# Supplementary material for: Bidirectional Two-Sample, Two-Step Mendelian Randomisation Study Reveals Mediating Role of Gut Microbiota Between Vitamin B Supplementation and Alzheimer’s Disease
Source: Nutrients. 2024 Nov 18;16(22):3929. doi: 10.3390/nu16223929 (PMC11597120; doi:10.3390/nu16223929)
Supplement: Supplementary file 1 [file nutrients-16-03929-s001.zip › Table S1.pdf]

SNP	other_all	effect_al	beta.expo	se.exposu	pval.expo	samplesiz	exposure	mr_keep.e	pval_orig	id.exposu	eaf.expos
rs1126173	C	T	-0.17087	0.036284	2.38E-06	4001	genus..Eubacteriumbrachygroup	TRUE	reported	U6XL4x	NA
rs9613196	A	T	-0.23872	0.052856	4.99E-06	3004	genus..Eubacteriumbrachygroup	TRUE	reported	U6XL4x	NA
rs6234877	C	T	-0.20148	0.043286	3.78E-06	3827	genus..Eubacteriumbrachygroup	TRUE	reported	U6XL4x	NA
rs2913110	T	C	0.105143	0.022942	4.56E-06	4001	genus..Eubacteriumbrachygroup	TRUE	reported	U6XL4x	NA
rs4862235	A	G	0.104806	0.022575	3.73E-06	4000	genus..Eubacteriumbrachygroup	TRUE	reported	U6XL4x	NA
rs1215142	G	A	0.101329	0.022732	9.27E-06	3854	genus..Eubacteriumbrachygroup	TRUE	reported	U6XL4x	NA
rs7319991	C	T	-0.23672	0.053133	8.16E-06	3008	genus..Eubacteriumbrachygroup	TRUE	reported	U6XL4x	NA
rs6591893	A	G	0.108225	0.024032	7.34E-06	3999	genus..Eubacteriumbrachygroup	TRUE	reported	U6XL4x	NA
rs720439	G	A	-0.11193	0.025125	7.03E-06	4001	genus..Eubacteriumbrachygroup	TRUE	reported	U6XL4x	NA
rs1313959	C	T	-0.146	0.032719	7.97E-06	3842	genus..Eubacteriumbrachygroup	TRUE	reported	U6XL4x	NA
rs1384962	G	A	0.120888	0.026626	6.99E-06	4001	genus..Eubacteriumbrachygroup	TRUE	reported	U6XL4x	NA
rs1715986	T	C	0.096223	0.016839	1.04E-08	17380	genus..Eubacteriumcoprostanol	TRUE	reported	RuIPL5	NA
rs9648214	C	T	-0.08287	0.016431	2.52E-07	17379	genus..Eubacteriumcoprostanol	TRUE	reported	RuIPL5	NA
rs1153257	C	G	0.078332	0.017472	2.62E-06	16555	genus..Eubacteriumcoprostanol	TRUE	reported	RuIPL5	NA
rs1290695	T	C	-0.05332	0.011593	4.35E-06	17380	genus..Eubacteriumcoprostanol	TRUE	reported	RuIPL5	NA
rs4076415	G	T	0.051515	0.011029	1.99E-06	16960	genus..Eubacteriumcoprostanol	TRUE	reported	RuIPL5	NA
rs6762473	A	C	0.052158	0.011235	4.26E-06	17377	genus..Eubacteriumcoprostanol	TRUE	reported	RuIPL5	NA
rs7310967	A	G	-0.0797	0.017228	3.66E-06	17379	genus..Eubacteriumcoprostanol	TRUE	reported	RuIPL5	NA
rs1044419	G	A	-0.05058	0.011346	5.98E-06	17380	genus..Eubacteriumcoprostanol	TRUE	reported	RuIPL5	NA
rs2644213	A	G	0.053885	0.012124	9.86E-06	17380	genus..Eubacteriumcoprostanol	TRUE	reported	RuIPL5	NA
rs1172085	T	C	0.063078	0.014448	9.26E-06	16960	genus..Eubacteriumcoprostanol	TRUE	reported	RuIPL5	NA
rs4717831	T	A	0.078678	0.017404	9.18E-06	16854	genus..Eubacteriumcoprostanol	TRUE	reported	RuIPL5	NA
rs6202443	T	C	-0.07697	0.01721	7.50E-06	17271	genus..Eubacteriumcoprostanol	TRUE	reported	RuIPL5	NA
rs7689892	A	G	0.123055	0.026637	4.79E-06	12197	genus..Eubacteriumcoprostanol	TRUE	reported	RuIPL5	NA
rs1105206	C	T	0.047783	0.010783	9.38E-06	17380	genus..Eubacteriumcoprostanol	TRUE	reported	RuIPL5	NA
rs7989514	C	T	-0.0641	0.014115	8.62E-06	16139	genus..Eubacteriumcoprostanol	TRUE	reported	RuIPL5	NA
rs4583233	C	A	0.06702	0.012812	2.84E-07	14327	genus..Eubacteriumeligensgrou	TRUE	reported	KgCYGO	NA
rs265534	G	T	-0.05638	0.012013	2.27E-06	14776	genus..Eubacteriumeligensgrou	TRUE	reported	KgCYGO	NA
rs7283919	G	C	0.155659	0.037561	3.14E-06	4926	genus..Eubacteriumeligensgrou	TRUE	reported	KgCYGO	NA
rs7460615	G	C	-0.19645	0.042565	4.25E-06	5381	genus..Eubacteriumeligensgrou	TRUE	reported	KgCYGO	NA
rs6923695	G	T	0.103268	0.022952	4.87E-06	12891	genus..Eubacteriumeligensgrou	TRUE	reported	KgCYGO	NA
rs5608021	T	C	0.123093	0.028274	9.14E-06	10699	genus..Eubacteriumeligensgrou	TRUE	reported	KgCYGO	NA

rs4888188	G	T	-0.05333	0.011913	7.74E-06	14771	genus..Eubacteriumeligensgrou	TRUE	reported	KgCYGO	NA
rs158115	C	G	0.091527	0.019061	9.74E-06	14678	genus..Eubacteriumeligensgrou	TRUE	reported	KgCYGO	NA
rs2200429	G	A	-0.08888	0.019848	5.30E-06	14761	genus..Eubacteriumeligensgrou	TRUE	reported	KgCYGO	NA
rs182318	A	G	-0.08249	0.019561	8.40E-06	14777	genus..Eubacteriumeligensgrou	TRUE	reported	KgCYGO	NA
rs1271905	G	A	0.091663	0.020818	7.12E-06	12799	genus..Eubacteriumeligensgrou	TRUE	reported	KgCYGO	NA
rs3771393	T	C	0.130842	0.026667	7.38E-07	3792	genus..Eubacteriumfissicatena	TRUE	reported	pRQoZI	NA
rs2733072	A	G	0.109644	0.022831	1.49E-06	3833	genus..Eubacteriumfissicatena	TRUE	reported	pRQoZI	NA
rs7104872	A	G	0.138612	0.029191	2.73E-06	3829	genus..Eubacteriumfissicatena	TRUE	reported	pRQoZI	NA
rs1512576	G	A	0.20951	0.045485	3.10E-06	3587	genus..Eubacteriumfissicatena	TRUE	reported	pRQoZI	NA
rs1187629	C	T	0.131469	0.028171	2.67E-06	3396	genus..Eubacteriumfissicatena	TRUE	reported	pRQoZI	NA
rs6934739	G	A	0.111463	0.025279	9.75E-06	3831	genus..Eubacteriumfissicatena	TRUE	reported	pRQoZI	NA
rs1014790	G	T	0.172263	0.039601	8.27E-06	3833	genus..Eubacteriumfissicatena	TRUE	reported	pRQoZI	NA
rs1768152	C	T	0.139489	0.031619	8.70E-06	3590	genus..Eubacteriumfissicatena	TRUE	reported	pRQoZI	NA
rs1181840	A	G	0.10585	0.023711	8.20E-06	3590	genus..Eubacteriumfissicatena	TRUE	reported	pRQoZI	NA
rs1311636	C	T	0.154124	0.029719	2.94E-07	10323	genus..Eubacteriumhalliigroup	TRUE	reported	5iKGmv	NA
rs949971	G	T	-0.05402	0.01161	3.29E-06	16566	genus..Eubacteriumhalliigroup	TRUE	reported	5iKGmv	NA
rs1079899	T	C	0.060166	0.012675	2.61E-06	16561	genus..Eubacteriumhalliigroup	TRUE	reported	5iKGmv	NA
rs6025419	G	A	-0.05228	0.011186	2.70E-06	16238	genus..Eubacteriumhalliigroup	TRUE	reported	5iKGmv	NA
rs1080811	C	A	-0.05047	0.010992	4.42E-06	16566	genus..Eubacteriumhalliigroup	TRUE	reported	5iKGmv	NA
rs7401858	T	C	0.208943	0.043822	3.70E-06	3039	genus..Eubacteriumhalliigroup	TRUE	reported	5iKGmv	NA
rs6550770	C	T	-0.19809	0.044354	4.82E-06	4279	genus..Eubacteriumhalliigroup	TRUE	reported	5iKGmv	NA
rs1050137	T	C	-0.11559	0.025249	5.42E-06	13824	genus..Eubacteriumhalliigroup	TRUE	reported	5iKGmv	NA
rs281379	G	A	-0.04995	0.011215	9.33E-06	16130	genus..Eubacteriumhalliigroup	TRUE	reported	5iKGmv	NA
rs7805609	T	G	-0.05074	0.011376	8.29E-06	16568	genus..Eubacteriumhalliigroup	TRUE	reported	5iKGmv	NA
rs1385318	G	A	0.153138	0.034946	5.43E-06	7710	genus..Eubacteriumhalliigroup	TRUE	reported	5iKGmv	NA
rs2858481	G	A	0.126115	0.026863	4.43E-06	11095	genus..Eubacteriumhalliigroup	TRUE	reported	5iKGmv	NA
rs1177481	C	T	-0.12658	0.028712	7.86E-06	10861	genus..Eubacteriumhalliigroup	TRUE	reported	5iKGmv	NA
rs1747425	A	G	0.081081	0.018457	9.45E-06	16460	genus..Eubacteriumhalliigroup	TRUE	reported	5iKGmv	NA
rs630939	T	C	-0.05089	0.011435	9.16E-06	16238	genus..Eubacteriumhalliigroup	TRUE	reported	5iKGmv	NA
rs1707406	C	T	-0.0814	0.018927	9.35E-06	15804	genus..Eubacteriumhalliigroup	TRUE	reported	5iKGmv	NA
rs3429706	G	A	-0.18691	0.034147	6.60E-08	3271	genus..Eubacteriumnodatumgrou	TRUE	reported	46hpnn	NA
rs1138936	T	C	-0.18511	0.040357	5.76E-06	3195	genus..Eubacteriumnodatumgrou	TRUE	reported	46hpnn	NA
rs7791082	T	C	0.201788	0.041357	9.05E-07	3271	genus..Eubacteriumnodatumgrou	TRUE	reported	46hpnn	NA

rs6184104	T	G	0.160621	0.034161	3.56E-06	3295	genus..Eubacteriumnodatumgrou	TRUE	reported 46hpnn	NA
rs9425984	C	T	-0.13023	0.029235	7.21E-06	3294	genus..Eubacteriumnodatumgrou	TRUE	reported 46hpnn	NA
rs1026362	T	C	0.193496	0.043902	8.91E-06	3080	genus..Eubacteriumnodatumgrou	TRUE	reported 46hpnn	NA
rs1100657	G	A	-0.11017	0.024593	7.99E-06	3295	genus..Eubacteriumnodatumgrou	TRUE	reported 46hpnn	NA
rs7880204	C	T	-0.12545	0.02753	6.84E-06	3294	genus..Eubacteriumnodatumgrou	TRUE	reported 46hpnn	NA
rs6818880	G	A	-0.11008	0.024596	7.83E-06	3291	genus..Eubacteriumnodatumgrou	TRUE	reported 46hpnn	NA
rs1045829	C	T	-0.18775	0.041969	8.37E-06	3271	genus..Eubacteriumnodatumgrou	TRUE	reported 46hpnn	NA
rs7827125	T	C	0.1223	0.027133	7.17E-06	3294	genus..Eubacteriumnodatumgrou	TRUE	reported 46hpnn	NA
rs1242377	T	G	0.14098	0.029503	2.63E-06	5321	genus..Eubacteriumoxidoreduce	TRUE	reported XqBDUu	NA
rs2973294	T	G	0.092356	0.019544	2.39E-06	5378	genus..Eubacteriumoxidoreduce	TRUE	reported XqBDUu	NA
rs3456113	A	G	0.216145	0.045992	2.51E-06	4186	genus..Eubacteriumoxidoreduce	TRUE	reported XqBDUu	NA
rs440215	T	C	0.093277	0.019529	1.65E-06	5377	genus..Eubacteriumoxidoreduce	TRUE	reported XqBDUu	NA
rs1425962	C	G	0.09061	0.020061	7.32E-06	5321	genus..Eubacteriumoxidoreduce	TRUE	reported XqBDUu	NA
rs1212990	A	C	0.089327	0.019833	5.80E-06	5377	genus..Eubacteriumoxidoreduce	TRUE	reported XqBDUu	NA
rs3539895	G	A	-0.09014	0.01746	5.40E-07	16621	genus..Eubacteriumrectalegrou	TRUE	reported HfXnfV	NA
rs1171514	C	G	-0.11314	0.024354	1.84E-06	13908	genus..Eubacteriumrectalegrou	TRUE	reported HfXnfV	NA
rs1696015	G	C	-0.1566	0.033623	4.10E-06	8334	genus..Eubacteriumrectalegrou	TRUE	reported HfXnfV	NA
rs314726	C	T	0.052882	0.010949	1.38E-06	16966	genus..Eubacteriumrectalegrou	TRUE	reported HfXnfV	NA
rs1079754	G	A	0.050318	0.010839	3.53E-06	17350	genus..Eubacteriumrectalegrou	TRUE	reported HfXnfV	NA
rs1024885	A	C	-0.05278	0.011346	4.21E-06	17348	genus..Eubacteriumrectalegrou	TRUE	reported HfXnfV	NA
rs3980709	T	A	-0.06227	0.014057	6.86E-06	17340	genus..Eubacteriumrectalegrou	TRUE	reported HfXnfV	NA
rs2884897	G	A	-0.12936	0.028902	6.44E-06	10813	genus..Eubacteriumrectalegrou	TRUE	reported HfXnfV	NA
rs1089208	G	C	-0.06355	0.014007	6.22E-06	17351	genus..Eubacteriumrectalegrou	TRUE	reported HfXnfV	NA
rs1436947	C	T	0.087003	0.019779	9.75E-06	15124	genus..Eubacteriumrectalegrou	TRUE	reported HfXnfV	NA
rs5942769	G	A	-0.0576	0.013085	5.37E-06	16613	genus..Eubacteriumrectalegrou	TRUE	reported HfXnfV	NA
rs6254723	G	A	0.053632	0.01202	9.90E-06	17347	genus..Eubacteriumrectalegrou	TRUE	reported HfXnfV	NA
rs5849841	T	C	-0.05173	0.01182	9.68E-06	17350	genus..Eubacteriumrectalegrou	TRUE	reported HfXnfV	NA
rs2116427	G	A	0.091146	0.018235	4.67E-07	7739	genus..Eubacteriumruminantium	TRUE	reported qrc4PH	NA
rs139749	T	C	-0.08454	0.017179	8.59E-07	7314	genus..Eubacteriumruminantium	TRUE	reported qrc4PH	NA
rs7283642	T	C	-0.13982	0.030069	2.62E-06	7314	genus..Eubacteriumruminantium	TRUE	reported qrc4PH	NA
rs2229917	G	A	0.153538	0.032392	2.16E-06	7050	genus..Eubacteriumruminantium	TRUE	reported qrc4PH	NA
rs1013172	C	A	-0.19983	0.041458	2.39E-06	5071	genus..Eubacteriumruminantium	TRUE	reported qrc4PH	NA
rs1689189	A	G	-0.17479	0.039057	2.38E-06	4753	genus..Eubacteriumruminantium	TRUE	reported qrc4PH	NA

rs7000472	G	A	-0.07623	0.016523	4.07E-06	7739	genus..Eubacteriumruminantium	TRUE	reported	qrc4PH	NA
rs1302546	C	T	-0.07371	0.016379	6.97E-06	7738	genus..Eubacteriumruminantium	TRUE	reported	qrc4PH	NA
rs2418654	T	C	-0.07489	0.016585	6.17E-06	7314	genus..Eubacteriumruminantium	TRUE	reported	qrc4PH	NA
rs6676699	T	G	-0.08881	0.019645	6.38E-06	7708	genus..Eubacteriumruminantium	TRUE	reported	qrc4PH	NA
rs7313962	C	A	-0.1151	0.024791	5.36E-06	7314	genus..Eubacteriumruminantium	TRUE	reported	qrc4PH	NA
rs1123758	A	T	0.143141	0.029376	5.82E-06	7123	genus..Eubacteriumruminantium	TRUE	reported	qrc4PH	NA
rs606117	G	A	0.083324	0.018056	4.82E-06	7263	genus..Eubacteriumruminantium	TRUE	reported	qrc4PH	NA
rs5734034	C	T	-0.09794	0.021217	4.93E-06	7314	genus..Eubacteriumruminantium	TRUE	reported	qrc4PH	NA
rs2817174	T	C	-0.07343	0.016369	7.87E-06	7710	genus..Eubacteriumruminantium	TRUE	reported	qrc4PH	NA
rs1092301	A	G	0.072644	0.016092	6.80E-06	7739	genus..Eubacteriumruminantium	TRUE	reported	qrc4PH	NA
rs1163798	T	G	-0.07326	0.016089	5.44E-06	7718	genus..Eubacteriumruminantium	TRUE	reported	qrc4PH	NA
rs1751947	T	C	0.107804	0.023398	4.70E-06	7710	genus..Eubacteriumruminantium	TRUE	reported	qrc4PH	NA
rs209813	A	G	-0.10349	0.023639	9.23E-06	7264	genus..Eubacteriumruminantium	TRUE	reported	qrc4PH	NA
rs6048195	T	A	-0.06036	0.011658	2.50E-07	14839	genus..Eubacteriumventriosumg	TRUE	reported	jDKzzg	NA
rs7361540	C	T	-0.09563	0.019343	9.54E-07	15146	genus..Eubacteriumventriosumg	TRUE	reported	jDKzzg	NA
rs5719956	C	T	0.078339	0.016026	7.97E-07	14913	genus..Eubacteriumventriosumg	TRUE	reported	jDKzzg	NA
rs1161769	G	A	-0.14335	0.028624	7.22E-07	11497	genus..Eubacteriumventriosumg	TRUE	reported	jDKzzg	NA
rs1688468	T	G	-0.09064	0.019191	1.74E-06	15289	genus..Eubacteriumventriosumg	TRUE	reported	jDKzzg	NA
rs1296451	A	G	0.05872	0.012345	2.07E-06	15289	genus..Eubacteriumventriosumg	TRUE	reported	jDKzzg	NA
rs876734	T	C	-0.06187	0.013253	2.89E-06	14913	genus..Eubacteriumventriosumg	TRUE	reported	jDKzzg	NA
rs3809430	C	T	-0.05482	0.011843	3.55E-06	15287	genus..Eubacteriumventriosumg	TRUE	reported	jDKzzg	NA
rs7278303	A	C	0.065893	0.014375	6.55E-06	15286	genus..Eubacteriumventriosumg	TRUE	reported	jDKzzg	NA
rs7384922	C	T	0.097571	0.022439	5.21E-06	14573	genus..Eubacteriumventriosumg	TRUE	reported	jDKzzg	NA
rs9316536	G	T	-0.08171	0.018312	7.84E-06	15289	genus..Eubacteriumventriosumg	TRUE	reported	jDKzzg	NA
rs3517927	T	C	-0.06273	0.013786	5.76E-06	15287	genus..Eubacteriumventriosumg	TRUE	reported	jDKzzg	NA
rs7825028	A	G	0.074976	0.01644	3.36E-06	14835	genus..Eubacteriumventriosumg	TRUE	reported	jDKzzg	NA
rs6674642	T	C	0.07515	0.016498	6.11E-06	14578	genus..Eubacteriumventriosumg	TRUE	reported	jDKzzg	NA
rs6704822	G	A	0.073918	0.01667	6.62E-06	15289	genus..Eubacteriumventriosumg	TRUE	reported	jDKzzg	NA
rs1308241	T	C	-0.07155	0.016137	9.56E-06	14501	genus..Eubacteriumventriosumg	TRUE	reported	jDKzzg	NA
rs6683035	A	T	0.052875	0.011797	6.87E-06	14913	genus..Eubacteriumventriosumg	TRUE	reported	jDKzzg	NA
rs1783003	A	G	-0.1606	0.031051	2.39E-07	9527	genus..Eubacteriumxylanophilu	TRUE	reported	e0PGqe	NA
rs1323907	A	G	0.068743	0.014273	1.82E-06	13307	genus..Eubacteriumxylanophilu	TRUE	reported	e0PGqe	NA
rs7958270	G	C	-0.09517	0.019997	2.41E-06	13256	genus..Eubacteriumxylanophilu	TRUE	reported	e0PGqe	NA

rs1298012	G	C	-0.10827	0.023826	5.02E-06	12039	genus..Eubacteriumxylanophilu	TRUE	reported	e0PGqe	NA
rs2012708	G	A	0.05732	0.012691	6.53E-06	13306	genus..Eubacteriumxylanophilu	TRUE	reported	e0PGqe	NA
rs2213117	G	T	0.08778	0.018901	4.21E-06	12878	genus..Eubacteriumxylanophilu	TRUE	reported	e0PGqe	NA
rs1014018	C	A	0.057679	0.012599	4.96E-06	12930	genus..Eubacteriumxylanophilu	TRUE	reported	e0PGqe	NA
rs1091720	C	A	0.061263	0.013092	3.15E-06	12930	genus..Eubacteriumxylanophilu	TRUE	reported	e0PGqe	NA
rs1121761	T	C	-0.11346	0.024576	3.33E-06	12199	genus..Eubacteriumxylanophilu	TRUE	reported	e0PGqe	NA
rs1999224	T	G	-0.09491	0.02035	3.75E-06	13309	genus..Eubacteriumxylanophilu	TRUE	reported	e0PGqe	NA
rs7558683	G	A	-0.1145	0.026345	9.39E-06	12550	genus..Eubacteriumxylanophilu	TRUE	reported	e0PGqe	NA
rs4654122	G	C	0.055351	0.012359	7.20E-06	13309	genus..Eubacteriumxylanophilu	TRUE	reported	e0PGqe	NA
rs2047242	G	A	-0.0676	0.013374	2.46E-07	13369	genus..Ruminococcusgauvreauii	TRUE	reported	W4L48e	NA
rs7138668	G	T	0.121037	0.02386	2.91E-07	11340	genus..Ruminococcusgauvreauii	TRUE	reported	W4L48e	NA
rs9870933	G	A	0.062164	0.012607	8.49E-07	13382	genus..Ruminococcusgauvreauii	TRUE	reported	W4L48e	NA
rs1391597	T	C	0.059028	0.012487	1.86E-06	13382	genus..Ruminococcusgauvreauii	TRUE	reported	W4L48e	NA
rs431418	G	A	-0.09474	0.021016	5.54E-06	13289	genus..Ruminococcusgauvreauii	TRUE	reported	W4L48e	NA
rs1093148	A	G	0.06101	0.013048	3.38E-06	13382	genus..Ruminococcusgauvreauii	TRUE	reported	W4L48e	NA
rs289410	A	G	-0.06549	0.01391	2.27E-06	13380	genus..Ruminococcusgauvreauii	TRUE	reported	W4L48e	NA
rs2105937	G	A	0.058022	0.012781	5.10E-06	13364	genus..Ruminococcusgauvreauii	TRUE	reported	W4L48e	NA
rs2166943	C	A	0.056697	0.01235	5.28E-06	13377	genus..Ruminococcusgauvreauii	TRUE	reported	W4L48e	NA
rs1253981	T	C	0.110654	0.024068	4.49E-06	11860	genus..Ruminococcusgauvreauii	TRUE	reported	W4L48e	NA
rs1207957	G	A	0.095532	0.021344	5.04E-06	12986	genus..Ruminococcusgauvreauii	TRUE	reported	W4L48e	NA
rs7380284	A	C	0.073681	0.016966	7.48E-06	13382	genus..Ruminococcusgauvreauii	TRUE	reported	W4L48e	NA
rs1318880	A	T	0.070942	0.015686	7.28E-06	12834	genus..Ruminococcusgauvreauii	TRUE	reported	W4L48e	NA
rs1316352	A	G	-0.12738	0.023388	5.61E-08	6326	genus..Ruminococcusgnavusgrou	TRUE	reported	ZdtCrp	NA
rs934940	C	A	-0.10504	0.022959	2.74E-06	6309	genus..Ruminococcusgnavusgrou	TRUE	reported	ZdtCrp	NA
rs9872758	C	T	0.084923	0.017664	1.66E-06	6398	genus..Ruminococcusgnavusgrou	TRUE	reported	ZdtCrp	NA
rs2909242	A	C	-0.091	0.018351	7.41E-07	6401	genus..Ruminococcusgnavusgrou	TRUE	reported	ZdtCrp	NA
rs3124783	G	A	-0.11599	0.02491	2.67E-06	6401	genus..Ruminococcusgnavusgrou	TRUE	reported	ZdtCrp	NA
rs6216703	C	T	0.185289	0.03963	3.50E-06	5194	genus..Ruminococcusgnavusgrou	TRUE	reported	ZdtCrp	NA
rs1298933	A	G	-0.08469	0.018794	7.12E-06	6401	genus..Ruminococcusgnavusgrou	TRUE	reported	ZdtCrp	NA
rs1213654	T	C	0.090195	0.019648	3.10E-06	6401	genus..Ruminococcusgnavusgrou	TRUE	reported	ZdtCrp	NA
rs7839908	C	T	0.144445	0.032662	6.63E-06	5944	genus..Ruminococcusgnavusgrou	TRUE	reported	ZdtCrp	NA
rs1159710	G	A	0.114673	0.025065	6.95E-06	6328	genus..Ruminococcusgnavusgrou	TRUE	reported	ZdtCrp	NA
rs1186464	C	T	-0.1398	0.031825	5.01E-06	6241	genus..Ruminococcusgnavusgrou	TRUE	reported	ZdtCrp	NA

rs4388134	T	C	-0.0905	0.020354	9.12E-06	6399	genus..Ruminococcusgnavusgrou	TRUE	reported	ZdtCrp	NA
rs3586662	C	T	-0.0612	0.010942	2.21E-08	17240	genus..Ruminococcustorquesgro	TRUE	reported	V38KXy	NA
rs773123	A	T	0.0824	0.017386	1.59E-06	17011	genus..Ruminococcustorquesgro	TRUE	reported	V38KXy	NA
rs1090429	G	A	-0.1678	0.038981	2.69E-06	3143	genus..Ruminococcustorquesgro	TRUE	reported	V38KXy	NA
rs8080469	A	G	0.049073	0.010704	3.50E-06	17798	genus..Ruminococcustorquesgro	TRUE	reported	V38KXy	NA
rs7703462	G	T	-0.15155	0.033588	6.07E-06	8567	genus..Ruminococcustorquesgro	TRUE	reported	V38KXy	NA
rs7313096	T	A	0.077037	0.016826	3.71E-06	17011	genus..Ruminococcustorquesgro	TRUE	reported	V38KXy	NA
rs1972694	A	T	-0.06141	0.013732	8.93E-06	17353	genus..Ruminococcustorquesgro	TRUE	reported	V38KXy	NA
rs1243463	G	A	0.074697	0.01534	2.77E-06	17353	genus..Ruminococcustorquesgro	TRUE	reported	V38KXy	NA
rs158487	G	A	0.053178	0.011634	7.52E-06	17798	genus..Ruminococcustorquesgro	TRUE	reported	V38KXy	NA
rs4073731	C	T	0.065195	0.014222	4.05E-06	17793	genus..Ruminococcustorquesgro	TRUE	reported	V38KXy	NA
rs6060376	G	C	0.124328	0.027529	5.87E-06	11700	genus..Ruminococcustorquesgro	TRUE	reported	V38KXy	NA
rs1315477	A	T	0.056336	0.012953	7.16E-06	17795	genus..Ruminococcustorquesgro	TRUE	reported	V38KXy	NA
rs1475330	C	T	0.052349	0.01182	8.13E-06	17353	genus..Ruminococcustorquesgro	TRUE	reported	V38KXy	NA
rs1096778	A	C	0.050799	0.011332	8.37E-06	17791	genus..Ruminococcustorquesgro	TRUE	reported	V38KXy	NA
rs8141465	G	A	0.048078	0.010744	9.65E-06	17791	genus..Ruminococcustorquesgro	TRUE	reported	V38KXy	NA
rs3458378	T	G	0.126596	0.026846	4.49E-06	7468	genus.Actinomyces.id.423.summ	TRUE	reported	dEtIVq	NA
rs2715439	T	C	0.074668	0.016482	6.27E-06	7468	genus.Actinomyces.id.423.summ	TRUE	reported	dEtIVq	NA
rs4146653	A	G	0.098522	0.021418	4.50E-06	7468	genus.Actinomyces.id.423.summ	TRUE	reported	dEtIVq	NA
rs3501110	G	A	0.232634	0.051204	6.34E-06	3515	genus.Actinomyces.id.423.summ	TRUE	reported	dEtIVq	NA
rs4073240	A	G	0.074969	0.016737	7.94E-06	7468	genus.Actinomyces.id.423.summ	TRUE	reported	dEtIVq	NA
rs7915461	C	T	0.18776	0.040164	5.92E-06	5282	genus.Actinomyces.id.423.summ	TRUE	reported	dEtIVq	NA
rs7131524	G	A	-0.09698	0.021925	9.83E-06	7468	genus.Actinomyces.id.423.summ	TRUE	reported	dEtIVq	NA
rs1078798	C	G	0.094316	0.021351	9.62E-06	7468	genus.Actinomyces.id.423.summ	TRUE	reported	dEtIVq	NA
rs7680684	T	C	-0.08338	0.01689	9.77E-07	8243	genus.Adlercreutzia.id.812.su	TRUE	reported	jm89hK	NA
rs2147798	G	C	0.092296	0.019212	1.40E-06	8243	genus.Adlercreutzia.id.812.su	TRUE	reported	jm89hK	NA
rs8007899	T	A	-0.11324	0.023259	1.57E-06	8243	genus.Adlercreutzia.id.812.su	TRUE	reported	jm89hK	NA
rs2717140	T	C	-0.11923	0.025108	2.05E-06	8243	genus.Adlercreutzia.id.812.su	TRUE	reported	jm89hK	NA
rs9490822	T	C	-0.07345	0.015579	2.54E-06	8243	genus.Adlercreutzia.id.812.su	TRUE	reported	jm89hK	NA
rs1252251	T	A	-0.10488	0.023469	4.41E-06	8187	genus.Adlercreutzia.id.812.su	TRUE	reported	jm89hK	NA
rs1323152	A	C	0.143237	0.031165	4.81E-06	8051	genus.Adlercreutzia.id.812.su	TRUE	reported	jm89hK	NA
rs6664405	C	T	-0.09531	0.021075	5.23E-06	8051	genus.Adlercreutzia.id.812.su	TRUE	reported	jm89hK	NA
rs1046175	G	C	0.112758	0.025599	6.36E-06	8051	genus.Adlercreutzia.id.812.su	TRUE	reported	jm89hK	NA

rs9915817	C	T	0.074922	0.016833	8.22E-06	8243	genus.Adlercreutzia.id.812.su	TRUE	reported	jm89hK	NA
rs5571920	A	G	-0.06992	0.015804	9.61E-06	8243	genus.Adlercreutzia.id.812.su	TRUE	reported	jm89hK	NA
rs1160440	T	C	-0.10252	0.023483	9.74E-06	8107	genus.Adlercreutzia.id.812.su	TRUE	reported	jm89hK	NA
rs1172925	C	T	0.075047	0.015018	6.58E-07	11860	genus.Akkermansia.id.4037.sum	TRUE	reported	RNRG8H	NA
rs4936098	G	A	0.064922	0.013593	1.10E-06	11857	genus.Akkermansia.id.4037.sum	TRUE	reported	RNRG8H	NA
rs9349825	G	A	-0.07034	0.014713	2.60E-06	11857	genus.Akkermansia.id.4037.sum	TRUE	reported	RNRG8H	NA
rs7454292	C	T	0.112623	0.023643	1.48E-06	11215	genus.Akkermansia.id.4037.sum	TRUE	reported	RNRG8H	NA
rs3995795	T	C	0.064118	0.0141	5.22E-06	11689	genus.Akkermansia.id.4037.sum	TRUE	reported	RNRG8H	NA
rs2602429	T	C	0.074535	0.01562	2.72E-06	11689	genus.Akkermansia.id.4037.sum	TRUE	reported	RNRG8H	NA
rs6177920	A	G	-0.07605	0.016777	6.32E-06	11590	genus.Akkermansia.id.4037.sum	TRUE	reported	RNRG8H	NA
rs1118434	C	G	0.065585	0.014224	4.06E-06	11863	genus.Akkermansia.id.4037.sum	TRUE	reported	RNRG8H	NA
rs4242783	A	G	0.068545	0.01477	3.00E-06	11689	genus.Akkermansia.id.4037.sum	TRUE	reported	RNRG8H	NA
rs1171071	G	A	0.204406	0.043163	3.01E-06	4974	genus.Akkermansia.id.4037.sum	TRUE	reported	RNRG8H	NA
rs1290852	A	G	0.061772	0.013095	2.26E-06	11862	genus.Akkermansia.id.4037.sum	TRUE	reported	RNRG8H	NA
rs1118626	C	T	0.09112	0.019675	3.39E-06	11620	genus.Akkermansia.id.4037.sum	TRUE	reported	RNRG8H	NA
rs941682	A	G	-0.0633	0.014378	9.17E-06	11808	genus.Akkermansia.id.4037.sum	TRUE	reported	RNRG8H	NA
rs6728111	C	G	0.063457	0.013785	3.92E-06	17090	genus.Alistipes.id.968.summar	TRUE	reported	JpIjF8	NA
rs6770535	G	T	-0.05324	0.011086	1.65E-06	17567	genus.Alistipes.id.968.summar	TRUE	reported	JpIjF8	NA
rs1176900	A	G	-0.05288	0.010939	1.45E-06	17571	genus.Alistipes.id.968.summar	TRUE	reported	JpIjF8	NA
rs7129639	A	C	-0.0525	0.010958	1.78E-06	17571	genus.Alistipes.id.968.summar	TRUE	reported	JpIjF8	NA
rs1689282	C	A	-0.05201	0.011394	5.28E-06	17090	genus.Alistipes.id.968.summar	TRUE	reported	JpIjF8	NA
rs1195829	G	A	-0.09812	0.021827	9.30E-06	16214	genus.Alistipes.id.968.summar	TRUE	reported	JpIjF8	NA
rs8130320	G	A	-0.049	0.010717	4.84E-06	17571	genus.Alistipes.id.968.summar	TRUE	reported	JpIjF8	NA
rs2875322	C	T	-0.05809	0.013143	8.78E-06	17459	genus.Alistipes.id.968.summar	TRUE	reported	JpIjF8	NA
rs2290844	T	C	0.081397	0.019163	9.10E-06	17571	genus.Alistipes.id.968.summar	TRUE	reported	JpIjF8	NA
rs2450745	C	A	-0.08052	0.01847	7.12E-06	17571	genus.Alistipes.id.968.summar	TRUE	reported	JpIjF8	NA
rs6257641	C	T	0.049265	0.010973	7.50E-06	17564	genus.Alistipes.id.968.summar	TRUE	reported	JpIjF8	NA
rs1107244	A	G	0.075856	0.017118	3.59E-06	17571	genus.Alistipes.id.968.summar	TRUE	reported	JpIjF8	NA
rs3441706	G	A	-0.0482	0.010687	7.01E-06	17570	genus.Alistipes.id.968.summar	TRUE	reported	JpIjF8	NA
rs1299074	T	C	-0.07766	0.017299	8.21E-06	16694	genus.Alistipes.id.968.summar	TRUE	reported	JpIjF8	NA
rs4810359	G	A	-0.06524	0.014616	7.50E-06	17380	genus.Alistipes.id.968.summar	TRUE	reported	JpIjF8	NA
rs602075	G	A	0.168974	0.029698	3.57E-08	3212	genus.Allisonella.id.2174.sum	TRUE	reported	Mcgo6i	NA
rs3577846	T	C	0.146679	0.029719	1.21E-06	3212	genus.Allisonella.id.2174.sum	TRUE	reported	Mcgo6i	NA

rs6742198	A	G	0.149152	0.031648	3.35E-06	3182	genus.Allisonella.id.2174.sum	TRUE	reported	Mcgo6i	NA
rs1901739	G	T	0.115769	0.024863	3.59E-06	3212	genus.Allisonella.id.2174.sum	TRUE	reported	Mcgo6i	NA
rs7690484	A	G	0.148523	0.033486	6.09E-06	3182	genus.Allisonella.id.2174.sum	TRUE	reported	Mcgo6i	NA
rs3511069	C	T	-0.14632	0.032085	5.72E-06	3212	genus.Allisonella.id.2174.sum	TRUE	reported	Mcgo6i	NA
rs7898615	G	T	0.167966	0.037359	8.87E-06	3212	genus.Allisonella.id.2174.sum	TRUE	reported	Mcgo6i	NA
rs594561	T	C	0.112231	0.025168	9.41E-06	3212	genus.Allisonella.id.2174.sum	TRUE	reported	Mcgo6i	NA
rs685403	C	G	-0.1752	0.040447	4.88E-06	3212	genus.Allisonella.id.2174.sum	TRUE	reported	Mcgo6i	NA
rs1267559	T	G	0.145669	0.029068	9.64E-07	3008	genus.Alloprevotella.id.961.s	TRUE	reported	jgA6vB	NA
rs5821216	G	A	-0.16151	0.03591	7.94E-06	3005	genus.Alloprevotella.id.961.s	TRUE	reported	jgA6vB	NA
rs3461920	A	G	-0.15604	0.034439	8.84E-06	3003	genus.Alloprevotella.id.961.s	TRUE	reported	jgA6vB	NA
rs1738063	T	A	0.125564	0.028041	9.27E-06	3009	genus.Alloprevotella.id.961.s	TRUE	reported	jgA6vB	NA
rs4680035	G	A	-0.1196	0.025945	4.99E-06	3007	genus.Alloprevotella.id.961.s	TRUE	reported	jgA6vB	NA
rs4364940	G	A	0.126418	0.028221	8.58E-06	3008	genus.Alloprevotella.id.961.s	TRUE	reported	jgA6vB	NA
rs2154444	G	T	0.138234	0.030896	8.37E-06	3009	genus.Alloprevotella.id.961.s	TRUE	reported	jgA6vB	NA
rs816292	C	T	-0.113	0.02204	2.64E-07	5017	genus.Anaerofilum.id.2053.sum	TRUE	reported	y5Jlko	NA
rs7959889	T	C	0.182586	0.035728	3.75E-07	4709	genus.Anaerofilum.id.2053.sum	TRUE	reported	y5Jlko	NA
rs4506496	A	G	0.10309	0.021286	1.49E-06	4935	genus.Anaerofilum.id.2053.sum	TRUE	reported	y5Jlko	NA
rs712981	C	A	0.10076	0.02029	6.83E-07	5051	genus.Anaerofilum.id.2053.sum	TRUE	reported	y5Jlko	NA
rs1710549	C	G	-0.19311	0.041119	1.57E-06	4856	genus.Anaerofilum.id.2053.sum	TRUE	reported	y5Jlko	NA
rs1079435	C	T	-0.09535	0.02006	2.23E-06	4994	genus.Anaerofilum.id.2053.sum	TRUE	reported	y5Jlko	NA
rs1709687	T	C	-0.12634	0.026892	2.86E-06	5051	genus.Anaerofilum.id.2053.sum	TRUE	reported	y5Jlko	NA
rs356049	A	G	0.132603	0.02898	6.56E-06	4994	genus.Anaerofilum.id.2053.sum	TRUE	reported	y5Jlko	NA
rs1563175	C	A	0.092383	0.020216	5.54E-06	5051	genus.Anaerofilum.id.2053.sum	TRUE	reported	y5Jlko	NA
rs1701273	G	T	0.090342	0.020024	7.24E-06	5051	genus.Anaerofilum.id.2053.sum	TRUE	reported	y5Jlko	NA
rs9299345	C	T	-0.1364	0.030237	8.04E-06	5051	genus.Anaerofilum.id.2053.sum	TRUE	reported	y5Jlko	NA
rs4244069	A	G	-0.14677	0.032659	9.81E-06	5030	genus.Anaerofilum.id.2053.sum	TRUE	reported	y5Jlko	NA
rs7193624	T	C	0.075064	0.015072	5.35E-07	16926	genus.Anaerostipes.id.1991.su	TRUE	reported	j7ybj6	NA
rs2804244	G	A	-0.05309	0.011098	2.04E-06	16926	genus.Anaerostipes.id.1991.su	TRUE	reported	j7ybj6	NA
rs6221570	A	G	0.064497	0.01367	1.98E-06	15550	genus.Anaerostipes.id.1991.su	TRUE	reported	j7ybj6	NA
rs2396460	C	T	-0.05127	0.010956	2.91E-06	16912	genus.Anaerostipes.id.1991.su	TRUE	reported	j7ybj6	NA
rs3900776	A	G	-0.11001	0.02363	2.75E-06	14694	genus.Anaerostipes.id.1991.su	TRUE	reported	j7ybj6	NA
rs2014785	C	T	0.051569	0.01122	4.68E-06	16445	genus.Anaerostipes.id.1991.su	TRUE	reported	j7ybj6	NA
rs6215762	C	T	0.088573	0.018555	1.45E-06	15215	genus.Anaerostipes.id.1991.su	TRUE	reported	j7ybj6	NA

rs6726833	A	C	-0.08775	0.018941	3.32E-06	16924	genus.Anaerostipes.id.1991.su	TRUE	reported	j7ybj6	NA
rs6474958	G	A	-0.05001	0.0112	6.74E-06	16918	genus.Anaerostipes.id.1991.su	TRUE	reported	j7ybj6	NA
rs6854026	C	T	-0.05085	0.010908	3.20E-06	16921	genus.Anaerostipes.id.1991.su	TRUE	reported	j7ybj6	NA
rs7873537	C	A	-0.13743	0.03053	5.33E-06	10360	genus.Anaerostipes.id.1991.su	TRUE	reported	j7ybj6	NA
rs7823228	C	G	-0.06211	0.013661	5.84E-06	16445	genus.Anaerostipes.id.1991.su	TRUE	reported	j7ybj6	NA
rs1337655	T	A	0.197389	0.045864	7.72E-06	3101	genus.Anaerostipes.id.1991.su	TRUE	reported	j7ybj6	NA
rs6098335	A	G	-0.05399	0.011658	4.42E-06	16344	genus.Anaerostipes.id.1991.su	TRUE	reported	j7ybj6	NA
rs1050206	G	A	0.083575	0.019202	7.94E-06	16825	genus.Anaerostipes.id.1991.su	TRUE	reported	j7ybj6	NA
rs6563550	C	T	0.087713	0.017674	2.35E-07	16566	genus.Anaerotruncus.id.2054.s	TRUE	reported	Dir815	NA
rs8005030	T	C	0.055445	0.011785	2.28E-06	16548	genus.Anaerotruncus.id.2054.s	TRUE	reported	Dir815	NA
rs4669806	T	G	0.057639	0.012299	2.42E-06	16566	genus.Anaerotruncus.id.2054.s	TRUE	reported	Dir815	NA
rs1015023	G	A	0.056709	0.012488	6.68E-06	16117	genus.Anaerotruncus.id.2054.s	TRUE	reported	Dir815	NA
rs1272208	T	G	-0.06117	0.012983	4.28E-06	16117	genus.Anaerotruncus.id.2054.s	TRUE	reported	Dir815	NA
rs1205680	G	C	0.077419	0.017718	6.13E-06	16010	genus.Anaerotruncus.id.2054.s	TRUE	reported	Dir815	NA
rs1101856	G	A	-0.15647	0.036603	6.14E-06	6278	genus.Anaerotruncus.id.2054.s	TRUE	reported	Dir815	NA
rs7675045	T	A	-0.04978	0.011057	6.92E-06	16566	genus.Anaerotruncus.id.2054.s	TRUE	reported	Dir815	NA
rs1431492	T	C	-0.0655	0.014619	7.36E-06	16560	genus.Anaerotruncus.id.2054.s	TRUE	reported	Dir815	NA
rs9347879	C	T	0.050618	0.011049	4.22E-06	16566	genus.Anaerotruncus.id.2054.s	TRUE	reported	Dir815	NA
rs1773473	C	T	0.066005	0.014908	7.43E-06	15927	genus.Anaerotruncus.id.2054.s	TRUE	reported	Dir815	NA
rs6494922	G	A	0.090311	0.020226	6.62E-06	16067	genus.Anaerotruncus.id.2054.s	TRUE	reported	Dir815	NA
rs3444943	C	A	-0.0497	0.01134	9.85E-06	16117	genus.Anaerotruncus.id.2054.s	TRUE	reported	Dir815	NA
rs7155595	A	C	0.053934	0.01189	7.55E-06	16553	genus.Anaerotruncus.id.2054.s	TRUE	reported	Dir815	NA
rs1154148	C	A	-0.14436	0.031752	6.83E-06	9273	genus.Anaerotruncus.id.2054.s	TRUE	reported	Dir815	NA
rs2704155	A	T	-0.10623	0.023481	5.42E-06	12419	genus.Anaerotruncus.id.2054.s	TRUE	reported	Dir815	NA
rs6795673	T	C	0.053857	0.010525	3.38E-07	18301	genus.Bacteroides.id.918.summ	TRUE	reported	UCCoB1	NA
rs2875721	A	T	0.08184	0.017032	1.29E-06	14833	genus.Bacteroides.id.918.summ	TRUE	reported	UCCoB1	NA
rs9507307	T	C	0.060446	0.012913	2.13E-06	18184	genus.Bacteroides.id.918.summ	TRUE	reported	UCCoB1	NA
rs6647497	T	G	0.081252	0.016448	6.81E-07	17405	genus.Bacteroides.id.918.summ	TRUE	reported	UCCoB1	NA
rs1158589	G	A	-0.07407	0.014763	1.80E-06	17821	genus.Bacteroides.id.918.summ	TRUE	reported	UCCoB1	NA
rs495004	G	C	-0.06075	0.012987	3.42E-06	17707	genus.Bacteroides.id.918.summ	TRUE	reported	UCCoB1	NA
rs1761998	G	T	0.088098	0.0187	2.69E-06	15801	genus.Bacteroides.id.918.summ	TRUE	reported	UCCoB1	NA
rs2023437	C	T	-0.07823	0.016763	5.02E-06	17484	genus.Bacteroides.id.918.summ	TRUE	reported	UCCoB1	NA
rs6671094	T	C	0.048804	0.010741	5.86E-06	17821	genus.Bacteroides.id.918.summ	TRUE	reported	UCCoB1	NA

rs1320758	G	A	-0.0592	0.013119	7.49E-06	17707	genus.Bacteroides.id.918.summ	TRUE	reported	UCCoB1	NA
rs2366421	A	T	-0.05282	0.011712	7.65E-06	18299	genus.Bacteroides.id.918.summ	TRUE	reported	UCCoB1	NA
rs1340391	C	T	-0.0592	0.013224	6.73E-06	18302	genus.Bacteroides.id.918.summ	TRUE	reported	UCCoB1	NA
rs2276875	G	A	-0.06972	0.013954	4.65E-07	13893	genus.Barnesiella.id.944.summ	TRUE	reported	9Px1Vs	NA
rs2428166	A	G	-0.16586	0.033731	8.51E-07	8091	genus.Barnesiella.id.944.summ	TRUE	reported	9Px1Vs	NA
rs6031689	T	C	-0.12148	0.025184	1.19E-06	12717	genus.Barnesiella.id.944.summ	TRUE	reported	9Px1Vs	NA
rs3517786	G	A	0.091713	0.019013	2.95E-06	13394	genus.Barnesiella.id.944.summ	TRUE	reported	9Px1Vs	NA
rs1324261	C	T	-0.05837	0.012318	2.29E-06	13945	genus.Barnesiella.id.944.summ	TRUE	reported	9Px1Vs	NA
rs7979532	G	A	-0.08186	0.017644	4.23E-06	13657	genus.Barnesiella.id.944.summ	TRUE	reported	9Px1Vs	NA
rs2057922	C	G	0.091524	0.019462	3.83E-06	14208	genus.Barnesiella.id.944.summ	TRUE	reported	9Px1Vs	NA
rs199035	A	G	0.055949	0.011972	3.00E-06	13945	genus.Barnesiella.id.944.summ	TRUE	reported	9Px1Vs	NA
rs6225133	G	A	-0.06908	0.014937	4.24E-06	13945	genus.Barnesiella.id.944.summ	TRUE	reported	9Px1Vs	NA
rs7745585	G	T	-0.08915	0.01956	3.16E-06	13394	genus.Barnesiella.id.944.summ	TRUE	reported	9Px1Vs	NA
rs1115555	C	T	0.095602	0.021296	8.92E-06	13383	genus.Barnesiella.id.944.summ	TRUE	reported	9Px1Vs	NA
rs1290971	T	C	-0.05507	0.012004	4.95E-06	14205	genus.Barnesiella.id.944.summ	TRUE	reported	9Px1Vs	NA
rs1132581	G	A	0.098992	0.021412	7.31E-06	13945	genus.Barnesiella.id.944.summ	TRUE	reported	9Px1Vs	NA
rs2847980	T	A	-0.09152	0.021	7.93E-06	12399	genus.Barnesiella.id.944.summ	TRUE	reported	9Px1Vs	NA
rs7618174	T	C	-0.07786	0.017164	6.78E-06	14201	genus.Barnesiella.id.944.summ	TRUE	reported	9Px1Vs	NA
rs7268484	C	T	-0.11437	0.025387	6.76E-06	12984	genus.Barnesiella.id.944.summ	TRUE	reported	9Px1Vs	NA
rs2841878	G	C	-0.07913	0.017496	6.48E-06	13698	genus.Barnesiella.id.944.summ	TRUE	reported	9Px1Vs	NA
rs182549	T	C	0.119703	0.012729	1.28E-20	14778	genus.Bifidobacterium.id.436.	TRUE	reported	DznpLI	NA
rs7570971	C	A	0.113335	0.012575	2.01E-19	14666	genus.Bifidobacterium.id.436.	TRUE	reported	DznpLI	NA
rs7322849	C	T	0.112428	0.020181	1.08E-08	14778	genus.Bifidobacterium.id.436.	TRUE	reported	DznpLI	NA
rs638382	C	T	-0.06509	0.012301	9.45E-08	14021	genus.Bifidobacterium.id.436.	TRUE	reported	DznpLI	NA
rs1961273	T	C	0.067404	0.013232	3.51E-07	14778	genus.Bifidobacterium.id.436.	TRUE	reported	DznpLI	NA
rs6430601	G	T	0.079626	0.017031	1.09E-06	14776	genus.Bifidobacterium.id.436.	TRUE	reported	DznpLI	NA
rs1084147	C	G	-0.06242	0.012944	1.65E-06	14777	genus.Bifidobacterium.id.436.	TRUE	reported	DznpLI	NA
rs1879087	T	C	0.105365	0.024053	1.45E-06	9066	genus.Bifidobacterium.id.436.	TRUE	reported	DznpLI	NA
rs5610866	C	T	0.072997	0.015793	2.44E-06	13916	genus.Bifidobacterium.id.436.	TRUE	reported	DznpLI	NA
rs2686790	C	T	0.070741	0.015793	7.50E-06	14778	genus.Bifidobacterium.id.436.	TRUE	reported	DznpLI	NA
rs4567981	A	T	0.056208	0.011792	1.93E-06	14666	genus.Bifidobacterium.id.436.	TRUE	reported	DznpLI	NA
rs2491158	A	G	0.071262	0.015983	8.05E-06	14021	genus.Bifidobacterium.id.436.	TRUE	reported	DznpLI	NA
rs7667185	G	C	-0.08461	0.0184	3.96E-06	13806	genus.Bifidobacterium.id.436.	TRUE	reported	DznpLI	NA

rs753440⁄	T	C	0.232354	0.050598	4.86E-06	3856	genus.Bifidobacterium.id.436.	TRUE	reported DznpLI	NA
rs540489	G	T	-0.06376	0.013875	5.19E-06	14561	genus.Bifidobacterium.id.436.	TRUE	reported DznpLI	NA
rs857444	T	C	0.055823	0.012122	3.57E-06	14777	genus.Bifidobacterium.id.436.	TRUE	reported DznpLI	NA
rs130206{	A	G	0.05627	0.012262	4.07E-06	14666	genus.Bifidobacterium.id.436.	TRUE	reported DznpLI	NA
rs120221{	A	G	0.061936	0.013894	8.00E-06	14776	genus.Bifidobacterium.id.436.	TRUE	reported DznpLI	NA
rs621817(	A	G	-0.06246	0.013121	2.17E-06	14774	genus.Bifidobacterium.id.436.	TRUE	reported DznpLI	NA
rs558887(	G	A	0.054632	0.012114	6.67E-06	14777	genus.Bifidobacterium.id.436.	TRUE	reported DznpLI	NA
rs574648(	C	T	-0.05362	0.01208	9.00E-06	14778	genus.Bifidobacterium.id.436.	TRUE	reported DznpLI	NA
rs737974(	G	T	-0.09536	0.020924	4.38E-06	14666	genus.Bifidobacterium.id.436.	TRUE	reported DznpLI	NA
rs124655(	G	A	0.053579	0.011822	6.27E-06	14778	genus.Bifidobacterium.id.436.	TRUE	reported DznpLI	NA
rs116261(	C	G	0.128112	0.026136	8.62E-07	11276	genus.Bilophila.id.3170.summa	TRUE	reported AuKXBx	NA
rs157122{	T	C	0.082683	0.017062	1.12E-06	12854	genus.Bilophila.id.3170.summa	TRUE	reported AuKXBx	NA
rs382702(	T	C	0.076648	0.016064	1.79E-06	12628	genus.Bilophila.id.3170.summa	TRUE	reported AuKXBx	NA
rs124117]	A	G	-0.06927	0.015015	4.24E-06	12856	genus.Bilophila.id.3170.summa	TRUE	reported AuKXBx	NA
rs780284]	A	C	0.06701	0.01377	1.77E-06	12856	genus.Bilophila.id.3170.summa	TRUE	reported AuKXBx	NA
rs801354]	T	A	-0.0572	0.012567	5.48E-06	12856	genus.Bilophila.id.3170.summa	TRUE	reported AuKXBx	NA
rs679329]	A	C	0.112729	0.024163	3.11E-06	11051	genus.Bilophila.id.3170.summa	TRUE	reported AuKXBx	NA
rs191770(	T	A	0.118509	0.026742	7.37E-06	10886	genus.Bilophila.id.3170.summa	TRUE	reported AuKXBx	NA
rs542415	C	T	-0.06136	0.013344	4.71E-06	12777	genus.Bilophila.id.3170.summa	TRUE	reported AuKXBx	NA
rs726768{	C	T	0.123209	0.026882	5.62E-06	11616	genus.Bilophila.id.3170.summa	TRUE	reported AuKXBx	NA
rs479812(	A	G	0.073288	0.016799	7.15E-06	12777	genus.Bilophila.id.3170.summa	TRUE	reported AuKXBx	NA
rs196992⁄	A	G	0.056459	0.012693	9.07E-06	12777	genus.Bilophila.id.3170.summa	TRUE	reported AuKXBx	NA
rs601789{	A	G	-0.06248	0.014148	8.06E-06	12777	genus.Bilophila.id.3170.summa	TRUE	reported AuKXBx	NA
rs272849]	T	G	-0.06274	0.013946	6.33E-06	12856	genus.Bilophila.id.3170.summa	TRUE	reported AuKXBx	NA
rs271334(	T	A	0.061723	0.014017	8.63E-06	12853	genus.Bilophila.id.3170.summa	TRUE	reported AuKXBx	NA
rs989999(	G	A	-0.1027	0.023388	9.07E-06	12088	genus.Bilophila.id.3170.summa	TRUE	reported AuKXBx	NA
rs110694{	C	T	-0.06809	0.015503	7.72E-06	12856	genus.Bilophila.id.3170.summa	TRUE	reported AuKXBx	NA
rs111499⁄	T	C	0.117605	0.02339	1.04E-06	16255	genus.Blautia.id.1992.summary	TRUE	reported AtjNL3	NA
rs124530(	T	C	0.062533	0.012999	1.26E-06	17815	genus.Blautia.id.1992.summary	TRUE	reported AtjNL3	NA
rs115043(	A	G	-0.20661	0.043992	5.19E-06	4902	genus.Blautia.id.1992.summary	TRUE	reported AtjNL3	NA
rs677943⁄	T	C	0.060171	0.012344	1.00E-06	18158	genus.Blautia.id.1992.summary	TRUE	reported AtjNL3	NA
rs117001⁄	C	T	0.196414	0.044112	8.84E-06	4965	genus.Blautia.id.1992.summary	TRUE	reported AtjNL3	NA
rs729735{	G	A	0.125169	0.026541	1.74E-06	12764	genus.Blautia.id.1992.summary	TRUE	reported AtjNL3	NA

rs4926264	C	T	0.082621	0.017824	5.10E-06	18276	genus.Blautia.id.1992.summary	TRUE	reported	AtjNL3	NA
rs7860714	G	A	-0.05022	0.010985	4.09E-06	18162	genus.Blautia.id.1992.summary	TRUE	reported	AtjNL3	NA
rs682885	G	A	-0.04934	0.010736	4.49E-06	18274	genus.Blautia.id.1992.summary	TRUE	reported	AtjNL3	NA
rs2788271	G	T	-0.05756	0.013349	7.16E-06	18276	genus.Blautia.id.1992.summary	TRUE	reported	AtjNL3	NA
rs1132713	T	C	0.078268	0.017212	6.85E-06	17465	genus.Blautia.id.1992.summary	TRUE	reported	AtjNL3	NA
rs3005511	G	A	0.050108	0.011077	6.19E-06	18276	genus.Blautia.id.1992.summary	TRUE	reported	AtjNL3	NA
rs1689204	C	T	-0.06226	0.01418	8.82E-06	18276	genus.Blautia.id.1992.summary	TRUE	reported	AtjNL3	NA
rs5622123	C	T	0.082803	0.01674	7.62E-07	17133	genus.Butyricicoccus.id.2055.	TRUE	reported	hAqODa	NA
rs2017189	T	G	-0.0507	0.011024	3.87E-06	17133	genus.Butyricicoccus.id.2055.	TRUE	reported	hAqODa	NA
rs7322368	C	T	0.081573	0.018317	5.52E-06	17134	genus.Butyricicoccus.id.2055.	TRUE	reported	hAqODa	NA
rs7523876	A	T	0.061942	0.013994	6.80E-06	17022	genus.Butyricicoccus.id.2055.	TRUE	reported	hAqODa	NA
rs1258579	C	T	-0.26221	0.056473	5.79E-06	3135	genus.Butyricicoccus.id.2055.	TRUE	reported	hAqODa	NA
rs6247807	G	T	0.224039	0.049496	5.94E-06	4007	genus.Butyricicoccus.id.2055.	TRUE	reported	hAqODa	NA
rs4962426	T	G	0.061422	0.013598	7.38E-06	16889	genus.Butyricicoccus.id.2055.	TRUE	reported	hAqODa	NA
rs1008420	G	A	0.05497	0.012356	8.59E-06	17135	genus.Butyricicoccus.id.2055.	TRUE	reported	hAqODa	NA
rs1203471	G	A	0.07012	0.015821	9.58E-06	16237	genus.Butyricicoccus.id.2055.	TRUE	reported	hAqODa	NA
rs1130546	A	G	-0.14488	0.027457	1.74E-07	9949	genus.Butyricimonas.id.945.su	TRUE	reported	gm7y1i	NA
rs6239030	C	T	-0.0873	0.017489	7.42E-07	10634	genus.Butyricimonas.id.945.su	TRUE	reported	gm7y1i	NA
rs7083431	C	A	0.070354	0.014444	8.85E-07	10737	genus.Butyricimonas.id.945.su	TRUE	reported	gm7y1i	NA
rs2642760	C	G	0.071336	0.01454	8.58E-07	10634	genus.Butyricimonas.id.945.su	TRUE	reported	gm7y1i	NA
rs782080	A	T	0.065349	0.013797	2.23E-06	10733	genus.Butyricimonas.id.945.su	TRUE	reported	gm7y1i	NA
rs1862649	A	G	0.113124	0.024818	4.76E-06	10123	genus.Butyricimonas.id.945.su	TRUE	reported	gm7y1i	NA
rs1230403	A	G	-0.08628	0.019685	6.70E-06	10658	genus.Butyricimonas.id.945.su	TRUE	reported	gm7y1i	NA
rs1701950	C	G	-0.10506	0.024105	7.68E-06	9875	genus.Butyricimonas.id.945.su	TRUE	reported	gm7y1i	NA
rs7142862	T	G	-0.13319	0.028991	4.80E-06	9776	genus.Butyricimonas.id.945.su	TRUE	reported	gm7y1i	NA
rs7845336	G	A	-0.14948	0.032707	4.06E-06	8207	genus.Butyricimonas.id.945.su	TRUE	reported	gm7y1i	NA
rs326049	G	C	0.075969	0.016762	8.19E-06	10557	genus.Butyricimonas.id.945.su	TRUE	reported	gm7y1i	NA
rs6213033	A	G	-0.07326	0.015832	3.90E-06	10557	genus.Butyricimonas.id.945.su	TRUE	reported	gm7y1i	NA
rs9657374	T	C	0.068072	0.014817	4.50E-06	10737	genus.Butyricimonas.id.945.su	TRUE	reported	gm7y1i	NA
rs1245876	C	A	0.122037	0.026959	6.37E-06	10383	genus.Butyricimonas.id.945.su	TRUE	reported	gm7y1i	NA
rs2114713	T	G	0.062721	0.0139	6.88E-06	10734	genus.Butyricimonas.id.945.su	TRUE	reported	gm7y1i	NA
rs270727	C	G	-0.06942	0.01508	5.38E-06	10634	genus.Butyricimonas.id.945.su	TRUE	reported	gm7y1i	NA
rs7281452	G	A	0.066415	0.014977	8.25E-06	10736	genus.Butyricimonas.id.945.su	TRUE	reported	gm7y1i	NA

rs1122883	G	A	0.135265	0.029841	6.55E-06	8974	genus.Butyricimonas.id.945.su	TRUE	reported	gm7y1i	NA
rs7272366	T	C	0.224078	0.044934	7.86E-07	3073	genus.Butyrivibrio.id.1993.su	TRUE	reported	pJ5mvL	NA
rs7412979	G	C	0.186779	0.038911	1.71E-06	3480	genus.Butyrivibrio.id.1993.su	TRUE	reported	pJ5mvL	NA
rs7752361	G	A	-0.11923	0.023997	7.69E-07	3481	genus.Butyrivibrio.id.1993.su	TRUE	reported	pJ5mvL	NA
rs4537857	C	T	-0.12459	0.026099	1.80E-06	3482	genus.Butyrivibrio.id.1993.su	TRUE	reported	pJ5mvL	NA
rs1176167	C	T	0.154775	0.032154	2.20E-06	3482	genus.Butyrivibrio.id.1993.su	TRUE	reported	pJ5mvL	NA
rs1694133	T	C	0.127488	0.026794	1.53E-06	3482	genus.Butyrivibrio.id.1993.su	TRUE	reported	pJ5mvL	NA
rs7735620	C	T	0.216874	0.04833	6.66E-06	3379	genus.Butyrivibrio.id.1993.su	TRUE	reported	pJ5mvL	NA
rs7462218	G	A	-0.20103	0.04282	2.46E-06	3478	genus.Butyrivibrio.id.1993.su	TRUE	reported	pJ5mvL	NA
rs7763512	A	G	0.119846	0.025337	3.11E-06	3259	genus.Butyrivibrio.id.1993.su	TRUE	reported	pJ5mvL	NA
rs1428558	G	A	0.205013	0.045714	6.86E-06	3234	genus.Butyrivibrio.id.1993.su	TRUE	reported	pJ5mvL	NA
rs9349693	G	A	0.117973	0.025986	5.55E-06	3482	genus.Butyrivibrio.id.1993.su	TRUE	reported	pJ5mvL	NA
rs486484	G	A	-0.10835	0.024001	6.61E-06	3456	genus.Butyrivibrio.id.1993.su	TRUE	reported	pJ5mvL	NA
rs1716323	A	G	0.140987	0.030912	5.51E-06	3482	genus.Butyrivibrio.id.1993.su	TRUE	reported	pJ5mvL	NA
rs4928024	G	A	-0.17471	0.038933	8.19E-06	3481	genus.Butyrivibrio.id.1993.su	TRUE	reported	pJ5mvL	NA
rs1007475	T	G	0.118065	0.026127	7.92E-06	3482	genus.Butyrivibrio.id.1993.su	TRUE	reported	pJ5mvL	NA
rs1693406	C	T	-0.13378	0.029944	8.86E-06	3482	genus.Butyrivibrio.id.1993.su	TRUE	reported	pJ5mvL	NA
rs4294381	C	T	0.112197	0.023186	1.37E-06	6145	genus.CandidatusSoleaferrea.i	TRUE	reported	tj0De1	NA
rs1009036	G	A	-0.08344	0.018099	4.17E-06	6145	genus.CandidatusSoleaferrea.i	TRUE	reported	tj0De1	NA
rs4678258	C	T	0.098614	0.021565	5.53E-06	6101	genus.CandidatusSoleaferrea.i	TRUE	reported	tj0De1	NA
rs1010878	G	A	-0.09283	0.019981	3.64E-06	5999	genus.CandidatusSoleaferrea.i	TRUE	reported	tj0De1	NA
rs386526	G	C	0.081846	0.018009	8.33E-06	6144	genus.CandidatusSoleaferrea.i	TRUE	reported	tj0De1	NA
rs3615514	T	C	0.104966	0.0241	5.41E-06	5958	genus.CandidatusSoleaferrea.i	TRUE	reported	tj0De1	NA
rs6881988	C	G	-0.08191	0.018185	9.23E-06	6140	genus.CandidatusSoleaferrea.i	TRUE	reported	tj0De1	NA
rs1115315	C	G	-0.12805	0.028545	4.42E-06	6101	genus.CandidatusSoleaferrea.i	TRUE	reported	tj0De1	NA
rs1080913	C	T	0.083486	0.018243	5.47E-06	5999	genus.CandidatusSoleaferrea.i	TRUE	reported	tj0De1	NA
rs9973954	G	A	0.089208	0.01954	5.95E-06	6145	genus.CandidatusSoleaferrea.i	TRUE	reported	tj0De1	NA
rs6489992	G	A	-0.08404	0.018703	7.89E-06	6142	genus.CandidatusSoleaferrea.i	TRUE	reported	tj0De1	NA
rs6494306	G	A	-0.09693	0.021422	5.80E-06	6145	genus.CandidatusSoleaferrea.i	TRUE	reported	tj0De1	NA
rs1250023	T	A	0.081385	0.01827	7.68E-06	6145	genus.CandidatusSoleaferrea.i	TRUE	reported	tj0De1	NA
rs7400877	C	T	-0.09511	0.02128	9.29E-06	6145	genus.CandidatusSoleaferrea.i	TRUE	reported	tj0De1	NA
rs2193878	A	T	0.228255	0.050924	9.46E-06	3088	genus.CandidatusSoleaferrea.i	TRUE	reported	tj0De1	NA
rs830149	G	C	0.184647	0.039724	9.58E-06	5065	genus.CandidatusSoleaferrea.i	TRUE	reported	tj0De1	NA

rs1240491	T	C	0.140717	0.030414	2.80E-06	3239	genus.Catenibacterium.id.2153	TRUE	reported	MDxzu4	NA
rs772851(	A	G	-0.16183	0.035345	3.63E-06	3210	genus.Catenibacterium.id.2153	TRUE	reported	MDxzu4	NA
rs7312829	G	A	0.129726	0.028456	4.29E-06	3239	genus.Catenibacterium.id.2153	TRUE	reported	MDxzu4	NA
rs212393	A	G	-0.13525	0.028621	3.62E-06	3239	genus.Catenibacterium.id.2153	TRUE	reported	MDxzu4	NA
rs7742829	T	C	0.11411	0.02511	5.61E-06	3239	genus.Catenibacterium.id.2153	TRUE	reported	MDxzu4	NA
rs999354	A	T	0.058025	0.011719	7.01E-07	16324	genus.ChristensenellaceaeR.7g	TRUE	reported	tHvTuu	NA
rs7852137	T	C	0.124992	0.02748	5.61E-06	12605	genus.ChristensenellaceaeR.7g	TRUE	reported	tHvTuu	NA
rs6246712	T	C	0.114108	0.025198	3.25E-06	12719	genus.ChristensenellaceaeR.7g	TRUE	reported	tHvTuu	NA
rs1708179	G	A	-0.09043	0.020425	3.34E-06	16324	genus.ChristensenellaceaeR.7g	TRUE	reported	tHvTuu	NA
rs6213281	G	A	-0.08289	0.017962	5.67E-06	15066	genus.ChristensenellaceaeR.7g	TRUE	reported	tHvTuu	NA
rs6095466	G	T	0.049815	0.011079	7.13E-06	16324	genus.ChristensenellaceaeR.7g	TRUE	reported	tHvTuu	NA
rs1046125	G	A	-0.0552	0.012207	6.51E-06	15987	genus.ChristensenellaceaeR.7g	TRUE	reported	tHvTuu	NA
rs7915007	A	C	0.121547	0.027098	9.42E-06	11994	genus.ChristensenellaceaeR.7g	TRUE	reported	tHvTuu	NA
rs6219026	C	A	0.095839	0.021472	8.74E-06	15274	genus.ChristensenellaceaeR.7g	TRUE	reported	tHvTuu	NA
rs892686	G	A	0.05141	0.011136	3.97E-06	16325	genus.ChristensenellaceaeR.7g	TRUE	reported	tHvTuu	NA
rs7395201	T	C	-0.08622	0.019435	8.46E-06	15274	genus.ChristensenellaceaeR.7g	TRUE	reported	tHvTuu	NA
rs550843	C	T	-0.07832	0.016921	2.05E-06	14102	genus.Clostridiumsensustricto	TRUE	reported	pIf6Xc	NA
rs2795528	A	G	-0.18431	0.03922	2.72E-06	6366	genus.Clostridiumsensustricto	TRUE	reported	pIf6Xc	NA
rs2817172	T	C	0.058139	0.012449	2.77E-06	13658	genus.Clostridiumsensustricto	TRUE	reported	pIf6Xc	NA
rs115807(	G	A	-0.22736	0.049258	4.32E-06	4229	genus.Clostridiumsensustricto	TRUE	reported	pIf6Xc	NA
rs1168472	T	C	0.110021	0.024603	4.58E-06	12910	genus.Clostridiumsensustricto	TRUE	reported	pIf6Xc	NA
rs123415(	A	G	0.081072	0.018012	4.82E-06	14118	genus.Clostridiumsensustricto	TRUE	reported	pIf6Xc	NA
rs1158602	T	A	0.110967	0.02505	8.85E-06	13095	genus.Clostridiumsensustricto	TRUE	reported	pIf6Xc	NA
rs112644(	A	G	-0.13906	0.033445	7.76E-06	7285	genus.Clostridiumsensustricto	TRUE	reported	pIf6Xc	NA
rs1249033	G	C	-0.06169	0.013758	7.49E-06	14121	genus.Clostridiumsensustricto	TRUE	reported	pIf6Xc	NA
rs9541268	A	C	0.09597	0.019731	8.79E-07	12914	genus.Collinsella.id.815.summ	TRUE	reported	9RY35g	NA
rs2671662	G	C	-0.05678	0.011945	2.22E-06	14334	genus.Collinsella.id.815.summ	TRUE	reported	9RY35g	NA
rs7305225	A	G	0.093018	0.020266	1.72E-06	14334	genus.Collinsella.id.815.summ	TRUE	reported	9RY35g	NA
rs210351(	A	G	0.078655	0.016822	2.42E-06	14334	genus.Collinsella.id.815.summ	TRUE	reported	9RY35g	NA
rs7567279	G	A	-0.10891	0.024052	6.14E-06	12601	genus.Collinsella.id.815.summ	TRUE	reported	9RY35g	NA
rs1089067	C	T	-0.05374	0.011885	6.52E-06	14334	genus.Collinsella.id.815.summ	TRUE	reported	9RY35g	NA
rs129211(	T	A	0.056376	0.012702	8.23E-06	14334	genus.Collinsella.id.815.summ	TRUE	reported	9RY35g	NA
rs5941478	G	C	0.066887	0.014977	9.15E-06	14334	genus.Collinsella.id.815.summ	TRUE	reported	9RY35g	NA

rs6210263	G	A	-0.06963	0.015332	8.31E-06	14334	genus.Collinsella.id.815.summ	TRUE	reported	9RY35g	NA
rs6244887	A	C	-0.05403	0.012032	6.78E-06	14334	genus.Collinsella.id.815.summ	TRUE	reported	9RY35g	NA
rs1496620	C	T	-0.07223	0.01616	6.78E-06	14334	genus.Collinsella.id.815.summ	TRUE	reported	9RY35g	NA
rs1498075	A	C	-0.10426	0.023584	7.10E-06	13473	genus.Collinsella.id.815.summ	TRUE	reported	9RY35g	NA
rs1159728	T	G	-0.05379	0.012055	9.38E-06	14334	genus.Collinsella.id.815.summ	TRUE	reported	9RY35g	NA
rs305411	G	A	0.129218	0.026452	1.01E-06	6777	genus.Coprobacter.id.949.summ	TRUE	reported	Psu1a4	NA
rs3828477	T	G	-0.09122	0.019569	2.89E-06	6798	genus.Coprobacter.id.949.summ	TRUE	reported	Psu1a4	NA
rs5567235	A	T	-0.19335	0.041381	2.74E-06	4903	genus.Coprobacter.id.949.summ	TRUE	reported	Psu1a4	NA
rs1436629	T	C	0.253264	0.054014	3.07E-06	3083	genus.Coprobacter.id.949.summ	TRUE	reported	Psu1a4	NA
rs213863	T	C	-0.08874	0.018838	2.35E-06	6798	genus.Coprobacter.id.949.summ	TRUE	reported	Psu1a4	NA
rs7282140	C	T	-0.14737	0.031998	4.76E-06	6177	genus.Coprobacter.id.949.summ	TRUE	reported	Psu1a4	NA
rs1153234	T	C	-0.10393	0.022683	5.71E-06	6364	genus.Coprobacter.id.949.summ	TRUE	reported	Psu1a4	NA
rs1299605	C	A	0.092182	0.02094	8.08E-06	6570	genus.Coprobacter.id.949.summ	TRUE	reported	Psu1a4	NA
rs1268460	C	T	0.100827	0.022037	6.10E-06	6364	genus.Coprobacter.id.949.summ	TRUE	reported	Psu1a4	NA
rs5011652	C	G	0.08996	0.020001	5.51E-06	6783	genus.Coprobacter.id.949.summ	TRUE	reported	Psu1a4	NA
rs7491952	A	G	0.125703	0.02763	5.76E-06	6387	genus.Coprobacter.id.949.summ	TRUE	reported	Psu1a4	NA
rs2840269	C	T	0.110695	0.02512	9.56E-06	6387	genus.Coprobacter.id.949.summ	TRUE	reported	Psu1a4	NA
rs189356	A	G	0.078113	0.017192	6.26E-06	6763	genus.Coprobacter.id.949.summ	TRUE	reported	Psu1a4	NA
rs7600161	G	C	0.21591	0.049302	9.13E-06	3385	genus.Coprobacter.id.949.summ	TRUE	reported	Psu1a4	NA
rs4277593	A	G	-0.05856	0.010991	1.14E-07	16651	genus.Coprococcus1.id.11301.s	TRUE	reported	fnhMKP	NA
rs5640561	G	A	-0.08963	0.018651	1.57E-06	16379	genus.Coprococcus1.id.11301.s	TRUE	reported	fnhMKP	NA
rs7410191	C	T	-0.0719	0.014465	1.03E-06	16732	genus.Coprococcus1.id.11301.s	TRUE	reported	fnhMKP	NA
rs1010560	A	C	0.058023	0.012272	1.96E-06	16723	genus.Coprococcus1.id.11301.s	TRUE	reported	fnhMKP	NA
rs7303172	C	T	0.167591	0.035522	1.98E-06	7206	genus.Coprococcus1.id.11301.s	TRUE	reported	fnhMKP	NA
rs1519491	C	T	0.04992	0.011356	8.95E-06	16733	genus.Coprococcus1.id.11301.s	TRUE	reported	fnhMKP	NA
rs1576241	G	A	-0.05103	0.010953	3.33E-06	16729	genus.Coprococcus1.id.11301.s	TRUE	reported	fnhMKP	NA
rs7316707	C	T	0.057314	0.012757	8.57E-06	16733	genus.Coprococcus1.id.11301.s	TRUE	reported	fnhMKP	NA
rs1279489	T	G	0.09033	0.019721	4.92E-06	15186	genus.Coprococcus1.id.11301.s	TRUE	reported	fnhMKP	NA
rs946513	T	C	0.205899	0.046002	8.62E-06	4310	genus.Coprococcus1.id.11301.s	TRUE	reported	fnhMKP	NA
rs1762123	T	C	-0.08915	0.019855	8.01E-06	16724	genus.Coprococcus1.id.11301.s	TRUE	reported	fnhMKP	NA
rs7784490	G	C	-0.05185	0.011314	4.55E-06	16733	genus.Coprococcus1.id.11301.s	TRUE	reported	fnhMKP	NA
rs1288605	C	G	-0.05218	0.011805	8.01E-06	16633	genus.Coprococcus1.id.11301.s	TRUE	reported	fnhMKP	NA
rs2907920	G	A	0.056117	0.012682	7.65E-06	16040	genus.Coprococcus1.id.11301.s	TRUE	reported	fnhMKP	NA

rs5993692	T	A	0.117085	0.023399	9.38E-07	10931	genus.Coprococcus2.id.11302.s	TRUE	reported	lbheQ2	NA
rs6677933	T	C	-0.08044	0.016422	1.19E-06	11483	genus.Coprococcus2.id.11302.s	TRUE	reported	lbheQ2	NA
rs1958519	A	T	0.066529	0.013856	1.58E-06	11009	genus.Coprococcus2.id.11302.s	TRUE	reported	lbheQ2	NA
rs7268032	C	T	-0.06494	0.013919	2.27E-06	11009	genus.Coprococcus2.id.11302.s	TRUE	reported	lbheQ2	NA
rs2482516	T	C	0.075441	0.016462	4.72E-06	11483	genus.Coprococcus2.id.11302.s	TRUE	reported	lbheQ2	NA
rs3589011	G	A	-0.06654	0.014766	8.26E-06	11478	genus.Coprococcus2.id.11302.s	TRUE	reported	lbheQ2	NA
rs1012134	G	C	0.092581	0.021946	8.31E-06	11457	genus.Coprococcus2.id.11302.s	TRUE	reported	lbheQ2	NA
rs9426473	G	A	0.072736	0.016166	6.31E-06	11486	genus.Coprococcus2.id.11302.s	TRUE	reported	lbheQ2	NA
rs6894272	C	T	-0.11346	0.0253	9.53E-06	11001	genus.Coprococcus2.id.11302.s	TRUE	reported	lbheQ2	NA
rs1263407	C	T	0.073649	0.016492	9.95E-06	11457	genus.Coprococcus2.id.11302.s	TRUE	reported	lbheQ2	NA
rs6182351	C	A	-0.09554	0.021572	6.68E-06	10980	genus.Coprococcus2.id.11302.s	TRUE	reported	lbheQ2	NA
rs1007005	G	A	0.059431	0.013538	7.65E-06	11473	genus.Coprococcus2.id.11302.s	TRUE	reported	lbheQ2	NA
rs8100692	C	T	0.057741	0.011348	4.16E-07	15829	genus.Coprococcus3.id.11303.s	TRUE	reported	IPHJjg	NA
rs6248198	C	G	-0.05824	0.011505	4.18E-07	15472	genus.Coprococcus3.id.11303.s	TRUE	reported	IPHJjg	NA
rs178271	C	T	0.145257	0.029424	7.81E-07	10794	genus.Coprococcus3.id.11303.s	TRUE	reported	IPHJjg	NA
rs1339439	T	C	-0.07093	0.015092	2.20E-06	15834	genus.Coprococcus3.id.11303.s	TRUE	reported	IPHJjg	NA
rs1108034	T	C	0.051692	0.011305	4.79E-06	15835	genus.Coprococcus3.id.11303.s	TRUE	reported	IPHJjg	NA
rs7521171	A	G	-0.05964	0.012928	4.32E-06	15472	genus.Coprococcus3.id.11303.s	TRUE	reported	IPHJjg	NA
rs3473143	G	T	-0.06266	0.01373	7.71E-06	15829	genus.Coprococcus3.id.11303.s	TRUE	reported	IPHJjg	NA
rs1107735	C	T	-0.06454	0.014883	9.64E-06	15378	genus.Coprococcus3.id.11303.s	TRUE	reported	IPHJjg	NA
rs6994742	T	C	0.055131	0.012393	7.84E-06	15834	genus.Coprococcus3.id.11303.s	TRUE	reported	IPHJjg	NA
rs1324735	A	G	0.051211	0.011303	7.33E-06	15833	genus.Coprococcus3.id.11303.s	TRUE	reported	IPHJjg	NA
rs4575475	A	G	0.061951	0.013779	7.04E-06	15154	genus.Coprococcus3.id.11303.s	TRUE	reported	IPHJjg	NA
rs1081004	G	A	0.051552	0.011592	9.27E-06	15833	genus.Coprococcus3.id.11303.s	TRUE	reported	IPHJjg	NA
rs4677103	G	A	0.097791	0.019726	9.60E-07	7983	genus.DefluviitaleaceaeUCG011	TRUE	reported	IMnq1j	NA
rs7273181	T	C	-0.14738	0.02938	4.33E-07	7767	genus.DefluviitaleaceaeUCG011	TRUE	reported	IMnq1j	NA
rs1128938	C	T	0.11381	0.023281	1.45E-06	8183	genus.DefluviitaleaceaeUCG011	TRUE	reported	IMnq1j	NA
rs9725395	G	A	-0.13834	0.029554	3.52E-06	8011	genus.DefluviitaleaceaeUCG011	TRUE	reported	IMnq1j	NA
rs5565861	C	T	0.174369	0.036223	2.15E-06	6663	genus.DefluviitaleaceaeUCG011	TRUE	reported	IMnq1j	NA
rs9608282	G	T	0.142939	0.029979	2.52E-06	7716	genus.DefluviitaleaceaeUCG011	TRUE	reported	IMnq1j	NA
rs2869612	T	A	-0.1065	0.023847	6.63E-06	7811	genus.DefluviitaleaceaeUCG011	TRUE	reported	IMnq1j	NA
rs1582238	C	T	0.080504	0.016726	1.57E-06	7983	genus.DefluviitaleaceaeUCG011	TRUE	reported	IMnq1j	NA
rs1212299	T	G	-0.08273	0.018115	4.83E-06	7811	genus.DefluviitaleaceaeUCG011	TRUE	reported	IMnq1j	NA

rs4344384	T	G	0.071598	0.015631	4.83E-06	8180	genus.DefluviitaleaceaeUCG011	TRUE	reported	IMnq1j	NA
rs2892880	A	G	0.081776	0.018169	6.83E-06	8182	genus.DefluviitaleaceaeUCG011	TRUE	reported	IMnq1j	NA
rs1116035	A	T	-0.06918	0.014729	2.70E-06	9234	genus.Desulfovibrio.id.3173.s	TRUE	reported	4HeSAm	NA
rs2853179	T	C	0.081197	0.017423	2.42E-06	8949	genus.Desulfovibrio.id.3173.s	TRUE	reported	4HeSAm	NA
rs1686336	G	A	0.109402	0.022696	1.79E-06	8776	genus.Desulfovibrio.id.3173.s	TRUE	reported	4HeSAm	NA
rs1203154	C	T	-0.12718	0.028189	6.55E-06	8855	genus.Desulfovibrio.id.3173.s	TRUE	reported	4HeSAm	NA
rs1306614	A	G	0.119141	0.025089	3.79E-06	9241	genus.Desulfovibrio.id.3173.s	TRUE	reported	4HeSAm	NA
rs1294544	C	A	0.068878	0.015363	7.78E-06	9241	genus.Desulfovibrio.id.3173.s	TRUE	reported	4HeSAm	NA
rs2590913	A	G	0.154496	0.03394	6.65E-06	7225	genus.Desulfovibrio.id.3173.s	TRUE	reported	4HeSAm	NA
rs2032031	G	A	-0.06549	0.014868	9.14E-06	9241	genus.Desulfovibrio.id.3173.s	TRUE	reported	4HeSAm	NA
rs7264708	G	T	-0.10659	0.023934	8.30E-06	8985	genus.Desulfovibrio.id.3173.s	TRUE	reported	4HeSAm	NA
rs7729080	A	C	-0.07029	0.015769	9.96E-06	9241	genus.Desulfovibrio.id.3173.s	TRUE	reported	4HeSAm	NA
rs4797774	A	G	0.212582	0.047004	5.64E-06	3476	genus.Desulfovibrio.id.3173.s	TRUE	reported	4HeSAm	NA
rs6580353	C	T	0.077098	0.016974	4.94E-06	9241	genus.Desulfovibrio.id.3173.s	TRUE	reported	4HeSAm	NA
rs1116670	A	G	-0.06553	0.013186	5.51E-07	11720	genus.Dialister.id.2183.summa	TRUE	reported	DxKRlf	NA
rs517089	A	T	0.076227	0.017041	5.14E-06	11988	genus.Dialister.id.2183.summa	TRUE	reported	DxKRlf	NA
rs4747450	A	C	0.066851	0.014769	5.84E-06	11987	genus.Dialister.id.2183.summa	TRUE	reported	DxKRlf	NA
rs2314294	C	T	0.086593	0.019372	8.08E-06	11339	genus.Dialister.id.2183.summa	TRUE	reported	DxKRlf	NA
rs4753063	A	G	-0.05963	0.013005	4.86E-06	11989	genus.Dialister.id.2183.summa	TRUE	reported	DxKRlf	NA
rs2435610	C	A	0.064713	0.014331	5.93E-06	11984	genus.Dialister.id.2183.summa	TRUE	reported	DxKRlf	NA
rs1093893	A	G	-0.07735	0.017091	7.37E-06	11401	genus.Dialister.id.2183.summa	TRUE	reported	DxKRlf	NA
rs1013845	C	T	-0.11305	0.026193	7.88E-06	10968	genus.Dialister.id.2183.summa	TRUE	reported	DxKRlf	NA
rs1107188	C	T	0.06624	0.014631	5.91E-06	11989	genus.Dialister.id.2183.summa	TRUE	reported	DxKRlf	NA
rs764177	A	C	-0.06015	0.013537	9.61E-06	11988	genus.Dialister.id.2183.summa	TRUE	reported	DxKRlf	NA
rs7668046	A	G	-0.1613	0.036433	8.19E-06	7176	genus.Dialister.id.2183.summa	TRUE	reported	DxKRlf	NA
rs7541697	G	A	0.072722	0.016451	9.46E-06	11402	genus.Dialister.id.2183.summa	TRUE	reported	DxKRlf	NA
rs1327914	A	G	0.071531	0.015088	2.25E-06	17605	genus.Dorea.id.1997.summary.t	TRUE	reported	cbnjd5	NA
rs6250316	G	A	-0.09741	0.019438	7.47E-07	16210	genus.Dorea.id.1997.summary.t	TRUE	reported	cbnjd5	NA
rs7877251	C	A	0.13826	0.028361	3.38E-06	10858	genus.Dorea.id.1997.summary.t	TRUE	reported	cbnjd5	NA
rs1221616	A	T	0.088172	0.019368	5.33E-06	16086	genus.Dorea.id.1997.summary.t	TRUE	reported	cbnjd5	NA
rs7372943	T	C	-0.13745	0.029998	3.17E-06	9596	genus.Dorea.id.1997.summary.t	TRUE	reported	cbnjd5	NA
rs4793307	T	C	0.057417	0.012247	4.01E-06	17600	genus.Dorea.id.1997.summary.t	TRUE	reported	cbnjd5	NA
rs1115040	G	T	0.048825	0.010906	7.06E-06	17215	genus.Dorea.id.1997.summary.t	TRUE	reported	cbnjd5	NA

rs345219	G	T	-0.04974	0.011262	8.80E-06	17215	genus.Dorea.id.1997.summary.t	TRUE	reported	cbnjd5	NA
rs6258346	A	T	-0.06341	0.014223	5.78E-06	16819	genus.Dorea.id.1997.summary.t	TRUE	reported	cbnjd5	NA
rs3005511	G	A	0.051613	0.011285	5.29E-06	17610	genus.Dorea.id.1997.summary.t	TRUE	reported	cbnjd5	NA
rs1899291	T	C	0.069722	0.015028	4.57E-06	17609	genus.Dorea.id.1997.summary.t	TRUE	reported	cbnjd5	NA
rs3752849	A	G	0.163846	0.036608	7.68E-06	5322	genus.Dorea.id.1997.summary.t	TRUE	reported	cbnjd5	NA
rs1253778	C	T	-0.05554	0.012522	9.15E-06	17608	genus.Dorea.id.1997.summary.t	TRUE	reported	cbnjd5	NA
rs3851328	G	T	-0.10777	0.023653	4.18E-06	5147	genus.Eggerthella.id.819.summ	TRUE	reported	BEkAYV	NA
rs2240838	G	A	0.098058	0.019765	7.36E-07	5147	genus.Eggerthella.id.819.summ	TRUE	reported	BEkAYV	NA
rs2223081	A	G	0.102572	0.022111	3.89E-06	5147	genus.Eggerthella.id.819.summ	TRUE	reported	BEkAYV	NA
rs1122052	C	T	-0.18862	0.040361	3.35E-06	5036	genus.Eggerthella.id.819.summ	TRUE	reported	BEkAYV	NA
rs4985746	A	G	0.110522	0.024765	5.71E-06	4906	genus.Eggerthella.id.819.summ	TRUE	reported	BEkAYV	NA
rs1784446	A	G	0.090773	0.01981	5.23E-06	5147	genus.Eggerthella.id.819.summ	TRUE	reported	BEkAYV	NA
rs7666350	T	C	0.175349	0.037858	4.83E-06	4814	genus.Eggerthella.id.819.summ	TRUE	reported	BEkAYV	NA
rs6430926	T	C	0.087974	0.019713	8.37E-06	5147	genus.Eggerthella.id.819.summ	TRUE	reported	BEkAYV	NA
rs2877457	A	G	-0.09349	0.021021	9.03E-06	5083	genus.Eggerthella.id.819.summ	TRUE	reported	BEkAYV	NA
rs1307073	C	A	-0.12133	0.027202	7.62E-06	5147	genus.Eggerthella.id.819.summ	TRUE	reported	BEkAYV	NA
rs6749056	C	T	0.108495	0.024546	8.94E-06	5083	genus.Eggerthella.id.819.summ	TRUE	reported	BEkAYV	NA
rs3812426	A	G	0.106447	0.022416	2.72E-06	5583	genus.Eisenbergiella.id.11304	TRUE	reported	9oGX4V	NA
rs1508033	C	A	0.091546	0.019579	3.23E-06	5580	genus.Eisenbergiella.id.11304	TRUE	reported	9oGX4V	NA
rs1227856	A	T	-0.12106	0.025166	1.65E-06	5582	genus.Eisenbergiella.id.11304	TRUE	reported	9oGX4V	NA
rs2683098	T	C	0.107319	0.022515	2.24E-06	5582	genus.Eisenbergiella.id.11304	TRUE	reported	9oGX4V	NA
rs1107915	C	T	0.100627	0.022546	7.35E-06	5582	genus.Eisenbergiella.id.11304	TRUE	reported	9oGX4V	NA
rs1325885	G	A	0.137001	0.030212	7.75E-06	5583	genus.Eisenbergiella.id.11304	TRUE	reported	9oGX4V	NA
rs1271072	A	C	0.089338	0.019907	9.84E-06	5583	genus.Eisenbergiella.id.11304	TRUE	reported	9oGX4V	NA
rs1225772	C	A	-0.09527	0.021157	8.85E-06	5582	genus.Eisenbergiella.id.11304	TRUE	reported	9oGX4V	NA
rs4462860	A	G	0.093907	0.02011	4.16E-06	5460	genus.Eisenbergiella.id.11304	TRUE	reported	9oGX4V	NA
rs1102764	T	C	0.129006	0.028484	4.92E-06	5583	genus.Eisenbergiella.id.11304	TRUE	reported	9oGX4V	NA
rs1193860	C	T	0.097813	0.021664	8.22E-06	5583	genus.Eisenbergiella.id.11304	TRUE	reported	9oGX4V	NA
rs1553971	G	T	0.120965	0.026305	5.27E-06	5583	genus.Eisenbergiella.id.11304	TRUE	reported	9oGX4V	NA
rs1109886	A	T	-0.0966	0.016268	3.06E-09	7524	genus.Enterorhabdus.id.820.su	TRUE	reported	9jAB0B	NA
rs7923280	T	A	0.086002	0.016915	5.24E-07	7524	genus.Enterorhabdus.id.820.su	TRUE	reported	9jAB0B	NA
rs1147317	G	T	0.182308	0.03823	2.17E-06	5956	genus.Enterorhabdus.id.820.su	TRUE	reported	9jAB0B	NA
rs3017103	G	A	0.09809	0.020899	2.94E-06	7524	genus.Enterorhabdus.id.820.su	TRUE	reported	9jAB0B	NA

rs7333171	C	T	0.26199	0.055123	4.85E-06	3109	genus.Enterorhabdus.id.820.su	TRUE	reported	9jABOB	NA
rs7765528	A	G	0.132988	0.029831	5.88E-06	7494	genus.Enterorhabdus.id.820.su	TRUE	reported	9jABOB	NA
rs9470637	T	A	-0.07555	0.016626	5.70E-06	7524	genus.Enterorhabdus.id.820.su	TRUE	reported	9jABOB	NA
rs424715	C	T	0.081874	0.017485	4.41E-06	7524	genus.Enterorhabdus.id.820.su	TRUE	reported	9jABOB	NA
rs1009849	C	T	0.132323	0.029369	6.41E-06	7361	genus.Enterorhabdus.id.820.su	TRUE	reported	9jABOB	NA
rs2051957	T	C	0.084329	0.018986	8.90E-06	7524	genus.Enterorhabdus.id.820.su	TRUE	reported	9jABOB	NA
rs7221249	G	A	0.083986	0.014274	4.31E-09	9781	genus.Erysipelatoclostridium.	TRUE	reported	SL2Y7s	NA
rs710230	C	T	0.143381	0.028151	6.33E-07	9016	genus.Erysipelatoclostridium.	TRUE	reported	SL2Y7s	NA
rs4697572	G	A	-0.08106	0.016331	7.59E-07	9779	genus.Erysipelatoclostridium.	TRUE	reported	SL2Y7s	NA
rs5823656	T	G	-0.11116	0.023443	2.16E-06	9368	genus.Erysipelatoclostridium.	TRUE	reported	SL2Y7s	NA
rs622418	G	A	-0.06681	0.014322	3.68E-06	9783	genus.Erysipelatoclostridium.	TRUE	reported	SL2Y7s	NA
rs1780423	C	T	-0.06629	0.01442	4.59E-06	9783	genus.Erysipelatoclostridium.	TRUE	reported	SL2Y7s	NA
rs1434153	A	G	-0.06845	0.015244	6.85E-06	9662	genus.Erysipelatoclostridium.	TRUE	reported	SL2Y7s	NA
rs340991	G	A	-0.07405	0.015889	3.75E-06	9781	genus.Erysipelatoclostridium.	TRUE	reported	SL2Y7s	NA
rs6474512	C	A	0.067015	0.01432	3.02E-06	9783	genus.Erysipelatoclostridium.	TRUE	reported	SL2Y7s	NA
rs9590927	A	G	-0.0646	0.014348	6.39E-06	9782	genus.Erysipelatoclostridium.	TRUE	reported	SL2Y7s	NA
rs6180697	T	C	0.142548	0.032069	9.09E-06	8644	genus.Erysipelatoclostridium.	TRUE	reported	SL2Y7s	NA
rs6206036	C	T	-0.0798	0.018136	6.70E-06	9662	genus.Erysipelatoclostridium.	TRUE	reported	SL2Y7s	NA
rs4548039	G	T	-0.06871	0.015184	7.66E-06	9604	genus.Erysipelatoclostridium.	TRUE	reported	SL2Y7s	NA
rs2901723	A	C	0.064134	0.014429	8.79E-06	9662	genus.Erysipelatoclostridium.	TRUE	reported	SL2Y7s	NA
rs3452814	G	C	-0.08756	0.019556	6.13E-06	9604	genus.Erysipelatoclostridium.	TRUE	reported	SL2Y7s	NA
rs1693667	T	C	-0.0968	0.021788	6.04E-06	9488	genus.Erysipelatoclostridium.	TRUE	reported	SL2Y7s	NA
rs3804326	G	A	0.14147	0.033635	9.85E-06	7028	genus.Erysipelatoclostridium.	TRUE	reported	SL2Y7s	NA
rs2856839	G	A	-0.05839	0.011874	6.42E-07	14544	genus.ErysipelotrichaceaeUCG0	TRUE	reported	hFr16d	NA
rs1199430	T	C	0.115417	0.02425	1.33E-06	13558	genus.ErysipelotrichaceaeUCG0	TRUE	reported	hFr16d	NA
rs7650220	C	T	0.144844	0.028993	6.41E-07	10740	genus.ErysipelotrichaceaeUCG0	TRUE	reported	hFr16d	NA
rs2302840	C	T	-0.0872	0.017687	1.29E-06	14897	genus.ErysipelotrichaceaeUCG0	TRUE	reported	hFr16d	NA
rs1016406	G	T	-0.10326	0.021241	1.13E-06	14427	genus.ErysipelotrichaceaeUCG0	TRUE	reported	hFr16d	NA
rs8053479	G	A	-0.08382	0.018651	5.83E-06	13813	genus.ErysipelotrichaceaeUCG0	TRUE	reported	hFr16d	NA
rs1779813	A	G	0.158818	0.034767	3.24E-06	7421	genus.ErysipelotrichaceaeUCG0	TRUE	reported	hFr16d	NA
rs1166612	G	A	-0.07192	0.016137	7.90E-06	13813	genus.ErysipelotrichaceaeUCG0	TRUE	reported	hFr16d	NA
rs6240346	C	T	-0.07317	0.015652	3.44E-06	14899	genus.ErysipelotrichaceaeUCG0	TRUE	reported	hFr16d	NA
rs5906808	G	T	0.056477	0.012022	3.12E-06	14544	genus.ErysipelotrichaceaeUCG0	TRUE	reported	hFr16d	NA

rs7307443	T	C	0.072154	0.016438	9.99E-06	14217	genus.ErysipelotrichaceaeUCG0	TRUE	reported	hFr16d	NA
rs214586	C	T	-0.10645	0.024038	8.50E-06	13505	genus.ErysipelotrichaceaeUCG0	TRUE	reported	hFr16d	NA
rs4758231	T	G	-0.05518	0.012205	6.55E-06	14890	genus.ErysipelotrichaceaeUCG0	TRUE	reported	hFr16d	NA
rs7498898	A	G	-0.13314	0.034857	8.64E-06	5944	genus.ErysipelotrichaceaeUCG0	TRUE	reported	hFr16d	NA
rs7594902	C	T	-0.16967	0.037424	3.58E-06	6721	genus.ErysipelotrichaceaeUCG0	TRUE	reported	hFr16d	NA
rs6875357	T	C	0.165673	0.035383	6.70E-06	7034	genus.ErysipelotrichaceaeUCG0	TRUE	reported	hFr16d	NA
rs7939653	G	C	0.084815	0.019115	8.63E-06	13750	genus.ErysipelotrichaceaeUCG0	TRUE	reported	hFr16d	NA
rs1225139	G	A	-0.07054	0.015877	9.52E-06	14211	genus.ErysipelotrichaceaeUCG0	TRUE	reported	hFr16d	NA
rs2267739	C	G	0.115505	0.023763	1.42E-06	11853	genus.Escherichia.Shigella.id	TRUE	reported	70JHDT	NA
rs1154904	G	A	-0.06134	0.013064	3.04E-06	12002	genus.Escherichia.Shigella.id	TRUE	reported	70JHDT	NA
rs7320816	G	A	-0.1193	0.024839	2.19E-06	11073	genus.Escherichia.Shigella.id	TRUE	reported	70JHDT	NA
rs1135138	G	A	0.172283	0.038041	5.28E-06	6280	genus.Escherichia.Shigella.id	TRUE	reported	70JHDT	NA
rs7502686	C	G	-0.13638	0.030258	5.90E-06	9990	genus.Escherichia.Shigella.id	TRUE	reported	70JHDT	NA
rs5702427	C	T	0.062617	0.013992	9.70E-06	11853	genus.Escherichia.Shigella.id	TRUE	reported	70JHDT	NA
rs1131270	G	A	0.151009	0.032343	3.33E-06	8433	genus.Escherichia.Shigella.id	TRUE	reported	70JHDT	NA
rs1170923	T	A	0.117362	0.026453	9.65E-06	10304	genus.Escherichia.Shigella.id	TRUE	reported	70JHDT	NA
rs1127672	C	T	0.073296	0.016359	8.21E-06	11771	genus.Escherichia.Shigella.id	TRUE	reported	70JHDT	NA
rs1170604	A	T	0.075657	0.016416	5.87E-06	12001	genus.Escherichia.Shigella.id	TRUE	reported	70JHDT	NA
rs592299	C	T	-0.0592	0.012949	4.77E-06	11995	genus.Escherichia.Shigella.id	TRUE	reported	70JHDT	NA
rs4731451	A	G	-0.06099	0.013516	7.47E-06	12001	genus.Escherichia.Shigella.id	TRUE	reported	70JHDT	NA
rs3555551	G	C	0.101976	0.022288	4.92E-06	12002	genus.Escherichia.Shigella.id	TRUE	reported	70JHDT	NA
rs118526	A	C	-0.05944	0.013598	8.00E-06	11853	genus.Escherichia.Shigella.id	TRUE	reported	70JHDT	NA
rs2798105	G	A	-0.10085	0.022204	8.25E-06	11296	genus.Escherichia.Shigella.id	TRUE	reported	70JHDT	NA
rs1232084	G	C	0.094833	0.016396	7.57E-09	17638	genus.Faecalibacterium.id.205	TRUE	reported	lbF15z	NA
rs6910935	G	A	0.134864	0.027703	1.38E-06	12640	genus.Faecalibacterium.id.205	TRUE	reported	lbF15z	NA
rs1271565	T	C	-0.05763	0.011965	1.30E-06	17638	genus.Faecalibacterium.id.205	TRUE	reported	lbF15z	NA
rs7549906	T	C	0.227585	0.046552	1.76E-06	4526	genus.Faecalibacterium.id.205	TRUE	reported	lbF15z	NA
rs9536330	C	T	-0.04832	0.010797	5.33E-06	18082	genus.Faecalibacterium.id.205	TRUE	reported	lbF15z	NA
rs2837666	G	C	0.050482	0.010937	3.66E-06	18087	genus.Faecalibacterium.id.205	TRUE	reported	lbF15z	NA
rs1092739	T	G	-0.23226	0.051236	7.02E-06	3495	genus.Faecalibacterium.id.205	TRUE	reported	lbF15z	NA
rs1149469	T	C	-0.08616	0.01896	5.70E-06	15673	genus.Faecalibacterium.id.205	TRUE	reported	lbF15z	NA
rs7965663	C	T	0.145631	0.032302	8.14E-06	9080	genus.Faecalibacterium.id.205	TRUE	reported	lbF15z	NA
rs6187548	G	C	0.081795	0.018369	9.18E-06	16718	genus.Faecalibacterium.id.205	TRUE	reported	lbF15z	NA

rs1275349	C	A	0.06413	0.014993	8.80E-06	17525	genus.Faecalibacterium.id.205	TRUE	reported	lbF15z	NA
rs2835874	C	T	-0.08664	0.01965	7.54E-06	17567	genus.Faecalibacterium.id.205	TRUE	reported	lbF15z	NA
rs1177639	C	T	-0.07835	0.017183	6.40E-06	16718	genus.Faecalibacterium.id.205	TRUE	reported	lbF15z	NA
rs9852893	G	C	0.065783	0.012906	3.88E-07	14184	genus.FamilyXIIIAD3011group.i	TRUE	reported	jB9coN	NA
rs6220041	T	C	-0.08009	0.016383	5.80E-07	13661	genus.FamilyXIIIAD3011group.i	TRUE	reported	jB9coN	NA
rs1684031	G	A	-0.06081	0.012215	6.75E-07	14201	genus.FamilyXIIIAD3011group.i	TRUE	reported	jB9coN	NA
rs7273093	A	C	-0.08996	0.017711	6.89E-07	13102	genus.FamilyXIIIAD3011group.i	TRUE	reported	jB9coN	NA
rs1694016	T	C	0.073255	0.01599	3.91E-06	14201	genus.FamilyXIIIAD3011group.i	TRUE	reported	jB9coN	NA
rs1715684	A	G	-0.11289	0.024529	4.19E-06	13352	genus.FamilyXIIIAD3011group.i	TRUE	reported	jB9coN	NA
rs6202976	G	A	0.128753	0.0276	3.89E-06	9655	genus.FamilyXIIIAD3011group.i	TRUE	reported	jB9coN	NA
rs1173661	A	G	-0.07595	0.01721	9.02E-06	13778	genus.FamilyXIIIAD3011group.i	TRUE	reported	jB9coN	NA
rs1281267	C	T	-0.09607	0.020828	2.56E-06	13565	genus.FamilyXIIIAD3011group.i	TRUE	reported	jB9coN	NA
rs9276029	G	A	-0.08114	0.018567	8.93E-06	13566	genus.FamilyXIIIAD3011group.i	TRUE	reported	jB9coN	NA
rs1291184	T	A	-0.0812	0.018334	6.91E-06	14125	genus.FamilyXIIIAD3011group.i	TRUE	reported	jB9coN	NA
rs149302	C	T	-0.06456	0.014322	7.48E-06	14192	genus.FamilyXIIIAD3011group.i	TRUE	reported	jB9coN	NA
rs9837139	G	A	0.10752	0.024048	8.71E-06	13527	genus.FamilyXIIIAD3011group.i	TRUE	reported	jB9coN	NA
rs739451	T	C	0.064959	0.014753	7.88E-06	14197	genus.FamilyXIIIAD3011group.i	TRUE	reported	jB9coN	NA
rs1112642	T	C	0.09044	0.01963	5.91E-06	13735	genus.FamilyXIIIAD3011group.i	TRUE	reported	jB9coN	NA
rs1426266	C	T	-0.06655	0.013715	1.25E-06	12751	genus.FamilyXIIIUCG001.id.112	TRUE	reported	1ElmdG	NA
rs1204945	C	T	-0.06473	0.013407	1.17E-06	12748	genus.FamilyXIIIUCG001.id.112	TRUE	reported	1ElmdG	NA
rs3842897	A	G	-0.11264	0.024275	5.20E-06	12407	genus.FamilyXIIIUCG001.id.112	TRUE	reported	1ElmdG	NA
rs1169795	A	T	-0.12168	0.026082	3.05E-06	11989	genus.FamilyXIIIUCG001.id.112	TRUE	reported	1ElmdG	NA
rs6241480	T	C	-0.06119	0.013457	4.29E-06	12750	genus.FamilyXIIIUCG001.id.112	TRUE	reported	1ElmdG	NA
rs2276529	G	C	-0.07594	0.01651	5.36E-06	12748	genus.FamilyXIIIUCG001.id.112	TRUE	reported	1ElmdG	NA
rs1123629	G	A	-0.14905	0.033327	7.88E-06	8579	genus.FamilyXIIIUCG001.id.112	TRUE	reported	1ElmdG	NA
rs8076666	G	A	0.088655	0.019809	8.02E-06	12265	genus.FamilyXIIIUCG001.id.112	TRUE	reported	1ElmdG	NA
rs7119679	A	G	-0.08091	0.017478	3.52E-06	12445	genus.FamilyXIIIUCG001.id.112	TRUE	reported	1ElmdG	NA
rs7646377	G	A	0.19313	0.041987	3.77E-06	5651	genus.FamilyXIIIUCG001.id.112	TRUE	reported	1ElmdG	NA
rs1203030	G	A	-0.06923	0.013747	5.61E-07	10805	genus.Flavonifractor.id.2059.	TRUE	reported	0jpvOB	NA
rs806808	C	T	0.066727	0.013656	1.18E-06	10801	genus.Flavonifractor.id.2059.	TRUE	reported	0jpvOB	NA
rs3406601	G	A	0.076429	0.015979	1.52E-06	10705	genus.Flavonifractor.id.2059.	TRUE	reported	0jpvOB	NA
rs1181169	C	T	-0.11606	0.024101	2.07E-06	10221	genus.Flavonifractor.id.2059.	TRUE	reported	0jpvOB	NA
rs4949766	C	T	-0.06875	0.015166	4.83E-06	10805	genus.Flavonifractor.id.2059.	TRUE	reported	0jpvOB	NA

rs1164282	C	G	0.14686	0.032519	6.65E-06	8151	genus.Flavonifractor.id.2059.	TRUE	reported	0jpv0B	NA
rs1148735	T	C	-0.13007	0.029409	7.13E-06	9156	genus.Flavonifractor.id.2059.	TRUE	reported	0jpv0B	NA
rs6761463	C	G	-0.08339	0.01846	8.11E-06	10805	genus.Flavonifractor.id.2059.	TRUE	reported	0jpv0B	NA
rs798674	C	G	0.063713	0.014161	7.02E-06	10583	genus.Flavonifractor.id.2059.	TRUE	reported	0jpv0B	NA
rs1203888	G	C	0.094272	0.021165	9.37E-06	10583	genus.Flavonifractor.id.2059.	TRUE	reported	0jpv0B	NA
rs4378146	C	A	-0.06167	0.012526	7.20E-07	17384	genus.Fusicatenibacter.id.113	TRUE	reported	1PFG91	NA
rs6235348	G	A	-0.07014	0.014559	1.57E-06	16119	genus.Fusicatenibacter.id.113	TRUE	reported	1PFG91	NA
rs704418	C	T	0.073912	0.015107	7.77E-07	16626	genus.Fusicatenibacter.id.113	TRUE	reported	1PFG91	NA
rs2132128	A	G	-0.07719	0.016034	1.08E-06	17370	genus.Fusicatenibacter.id.113	TRUE	reported	1PFG91	NA
rs206581	G	A	-0.05683	0.012789	8.96E-06	17384	genus.Fusicatenibacter.id.113	TRUE	reported	1PFG91	NA
rs6218763	C	T	-0.07106	0.015924	4.55E-06	16522	genus.Fusicatenibacter.id.113	TRUE	reported	1PFG91	NA
rs2025938	A	G	-0.09673	0.020543	2.99E-06	15626	genus.Fusicatenibacter.id.113	TRUE	reported	1PFG91	NA
rs7069626	C	T	0.050901	0.011138	5.42E-06	17384	genus.Fusicatenibacter.id.113	TRUE	reported	1PFG91	NA
rs3303	C	T	-0.09537	0.020407	3.94E-06	16877	genus.Fusicatenibacter.id.113	TRUE	reported	1PFG91	NA
rs2039204	A	T	-0.04974	0.010794	3.94E-06	17383	genus.Fusicatenibacter.id.113	TRUE	reported	1PFG91	NA
rs8028026	G	A	-0.07921	0.01805	8.06E-06	16522	genus.Fusicatenibacter.id.113	TRUE	reported	1PFG91	NA
rs1864685	C	A	-0.04948	0.01081	4.96E-06	17374	genus.Fusicatenibacter.id.113	TRUE	reported	1PFG91	NA
rs792108	C	T	-0.05083	0.011384	8.50E-06	16978	genus.Fusicatenibacter.id.113	TRUE	reported	1PFG91	NA
rs6025419	G	A	-0.04924	0.010936	5.47E-06	16978	genus.Fusicatenibacter.id.113	TRUE	reported	1PFG91	NA
rs9905659	A	G	-0.06162	0.013658	7.31E-06	17384	genus.Fusicatenibacter.id.113	TRUE	reported	1PFG91	NA
rs8063430	C	T	-0.10403	0.022217	4.93E-06	15552	genus.Fusicatenibacter.id.113	TRUE	reported	1PFG91	NA
rs6515626	A	G	0.141575	0.031356	7.29E-06	7796	genus.Fusicatenibacter.id.113	TRUE	reported	1PFG91	NA
rs1043967	G	A	-0.05721	0.013001	7.68E-06	17381	genus.Fusicatenibacter.id.113	TRUE	reported	1PFG91	NA
rs167879	T	C	-0.06595	0.014876	5.87E-06	16542	genus.Fusicatenibacter.id.113	TRUE	reported	1PFG91	NA
rs7310391	G	A	-0.05973	0.013446	8.30E-06	17384	genus.Fusicatenibacter.id.113	TRUE	reported	1PFG91	NA
rs7628711	T	A	-0.24296	0.046618	1.67E-07	3483	genus.Gordonibacter.id.821.su	TRUE	reported	rSbgJh	NA
rs7294633	T	C	0.12867	0.024995	3.44E-07	3723	genus.Gordonibacter.id.821.su	TRUE	reported	rSbgJh	NA
rs7220558	T	A	0.116884	0.023483	6.71E-07	3723	genus.Gordonibacter.id.821.su	TRUE	reported	rSbgJh	NA
rs7154597	G	A	-0.15397	0.033892	7.04E-06	3556	genus.Gordonibacter.id.821.su	TRUE	reported	rSbgJh	NA
rs3504226	A	C	-0.18029	0.04033	8.11E-06	3595	genus.Gordonibacter.id.821.su	TRUE	reported	rSbgJh	NA
rs7271478	A	C	0.181405	0.03771	1.43E-06	3723	genus.Gordonibacter.id.821.su	TRUE	reported	rSbgJh	NA
rs322296	A	G	0.17869	0.037722	4.02E-06	3595	genus.Gordonibacter.id.821.su	TRUE	reported	rSbgJh	NA
rs7293951	G	A	-0.21399	0.049059	7.98E-06	3435	genus.Gordonibacter.id.821.su	TRUE	reported	rSbgJh	NA

rs3765837	G	T	-0.19073	0.043361	7.17E-06	3556	genus.Gordonibacter.id.821.su	TRUE	reported	rSbgJh	NA
rs1341265	C	A	0.107597	0.023923	8.61E-06	3723	genus.Gordonibacter.id.821.su	TRUE	reported	rSbgJh	NA
rs1173470	C	G	-0.12826	0.028512	9.17E-06	3684	genus.Gordonibacter.id.821.su	TRUE	reported	rSbgJh	NA
rs1695529	A	G	-0.19642	0.043354	6.37E-06	3572	genus.Gordonibacter.id.821.su	TRUE	reported	rSbgJh	NA
rs4596722	G	A	0.102908	0.023157	9.06E-06	3723	genus.Gordonibacter.id.821.su	TRUE	reported	rSbgJh	NA
rs768830	A	G	0.149852	0.033331	7.76E-06	3723	genus.Gordonibacter.id.821.su	TRUE	reported	rSbgJh	NA
rs6193459	T	C	-0.17249	0.038857	8.37E-06	3684	genus.Gordonibacter.id.821.su	TRUE	reported	rSbgJh	NA
rs1219168	G	C	0.106544	0.020032	1.47E-07	9115	genus.Haemophilus.id.3698.sum	TRUE	reported	QjhWut	NA
rs9382510	T	C	-0.09352	0.017265	7.12E-08	9117	genus.Haemophilus.id.3698.sum	TRUE	reported	QjhWut	NA
rs9574096	T	A	-0.07364	0.015523	2.18E-06	9118	genus.Haemophilus.id.3698.sum	TRUE	reported	QjhWut	NA
rs7602235	T	C	0.244638	0.05055	1.83E-06	3618	genus.Haemophilus.id.3698.sum	TRUE	reported	QjhWut	NA
rs1115828	A	G	-0.12427	0.026016	1.27E-06	8370	genus.Haemophilus.id.3698.sum	TRUE	reported	QjhWut	NA
rs9895850	C	T	-0.19296	0.041676	2.14E-06	4592	genus.Haemophilus.id.3698.sum	TRUE	reported	QjhWut	NA
rs35509	A	G	0.128249	0.026878	2.01E-06	8430	genus.Haemophilus.id.3698.sum	TRUE	reported	QjhWut	NA
rs7890900	C	T	-0.24626	0.050392	1.67E-06	3559	genus.Haemophilus.id.3698.sum	TRUE	reported	QjhWut	NA
rs9328464	C	T	0.072309	0.01488	1.42E-06	9119	genus.Haemophilus.id.3698.sum	TRUE	reported	QjhWut	NA
rs4822728	C	T	0.070586	0.015139	3.48E-06	9119	genus.Haemophilus.id.3698.sum	TRUE	reported	QjhWut	NA
rs1084032	G	C	-0.06774	0.015146	7.37E-06	8888	genus.Haemophilus.id.3698.sum	TRUE	reported	QjhWut	NA
rs1287618	A	T	0.07492	0.016711	9.62E-06	9110	genus.Haemophilus.id.3698.sum	TRUE	reported	QjhWut	NA
rs1078134	A	G	0.094892	0.020322	4.32E-06	9037	genus.Haemophilus.id.3698.sum	TRUE	reported	QjhWut	NA
rs5631094	C	G	-0.10839	0.024705	7.23E-06	9119	genus.Haemophilus.id.3698.sum	TRUE	reported	QjhWut	NA
rs761624	G	C	0.09598	0.017954	1.38E-07	7706	genus.Holdemanella.id.11393.s	TRUE	reported	UbTUNR	NA
rs607782	C	T	-0.08542	0.017252	7.19E-07	7456	genus.Holdemanella.id.11393.s	TRUE	reported	UbTUNR	NA
rs7576468	C	T	-0.2831	0.0599	1.94E-06	3003	genus.Holdemanella.id.11393.s	TRUE	reported	UbTUNR	NA
rs7301127	C	T	-0.09617	0.019934	1.36E-06	7456	genus.Holdemanella.id.11393.s	TRUE	reported	UbTUNR	NA
rs4541991	C	T	-0.09273	0.019443	2.10E-06	7657	genus.Holdemanella.id.11393.s	TRUE	reported	UbTUNR	NA
rs1830029	G	C	-0.09541	0.021093	5.35E-06	7456	genus.Holdemanella.id.11393.s	TRUE	reported	UbTUNR	NA
rs1251318	A	G	0.090391	0.019528	4.65E-06	7700	genus.Holdemanella.id.11393.s	TRUE	reported	UbTUNR	NA
rs1241564	C	G	0.083938	0.019057	7.88E-06	7662	genus.Holdemanella.id.11393.s	TRUE	reported	UbTUNR	NA
rs1926302	A	G	-0.10797	0.023142	7.50E-06	7702	genus.Holdemanella.id.11393.s	TRUE	reported	UbTUNR	NA
rs1758676	C	T	-0.22728	0.051013	7.72E-06	3817	genus.Holdemanella.id.11393.s	TRUE	reported	UbTUNR	NA
rs8113760	A	G	0.078999	0.01734	4.62E-06	7456	genus.Holdemanella.id.11393.s	TRUE	reported	UbTUNR	NA
rs3418711	A	C	-0.10451	0.022601	5.13E-06	7704	genus.Holdemanella.id.11393.s	TRUE	reported	UbTUNR	NA

rs3522829	A	G	0.093483	0.020289	7.30E-06	7456	genus.Holdemanella.id.11393.s	TRUE	reported	UbTUNR	NA
rs6211338	C	T	-0.10545	0.023203	5.54E-06	7310	genus.Holdemanella.id.11393.s	TRUE	reported	UbTUNR	NA
rs1867876	C	T	0.084292	0.016219	2.74E-07	9109	genus.Holdemania.id.2157.summ	TRUE	reported	kI70by	NA
rs9500080	T	C	0.092676	0.017889	4.09E-07	9106	genus.Holdemania.id.2157.summ	TRUE	reported	kI70by	NA
rs6133067	C	T	0.091083	0.017856	5.17E-07	8863	genus.Holdemania.id.2157.summ	TRUE	reported	kI70by	NA
rs1500961	A	T	0.162139	0.033209	2.38E-06	6906	genus.Holdemania.id.2157.summ	TRUE	reported	kI70by	NA
rs4143874	G	C	-0.12487	0.027013	2.44E-06	8839	genus.Holdemania.id.2157.summ	TRUE	reported	kI70by	NA
rs7729340	G	A	0.164556	0.034177	1.77E-06	7585	genus.Holdemania.id.2157.summ	TRUE	reported	kI70by	NA
rs8014966	T	C	-0.23299	0.051918	6.04E-06	3283	genus.Holdemania.id.2157.summ	TRUE	reported	kI70by	NA
rs1165009	T	G	-0.13755	0.029339	2.34E-06	8432	genus.Holdemania.id.2157.summ	TRUE	reported	kI70by	NA
rs5588818	G	C	0.128671	0.028279	5.89E-06	8464	genus.Holdemania.id.2157.summ	TRUE	reported	kI70by	NA
rs4146507	T	C	0.079488	0.0177	7.23E-06	9108	genus.Holdemania.id.2157.summ	TRUE	reported	kI70by	NA
rs1117459	G	A	0.120677	0.026578	3.71E-06	8233	genus.Holdemania.id.2157.summ	TRUE	reported	kI70by	NA
rs1135933	G	A	-0.12893	0.028253	9.36E-06	8296	genus.Holdemania.id.2157.summ	TRUE	reported	kI70by	NA
rs1088547	C	T	-0.13514	0.030189	8.60E-06	8690	genus.Holdemania.id.2157.summ	TRUE	reported	kI70by	NA
rs9529719	C	T	0.074038	0.016049	5.97E-06	9107	genus.Holdemania.id.2157.summ	TRUE	reported	kI70by	NA
rs967319	C	T	0.078864	0.017674	8.38E-06	9108	genus.Holdemania.id.2157.summ	TRUE	reported	kI70by	NA
rs1270161	G	A	-0.06606	0.014943	9.52E-06	9106	genus.Holdemania.id.2157.summ	TRUE	reported	kI70by	NA
rs7313953	A	G	-0.14859	0.032747	7.77E-06	8095	genus.Holdemania.id.2157.summ	TRUE	reported	kI70by	NA
rs1108006	A	G	-0.06652	0.014984	6.67E-06	9103	genus.Holdemania.id.2157.summ	TRUE	reported	kI70by	NA
rs901099	G	T	-0.12718	0.025092	6.53E-07	3647	genus.Howardella.id.2000.summ	TRUE	reported	IhD68d	NA
rs1484873	G	A	-0.22783	0.046335	2.56E-06	3608	genus.Howardella.id.2000.summ	TRUE	reported	IhD68d	NA
rs1716709	A	G	-0.16937	0.035207	1.12E-06	3586	genus.Howardella.id.2000.summ	TRUE	reported	IhD68d	NA
rs609430	G	T	-0.11205	0.023933	3.34E-06	3798	genus.Howardella.id.2000.summ	TRUE	reported	IhD68d	NA
rs3608191	C	T	-0.18123	0.040299	4.70E-06	3216	genus.Howardella.id.2000.summ	TRUE	reported	IhD68d	NA
rs1245294	G	A	-0.10583	0.022894	3.80E-06	3800	genus.Howardella.id.2000.summ	TRUE	reported	IhD68d	NA
rs6177180	T	A	-0.13678	0.029681	4.03E-06	3612	genus.Howardella.id.2000.summ	TRUE	reported	IhD68d	NA
rs672217	A	G	0.164146	0.034999	3.52E-06	3611	genus.Howardella.id.2000.summ	TRUE	reported	IhD68d	NA
rs3791893	G	A	0.147035	0.034023	9.50E-06	3795	genus.Howardella.id.2000.summ	TRUE	reported	IhD68d	NA
rs1004806	T	C	-0.14735	0.033653	8.59E-06	3800	genus.Howardella.id.2000.summ	TRUE	reported	IhD68d	NA
rs2154047	A	C	-0.19256	0.042009	9.97E-06	3623	genus.Howardella.id.2000.summ	TRUE	reported	IhD68d	NA
rs1312878	C	T	-0.14973	0.031278	1.75E-06	3999	genus.Hungatella.id.11306.sum	TRUE	reported	xYETYL	NA
rs7275904	T	G	-0.12603	0.028224	3.86E-06	3910	genus.Hungatella.id.11306.sum	TRUE	reported	xYETYL	NA

rs1004499	A	C	0.139547	0.031675	8.07E-06	4209	genus.Hungatella.id.11306.sum	TRUE	reported	xYETYL	NA
rs1324932	G	T	-0.10002	0.022588	9.69E-06	4209	genus.Hungatella.id.11306.sum	TRUE	reported	xYETYL	NA
rs1709261	A	G	0.152235	0.033787	7.38E-06	4009	genus.Hungatella.id.11306.sum	TRUE	reported	xYETYL	NA
rs1080532	A	G	0.077515	0.013966	3.55E-08	12303	genus.Intestinibacter.id.1134	TRUE	reported	nIcFw7	NA
rs4327025	A	G	-0.08103	0.01544	1.64E-07	12303	genus.Intestinibacter.id.1134	TRUE	reported	nIcFw7	NA
rs478972	C	T	-0.14268	0.029712	1.82E-06	10333	genus.Intestinibacter.id.1134	TRUE	reported	nIcFw7	NA
rs6875660	T	C	0.089046	0.019391	3.06E-06	12293	genus.Intestinibacter.id.1134	TRUE	reported	nIcFw7	NA
rs1693843	C	T	-0.11219	0.023544	1.80E-06	12303	genus.Intestinibacter.id.1134	TRUE	reported	nIcFw7	NA
rs6062862	G	A	0.092451	0.020466	6.68E-06	12189	genus.Intestinibacter.id.1134	TRUE	reported	nIcFw7	NA
rs1180302	A	G	-0.15183	0.032447	2.67E-06	8830	genus.Intestinibacter.id.1134	TRUE	reported	nIcFw7	NA
rs1110909	T	C	0.062427	0.013854	5.49E-06	11886	genus.Intestinibacter.id.1134	TRUE	reported	nIcFw7	NA
rs9348442	T	C	0.099082	0.022163	6.26E-06	12303	genus.Intestinibacter.id.1134	TRUE	reported	nIcFw7	NA
rs447950	G	A	0.062834	0.013665	5.64E-06	12303	genus.Intestinibacter.id.1134	TRUE	reported	nIcFw7	NA
rs6243035	C	T	0.151096	0.035148	6.84E-06	7650	genus.Intestinibacter.id.1134	TRUE	reported	nIcFw7	NA
rs2702387	G	A	0.060856	0.013215	4.26E-06	12291	genus.Intestinibacter.id.1134	TRUE	reported	nIcFw7	NA
rs6809321	T	C	0.066226	0.014988	9.26E-06	11886	genus.Intestinibacter.id.1134	TRUE	reported	nIcFw7	NA
rs2098844	T	C	-0.05753	0.012842	6.79E-06	12291	genus.Intestinibacter.id.1134	TRUE	reported	nIcFw7	NA
rs893394	A	G	0.058332	0.013073	7.85E-06	11886	genus.Intestinibacter.id.1134	TRUE	reported	nIcFw7	NA
rs1125817	G	A	0.066074	0.013414	6.98E-07	11766	genus.Intestinimonas.id.2062.	TRUE	reported	kf2010	NA
rs1222615	G	A	-0.15114	0.030692	5.12E-07	9023	genus.Intestinimonas.id.2062.	TRUE	reported	kf2010	NA
rs4784055	C	T	-0.17532	0.038599	8.72E-07	3193	genus.Intestinimonas.id.2062.	TRUE	reported	kf2010	NA
rs716604	G	A	0.081807	0.016599	8.57E-07	12090	genus.Intestinimonas.id.2062.	TRUE	reported	kf2010	NA
rs2930225	T	G	0.072952	0.015295	1.35E-06	12003	genus.Intestinimonas.id.2062.	TRUE	reported	kf2010	NA
rs6224018	A	G	0.130074	0.026718	2.20E-06	10252	genus.Intestinimonas.id.2062.	TRUE	reported	kf2010	NA
rs2731794	T	C	0.120632	0.025753	1.92E-06	11079	genus.Intestinimonas.id.2062.	TRUE	reported	kf2010	NA
rs7170984	C	T	-0.06581	0.014077	2.98E-06	12090	genus.Intestinimonas.id.2062.	TRUE	reported	kf2010	NA
rs1026270	C	T	0.091802	0.019488	2.06E-06	11766	genus.Intestinimonas.id.2062.	TRUE	reported	kf2010	NA
rs1256624	A	T	0.063708	0.013536	2.19E-06	12076	genus.Intestinimonas.id.2062.	TRUE	reported	kf2010	NA
rs1859797	A	G	0.060368	0.013179	4.12E-06	11766	genus.Intestinimonas.id.2062.	TRUE	reported	kf2010	NA
rs1192884	A	G	-0.06073	0.013204	4.21E-06	11766	genus.Intestinimonas.id.2062.	TRUE	reported	kf2010	NA
rs1706789	T	C	0.107194	0.025001	6.38E-06	11544	genus.Intestinimonas.id.2062.	TRUE	reported	kf2010	NA
rs7298291	T	C	0.183158	0.040274	4.91E-06	5591	genus.Intestinimonas.id.2062.	TRUE	reported	kf2010	NA
rs994794	C	G	-0.1419	0.031507	7.31E-06	9250	genus.Intestinimonas.id.2062.	TRUE	reported	kf2010	NA

rs41136760	C	A	-0.21851	0.049015	7.42E-06	3170	genus.Intestinimonas.id.2062.	TRUE	reported	kf2010	NA
rs62427233	A	C	0.162658	0.036765	9.41E-06	6385	genus.Intestinimonas.id.2062.	TRUE	reported	kf2010	NA
rs22767600	G	A	-0.06852	0.015255	7.84E-06	12081	genus.Intestinimonas.id.2062.	TRUE	reported	kf2010	NA
rs69345199	T	C	0.069237	0.015115	8.57E-06	12089	genus.Intestinimonas.id.2062.	TRUE	reported	kf2010	NA
rs10008888	G	C	-0.0585	0.013219	9.71E-06	12090	genus.Intestinimonas.id.2062.	TRUE	reported	kf2010	NA
rs61123144	C	A	-0.05617	0.010817	2.43E-07	17842	genus.Lachnoclostridium.id.11	TRUE	reported	L8BtIX	NA
rs62285311	G	A	0.08642	0.018157	1.58E-06	16991	genus.Lachnoclostridium.id.11	TRUE	reported	L8BtIX	NA
rs78068100	G	A	0.08862	0.019425	3.67E-06	17477	genus.Lachnoclostridium.id.11	TRUE	reported	L8BtIX	NA
rs615997	C	T	0.051175	0.010649	2.03E-06	17911	genus.Lachnoclostridium.id.11	TRUE	reported	L8BtIX	NA
rs61915999	T	A	0.080388	0.017221	2.67E-06	16773	genus.Lachnoclostridium.id.11	TRUE	reported	L8BtIX	NA
rs789029	T	C	-0.06413	0.013797	3.75E-06	17922	genus.Lachnoclostridium.id.11	TRUE	reported	L8BtIX	NA
rs12566977	C	T	-0.04681	0.010579	9.57E-06	17921	genus.Lachnoclostridium.id.11	TRUE	reported	L8BtIX	NA
rs47386799	A	G	-0.05203	0.011404	4.42E-06	17921	genus.Lachnoclostridium.id.11	TRUE	reported	L8BtIX	NA
rs10315999	T	G	-0.07863	0.017564	6.31E-06	17398	genus.Lachnoclostridium.id.11	TRUE	reported	L8BtIX	NA
rs72829899	T	G	0.117472	0.02681	5.58E-06	12404	genus.Lachnoclostridium.id.11	TRUE	reported	L8BtIX	NA
rs38219988	A	C	-0.08641	0.019252	6.72E-06	17920	genus.Lachnoclostridium.id.11	TRUE	reported	L8BtIX	NA
rs23854211	G	A	0.074619	0.018073	7.14E-06	17903	genus.Lachnoclostridium.id.11	TRUE	reported	L8BtIX	NA
rs15284799	A	G	-0.04978	0.011192	9.64E-06	17477	genus.Lachnoclostridium.id.11	TRUE	reported	L8BtIX	NA
rs19972044	C	T	-0.10807	0.024202	5.97E-06	14138	genus.Lachnoclostridium.id.11	TRUE	reported	L8BtIX	NA
rs62028344	C	G	0.046999	0.010597	9.17E-06	17922	genus.Lachnoclostridium.id.11	TRUE	reported	L8BtIX	NA
rs13157099	G	A	-0.07681	0.015531	5.99E-07	15947	genus.Lachnospira.id.2004.sum	TRUE	reported	9h73c6	NA
rs49233244	A	G	-0.06173	0.013339	2.44E-06	16490	genus.Lachnospira.id.2004.sum	TRUE	reported	9h73c6	NA
rs56791200	C	T	0.051823	0.01107	2.93E-06	16486	genus.Lachnospira.id.2004.sum	TRUE	reported	9h73c6	NA
rs23268333	G	C	-0.0785	0.017109	4.60E-06	15632	genus.Lachnospira.id.2004.sum	TRUE	reported	9h73c6	NA
rs46867988	C	T	0.053183	0.011375	2.74E-06	16497	genus.Lachnospira.id.2004.sum	TRUE	reported	9h73c6	NA
rs159484	A	G	0.079467	0.0177	6.68E-06	16487	genus.Lachnospira.id.2004.sum	TRUE	reported	9h73c6	NA
rs25205099	G	A	0.051906	0.011578	7.42E-06	16500	genus.Lachnospira.id.2004.sum	TRUE	reported	9h73c6	NA
rs72491133	A	G	0.067948	0.01335	3.72E-07	13534	genus.LachnospiraceaeFCS020gr	TRUE	reported	rSCYwx	NA
rs12078955	G	C	0.106101	0.02229	2.15E-06	13101	genus.LachnospiraceaeFCS020gr	TRUE	reported	rSCYwx	NA
rs369444	G	C	0.125481	0.025901	3.15E-06	12465	genus.LachnospiraceaeFCS020gr	TRUE	reported	rSCYwx	NA
rs72793666	G	A	-0.11688	0.024659	1.63E-06	12396	genus.LachnospiraceaeFCS020gr	TRUE	reported	rSCYwx	NA
rs13637699	C	T	-0.20063	0.044937	1.58E-06	3555	genus.LachnospiraceaeFCS020gr	TRUE	reported	rSCYwx	NA
rs10093866	A	G	-0.05689	0.012115	3.06E-06	13772	genus.LachnospiraceaeFCS020gr	TRUE	reported	rSCYwx	NA

rs9788306	T	C	-0.0628	0.013074	1.39E-06	13772	genus.LachnospiraceaeFCS020gr	TRUE	reported	rSCYwx	NA
rs6214092	T	C	0.061371	0.013105	2.38E-06	13771	genus.LachnospiraceaeFCS020gr	TRUE	reported	rSCYwx	NA
rs1138591	C	G	-0.10891	0.024205	2.55E-06	13238	genus.LachnospiraceaeFCS020gr	TRUE	reported	rSCYwx	NA
rs9919338	C	G	-0.05533	0.012062	4.91E-06	13768	genus.LachnospiraceaeFCS020gr	TRUE	reported	rSCYwx	NA
rs3503587	C	T	-0.19062	0.04144	2.62E-06	5642	genus.LachnospiraceaeFCS020gr	TRUE	reported	rSCYwx	NA
rs2862811	C	T	0.056493	0.012174	3.92E-06	13772	genus.LachnospiraceaeFCS020gr	TRUE	reported	rSCYwx	NA
rs1254846	A	G	0.105963	0.02325	5.60E-06	13172	genus.LachnospiraceaeFCS020gr	TRUE	reported	rSCYwx	NA
rs4452603	G	T	0.060421	0.013596	8.98E-06	13487	genus.LachnospiraceaeFCS020gr	TRUE	reported	rSCYwx	NA
rs2322265	T	C	-0.06663	0.014158	5.21E-06	13772	genus.LachnospiraceaeFCS020gr	TRUE	reported	rSCYwx	NA
rs3999074	T	G	-0.05506	0.012185	6.55E-06	13772	genus.LachnospiraceaeFCS020gr	TRUE	reported	rSCYwx	NA
rs9308097	G	A	0.055381	0.012366	7.47E-06	13542	genus.LachnospiraceaeFCS020gr	TRUE	reported	rSCYwx	NA
rs6116753	A	G	0.099475	0.020914	2.92E-06	6278	genus.LachnospiraceaeNC2004gr	TRUE	reported	GDobv5	NA
rs3756315	G	A	-0.08835	0.01884	3.33E-06	6328	genus.LachnospiraceaeNC2004gr	TRUE	reported	GDobv5	NA
rs1212773	A	G	0.115182	0.024601	3.11E-06	6226	genus.LachnospiraceaeNC2004gr	TRUE	reported	GDobv5	NA
rs1706707	A	G	-0.15463	0.035219	5.61E-06	4449	genus.LachnospiraceaeNC2004gr	TRUE	reported	GDobv5	NA
rs1331592	G	C	0.094881	0.020837	5.34E-06	6328	genus.LachnospiraceaeNC2004gr	TRUE	reported	GDobv5	NA
rs1928659	C	T	0.102522	0.022645	6.17E-06	5516	genus.LachnospiraceaeNC2004gr	TRUE	reported	GDobv5	NA
rs1929743	C	T	0.083721	0.019032	9.06E-06	6330	genus.LachnospiraceaeNC2004gr	TRUE	reported	GDobv5	NA
rs1286346	A	G	-0.15636	0.034537	6.04E-06	5516	genus.LachnospiraceaeNC2004gr	TRUE	reported	GDobv5	NA
rs1174676	C	T	-0.16969	0.038317	9.13E-06	5077	genus.LachnospiraceaeNC2004gr	TRUE	reported	GDobv5	NA
rs1220822	A	C	-0.15474	0.034037	9.75E-06	5620	genus.LachnospiraceaeNC2004gr	TRUE	reported	GDobv5	NA
rs9932954	G	A	-0.05619	0.011598	1.25E-06	15222	genus.LachnospiraceaeND3007gr	TRUE	reported	KB6ewd	NA
rs1311023	G	C	-0.0637	0.014204	4.48E-06	15019	genus.LachnospiraceaeND3007gr	TRUE	reported	KB6ewd	NA
rs7277667	C	T	-0.06471	0.014797	8.72E-06	15302	genus.LachnospiraceaeND3007gr	TRUE	reported	KB6ewd	NA
rs2861203	A	G	0.05723	0.012728	7.37E-06	15305	genus.LachnospiraceaeND3007gr	TRUE	reported	KB6ewd	NA
rs1236232	C	G	0.057317	0.011547	8.04E-07	16764	genus.LachnospiraceaeNK4A136g	TRUE	reported	xmbsp0	NA
rs954878	G	A	-0.05207	0.010908	1.78E-06	17227	genus.LachnospiraceaeNK4A136g	TRUE	reported	xmbsp0	NA
rs7832116	G	A	-0.07148	0.01517	3.57E-06	17219	genus.LachnospiraceaeNK4A136g	TRUE	reported	xmbsp0	NA
rs7616165	T	G	-0.23054	0.048346	2.77E-06	4094	genus.LachnospiraceaeNK4A136g	TRUE	reported	xmbsp0	NA
rs7619350	G	A	-0.22973	0.049978	2.93E-06	3578	genus.LachnospiraceaeNK4A136g	TRUE	reported	xmbsp0	NA
rs1126380	G	A	-0.05246	0.011676	5.07E-06	17236	genus.LachnospiraceaeNK4A136g	TRUE	reported	xmbsp0	NA
rs7304469	G	A	-0.10758	0.022988	3.57E-06	12773	genus.LachnospiraceaeNK4A136g	TRUE	reported	xmbsp0	NA
rs160061	G	A	0.051383	0.010809	2.12E-06	17234	genus.LachnospiraceaeNK4A136g	TRUE	reported	xmbsp0	NA

rs7073658	G	T	-0.04996	0.010969	5.27E-06	17236	genus.LachnospiraceaeNK4A136g	TRUE	reported	xmbsp0	NA
rs6810492	C	T	-0.05491	0.011538	2.37E-06	17227	genus.LachnospiraceaeNK4A136g	TRUE	reported	xmbsp0	NA
rs1261139	G	A	-0.09025	0.019965	5.83E-06	16486	genus.LachnospiraceaeNK4A136g	TRUE	reported	xmbsp0	NA
rs2880566	C	T	0.059958	0.013469	5.61E-06	17232	genus.LachnospiraceaeNK4A136g	TRUE	reported	xmbsp0	NA
rs2854083	C	A	0.050828	0.011059	9.34E-06	16764	genus.LachnospiraceaeNK4A136g	TRUE	reported	xmbsp0	NA
rs5980524	C	T	0.093616	0.020798	9.45E-06	15195	genus.LachnospiraceaeNK4A136g	TRUE	reported	xmbsp0	NA
rs1095211	T	G	0.048771	0.010961	9.08E-06	16764	genus.LachnospiraceaeNK4A136g	TRUE	reported	xmbsp0	NA
rs4955932	C	T	-0.04923	0.010939	7.05E-06	17236	genus.LachnospiraceaeNK4A136g	TRUE	reported	xmbsp0	NA
rs437876	C	T	0.078464	0.014477	7.17E-08	10150	genus.LachnospiraceaeUCG001.i	TRUE	reported	LSize9	NA
rs985416	T	C	0.097026	0.018181	1.46E-07	9917	genus.LachnospiraceaeUCG001.i	TRUE	reported	LSize9	NA
rs2050911	A	G	0.075147	0.015393	1.11E-06	10148	genus.LachnospiraceaeUCG001.i	TRUE	reported	LSize9	NA
rs1081557	G	C	-0.06837	0.014369	1.72E-06	10149	genus.LachnospiraceaeUCG001.i	TRUE	reported	LSize9	NA
rs573933	C	T	-0.1079	0.023235	3.11E-06	10150	genus.LachnospiraceaeUCG001.i	TRUE	reported	LSize9	NA
rs7884883	G	A	-0.11887	0.025975	3.38E-06	9506	genus.LachnospiraceaeUCG001.i	TRUE	reported	LSize9	NA
rs9403580	T	C	0.107801	0.022978	3.47E-06	10095	genus.LachnospiraceaeUCG001.i	TRUE	reported	LSize9	NA
rs7213933	A	T	-0.08163	0.01838	9.02E-06	9961	genus.LachnospiraceaeUCG001.i	TRUE	reported	LSize9	NA
rs8104225	G	A	0.08921	0.019766	8.04E-06	9908	genus.LachnospiraceaeUCG001.i	TRUE	reported	LSize9	NA
rs7403433	A	G	0.168045	0.038264	3.33E-06	6004	genus.LachnospiraceaeUCG001.i	TRUE	reported	LSize9	NA
rs6249641	G	T	-0.07485	0.016568	5.88E-06	10150	genus.LachnospiraceaeUCG001.i	TRUE	reported	LSize9	NA
rs2371284	C	T	-0.07619	0.017013	7.56E-06	9961	genus.LachnospiraceaeUCG001.i	TRUE	reported	LSize9	NA
rs1213122	T	C	0.117095	0.02591	7.40E-06	9676	genus.LachnospiraceaeUCG001.i	TRUE	reported	LSize9	NA
rs4981345	C	T	-0.0682	0.014983	6.09E-06	9961	genus.LachnospiraceaeUCG001.i	TRUE	reported	LSize9	NA
rs7947690	A	T	-0.08709	0.019701	8.27E-06	9917	genus.LachnospiraceaeUCG001.i	TRUE	reported	LSize9	NA
rs7341608	C	T	-0.07848	0.017765	9.48E-06	9917	genus.LachnospiraceaeUCG001.i	TRUE	reported	LSize9	NA
rs1274780	A	G	-0.0622	0.012568	8.65E-07	14464	genus.LachnospiraceaeUCG004.i	TRUE	reported	6LcZle	NA
rs2882478	A	G	-0.05771	0.011834	1.21E-06	14466	genus.LachnospiraceaeUCG004.i	TRUE	reported	6LcZle	NA
rs1267342	A	G	0.055435	0.011838	2.98E-06	14463	genus.LachnospiraceaeUCG004.i	TRUE	reported	6LcZle	NA
rs2444793	T	C	-0.05424	0.011819	4.77E-06	14463	genus.LachnospiraceaeUCG004.i	TRUE	reported	6LcZle	NA
rs2726805	G	A	0.05484	0.012084	6.30E-06	14466	genus.LachnospiraceaeUCG004.i	TRUE	reported	6LcZle	NA
rs1112818	G	A	0.064829	0.014013	4.52E-06	14466	genus.LachnospiraceaeUCG004.i	TRUE	reported	6LcZle	NA
rs1220225	A	G	-0.05509	0.012419	7.03E-06	14448	genus.LachnospiraceaeUCG004.i	TRUE	reported	6LcZle	NA
rs1289427	G	A	0.057979	0.012523	4.34E-06	14463	genus.LachnospiraceaeUCG004.i	TRUE	reported	6LcZle	NA
rs233486	G	A	-0.07991	0.017766	6.28E-06	13710	genus.LachnospiraceaeUCG004.i	TRUE	reported	6LcZle	NA

rs351821(	G	A	-0.10972	0.024221	4.87E-06	13170	genus.LachnospiraceaeUCG004.i	TRUE	reported	6LcZle	NA
rs6656451	T	C	-0.05436	0.011947	5.57E-06	14458	genus.LachnospiraceaeUCG004.i	TRUE	reported	6LcZle	NA
rs120725(	C	T	0.133104	0.030357	7.07E-06	9176	genus.LachnospiraceaeUCG004.i	TRUE	reported	6LcZle	NA
rs2706242	C	G	-0.09014	0.019971	9.84E-06	14026	genus.LachnospiraceaeUCG004.i	TRUE	reported	6LcZle	NA
rs6225651	A	G	0.059237	0.013222	9.85E-06	14026	genus.LachnospiraceaeUCG004.i	TRUE	reported	6LcZle	NA
rs7629954	G	A	0.108441	0.023825	5.77E-06	12568	genus.LachnospiraceaeUCG004.i	TRUE	reported	6LcZle	NA
rs107931(	T	C	0.097434	0.018128	9.35E-08	7014	genus.LachnospiraceaeUCG008.i	TRUE	reported	2N0Cw7	NA
rs9873555	C	G	-0.12116	0.023347	2.41E-07	6577	genus.LachnospiraceaeUCG008.i	TRUE	reported	2N0Cw7	NA
rs1302478	C	T	-0.07988	0.016885	2.29E-06	7016	genus.LachnospiraceaeUCG008.i	TRUE	reported	2N0Cw7	NA
rs1074177	C	T	-0.09738	0.019479	7.69E-07	7014	genus.LachnospiraceaeUCG008.i	TRUE	reported	2N0Cw7	NA
rs955844	C	A	0.112064	0.022837	1.81E-06	6863	genus.LachnospiraceaeUCG008.i	TRUE	reported	2N0Cw7	NA
rs6707883	C	T	-0.08458	0.017069	7.68E-07	7016	genus.LachnospiraceaeUCG008.i	TRUE	reported	2N0Cw7	NA
rs1075123	C	A	-0.08207	0.017438	3.17E-06	6930	genus.LachnospiraceaeUCG008.i	TRUE	reported	2N0Cw7	NA
rs6227784	T	C	0.102292	0.021232	1.59E-06	7016	genus.LachnospiraceaeUCG008.i	TRUE	reported	2N0Cw7	NA
rs108018(	A	G	-0.11703	0.024308	1.40E-06	7017	genus.LachnospiraceaeUCG008.i	TRUE	reported	2N0Cw7	NA
rs6194477	G	A	0.179838	0.03938	6.34E-06	6000	genus.LachnospiraceaeUCG008.i	TRUE	reported	2N0Cw7	NA
rs7535664	A	G	0.136523	0.030313	9.83E-06	6423	genus.LachnospiraceaeUCG008.i	TRUE	reported	2N0Cw7	NA
rs1123621	C	T	0.083842	0.017671	2.35E-06	6930	genus.LachnospiraceaeUCG008.i	TRUE	reported	2N0Cw7	NA
rs5725447	A	G	0.088651	0.019905	6.92E-06	7017	genus.LachnospiraceaeUCG008.i	TRUE	reported	2N0Cw7	NA
rs5709157	G	A	-0.11044	0.02358	2.86E-06	6950	genus.LachnospiraceaeUCG008.i	TRUE	reported	2N0Cw7	NA
rs1119244	G	A	0.126592	0.024345	4.69E-07	12695	genus.LachnospiraceaeUCG010.i	TRUE	reported	rDCjKX	NA
rs9981767	C	A	0.065506	0.013199	9.96E-07	12816	genus.LachnospiraceaeUCG010.i	TRUE	reported	rDCjKX	NA
rs743158(	T	G	0.086726	0.018348	3.19E-06	12715	genus.LachnospiraceaeUCG010.i	TRUE	reported	rDCjKX	NA
rs1234665	T	C	0.065769	0.013957	2.70E-06	12816	genus.LachnospiraceaeUCG010.i	TRUE	reported	rDCjKX	NA
rs1041481	C	T	0.10453	0.023025	4.24E-06	11564	genus.LachnospiraceaeUCG010.i	TRUE	reported	rDCjKX	NA
rs7276182	T	A	0.111884	0.023889	2.58E-06	11488	genus.LachnospiraceaeUCG010.i	TRUE	reported	rDCjKX	NA
rs7289495	A	G	0.222263	0.048643	5.68E-06	4316	genus.LachnospiraceaeUCG010.i	TRUE	reported	rDCjKX	NA
rs336138	T	G	0.077954	0.017187	7.48E-06	13236	genus.LachnospiraceaeUCG010.i	TRUE	reported	rDCjKX	NA
rs4576377	C	A	-0.0572	0.012704	7.63E-06	13225	genus.LachnospiraceaeUCG010.i	TRUE	reported	rDCjKX	NA
rs2833528	T	C	-0.05621	0.012776	9.92E-06	13233	genus.LachnospiraceaeUCG010.i	TRUE	reported	rDCjKX	NA
rs1408275	T	A	-0.06156	0.013873	8.05E-06	12816	genus.LachnospiraceaeUCG010.i	TRUE	reported	rDCjKX	NA
rs215346(	T	A	-0.0684	0.015656	9.17E-06	13236	genus.LachnospiraceaeUCG010.i	TRUE	reported	rDCjKX	NA
rs1773001	A	G	-0.07024	0.015706	7.85E-06	13226	genus.LachnospiraceaeUCG010.i	TRUE	reported	rDCjKX	NA

rs921925	C	A	0.098508	0.020323	9.72E-07	6952	genus.Lactobacillus.id.1837.s	TRUE	reported	17J2Sq	NA
rs168616(	A	G	-0.18315	0.038148	1.28E-06	5325	genus.Lactobacillus.id.1837.s	TRUE	reported	17J2Sq	NA
rs768253	G	T	-0.0792	0.017179	4.25E-06	6958	genus.Lactobacillus.id.1837.s	TRUE	reported	17J2Sq	NA
rs116748!	T	C	-0.08527	0.01765	1.59E-06	6655	genus.Lactobacillus.id.1837.s	TRUE	reported	17J2Sq	NA
rs328312	A	T	0.081509	0.016944	1.41E-06	6950	genus.Lactobacillus.id.1837.s	TRUE	reported	17J2Sq	NA
rs609214(	T	A	-0.08013	0.017149	3.29E-06	6906	genus.Lactobacillus.id.1837.s	TRUE	reported	17J2Sq	NA
rs751276(	A	C	0.139784	0.031041	6.83E-06	6840	genus.Lactobacillus.id.1837.s	TRUE	reported	17J2Sq	NA
rs774787!	G	A	-0.21989	0.047576	7.33E-06	3669	genus.Lactobacillus.id.1837.s	TRUE	reported	17J2Sq	NA
rs153055(	A	G	0.0804	0.017821	4.93E-06	6655	genus.Lactobacillus.id.1837.s	TRUE	reported	17J2Sq	NA
rs623146!	A	C	0.187692	0.039458	2.24E-06	5944	genus.Lactobacillus.id.1837.s	TRUE	reported	17J2Sq	NA
rs739965{	A	G	-0.10713	0.022188	3.12E-06	6613	genus.Lactobacillus.id.1837.s	TRUE	reported	17J2Sq	NA
rs126938	T	C	-0.08054	0.017743	8.96E-06	6958	genus.Lactobacillus.id.1837.s	TRUE	reported	17J2Sq	NA
rs347579{	C	G	0.122294	0.022898	8.95E-08	4055	genus.Lactococcus.id.1851.sum	TRUE	reported	5Q22Hh	NA
rs757872	C	G	0.140799	0.027582	4.37E-07	3792	genus.Lactococcus.id.1851.sum	TRUE	reported	5Q22Hh	NA
rs123059	C	T	-0.13671	0.027469	1.27E-06	3760	genus.Lactococcus.id.1851.sum	TRUE	reported	5Q22Hh	NA
rs476699	T	C	0.114599	0.023839	2.06E-06	4053	genus.Lactococcus.id.1851.sum	TRUE	reported	5Q22Hh	NA
rs104178	G	T	0.118306	0.024522	1.29E-06	4056	genus.Lactococcus.id.1851.sum	TRUE	reported	5Q22Hh	NA
rs667430	T	C	0.200758	0.044212	6.18E-06	3869	genus.Lactococcus.id.1851.sum	TRUE	reported	5Q22Hh	NA
rs799224(	C	T	0.104232	0.023079	4.45E-06	4057	genus.Lactococcus.id.1851.sum	TRUE	reported	5Q22Hh	NA
rs559101(	T	C	0.146426	0.030737	2.36E-06	3836	genus.Lactococcus.id.1851.sum	TRUE	reported	5Q22Hh	NA
rs126218	A	G	0.108423	0.023998	6.61E-06	4057	genus.Lactococcus.id.1851.sum	TRUE	reported	5Q22Hh	NA
rs229336	T	C	-0.19922	0.043097	1.40E-06	3787	genus.Lactococcus.id.1851.sum	TRUE	reported	5Q22Hh	NA
rs171683(	A	G	0.191857	0.042476	6.29E-06	4023	genus.Lactococcus.id.1851.sum	TRUE	reported	5Q22Hh	NA
rs618844	A	G	0.124426	0.024843	1.01E-06	11559	genus.Marvinbryantia.id.2005.	TRUE	reported	JzCX8b	NA
rs272481:	G	A	-0.08408	0.016755	6.28E-07	11610	genus.Marvinbryantia.id.2005.	TRUE	reported	JzCX8b	NA
rs118798:	T	C	-0.09355	0.019317	2.02E-06	11896	genus.Marvinbryantia.id.2005.	TRUE	reported	JzCX8b	NA
rs284289(	T	C	-0.06494	0.013115	7.25E-07	11709	genus.Marvinbryantia.id.2005.	TRUE	reported	JzCX8b	NA
rs116450	C	G	-0.06062	0.013155	4.15E-06	11709	genus.Marvinbryantia.id.2005.	TRUE	reported	JzCX8b	NA
rs129633	C	G	-0.05968	0.013229	6.60E-06	11709	genus.Marvinbryantia.id.2005.	TRUE	reported	JzCX8b	NA
rs286336:	G	A	0.063486	0.013632	3.11E-06	11950	genus.Marvinbryantia.id.2005.	TRUE	reported	JzCX8b	NA
rs729482	C	A	-0.12635	0.027221	3.26E-06	11561	genus.Marvinbryantia.id.2005.	TRUE	reported	JzCX8b	NA
rs399104{	T	G	0.059146	0.013254	7.09E-06	11939	genus.Marvinbryantia.id.2005.	TRUE	reported	JzCX8b	NA
rs146541	A	G	0.118845	0.026842	6.86E-06	10778	genus.Marvinbryantia.id.2005.	TRUE	reported	JzCX8b	NA

rs1162059	C	T	0.119479	0.027169	7.80E-06	11031	genus.Marvinbryantia.id.2005.	TRUE	reported	JzCX8b	NA
rs8006832	T	G	-0.09524	0.02167	6.58E-06	11412	genus.Marvinbryantia.id.2005.	TRUE	reported	JzCX8b	NA
rs3125832	C	A	0.067932	0.015012	5.03E-06	11709	genus.Marvinbryantia.id.2005.	TRUE	reported	JzCX8b	NA
rs7602931	C	T	0.222849	0.045432	1.08E-06	3387	genus.Methanobrevibacter.id.1	TRUE	reported	j852P6	NA
rs1020290	G	T	-0.11281	0.023911	3.09E-06	3583	genus.Methanobrevibacter.id.1	TRUE	reported	j852P6	NA
rs894996	A	C	0.214213	0.045604	3.82E-06	3479	genus.Methanobrevibacter.id.1	TRUE	reported	j852P6	NA
rs1101866	T	A	0.113013	0.025434	7.03E-06	3586	genus.Methanobrevibacter.id.1	TRUE	reported	j852P6	NA
rs1334944	C	T	0.115197	0.025549	7.61E-06	3586	genus.Methanobrevibacter.id.1	TRUE	reported	j852P6	NA
rs6776814	C	T	-0.18896	0.041991	8.05E-06	3283	genus.Methanobrevibacter.id.1	TRUE	reported	j852P6	NA
rs4779844	C	G	0.109639	0.024798	9.28E-06	3585	genus.Methanobrevibacter.id.1	TRUE	reported	j852P6	NA
rs4802933	G	A	-0.13563	0.030814	9.74E-06	3586	genus.Methanobrevibacter.id.1	TRUE	reported	j852P6	NA
rs503751	G	C	0.061829	0.011889	2.08E-07	14218	genus.Odoribacter.id.952.summ	TRUE	reported	x9TMdf	NA
rs7777948	A	G	-0.13349	0.026853	6.56E-07	11935	genus.Odoribacter.id.952.summ	TRUE	reported	x9TMdf	NA
rs1042379	T	C	0.055069	0.012116	6.58E-06	14135	genus.Odoribacter.id.952.summ	TRUE	reported	x9TMdf	NA
rs6856150	A	G	0.088192	0.019415	6.06E-06	14597	genus.Odoribacter.id.952.summ	TRUE	reported	x9TMdf	NA
rs1009386	G	A	-0.05778	0.012539	3.67E-06	14218	genus.Odoribacter.id.952.summ	TRUE	reported	x9TMdf	NA
rs4793970	G	A	-0.05763	0.012915	6.03E-06	14687	genus.Odoribacter.id.952.summ	TRUE	reported	x9TMdf	NA
rs7455396	G	T	0.121449	0.026411	9.49E-06	11803	genus.Odoribacter.id.952.summ	TRUE	reported	x9TMdf	NA
rs2841740	G	A	-0.07269	0.016137	3.68E-06	14698	genus.Odoribacter.id.952.summ	TRUE	reported	x9TMdf	NA
rs1691842	T	A	0.099978	0.022378	8.85E-06	13689	genus.Odoribacter.id.952.summ	TRUE	reported	x9TMdf	NA
rs1035588	G	A	-0.10815	0.023685	4.86E-06	3739	genus.Olsenella.id.822.summar	TRUE	reported	pDXde1	NA
rs6211253	T	C	-0.19943	0.040703	1.19E-06	3630	genus.Olsenella.id.822.summar	TRUE	reported	pDXde1	NA
rs3522586	G	A	-0.2236	0.048239	3.87E-06	3630	genus.Olsenella.id.822.summar	TRUE	reported	pDXde1	NA
rs7269158	A	C	-0.24908	0.052081	2.95E-06	3078	genus.Olsenella.id.822.summar	TRUE	reported	pDXde1	NA
rs9460691	A	C	0.119966	0.026864	7.28E-06	3739	genus.Olsenella.id.822.summar	TRUE	reported	pDXde1	NA
rs1714876	A	G	0.140434	0.02956	2.20E-06	3648	genus.Olsenella.id.822.summar	TRUE	reported	pDXde1	NA
rs6109014	G	A	-0.10478	0.023134	6.44E-06	3739	genus.Olsenella.id.822.summar	TRUE	reported	pDXde1	NA
rs7540303	T	C	0.108044	0.023638	5.32E-06	3739	genus.Olsenella.id.822.summar	TRUE	reported	pDXde1	NA
rs2759329	A	G	-0.11113	0.023722	3.43E-06	3739	genus.Olsenella.id.822.summar	TRUE	reported	pDXde1	NA
rs6046522	T	C	0.122938	0.026993	4.48E-06	3705	genus.Olsenella.id.822.summar	TRUE	reported	pDXde1	NA
rs8066522	A	G	-0.10653	0.024038	9.70E-06	3739	genus.Olsenella.id.822.summar	TRUE	reported	pDXde1	NA
rs234108	G	A	0.074955	0.015263	9.16E-07	8694	genus.Oscillibacter.id.2063.s	TRUE	reported	iiflrl	NA
rs3609527	T	C	-0.07524	0.015686	1.40E-06	8694	genus.Oscillibacter.id.2063.s	TRUE	reported	iiflrl	NA

rs1162762	C	T	0.143961	0.029022	1.01E-06	8486	genus.Oscillibacter.id.2063.s	TRUE	reported iiflrl	NA
rs9393920	G	A	-0.07447	0.015108	9.92E-07	8930	genus.Oscillibacter.id.2063.s	TRUE	reported iiflrl	NA
rs133832	C	A	-0.07955	0.016241	1.15E-06	8926	genus.Oscillibacter.id.2063.s	TRUE	reported iiflrl	NA
rs1264993	G	T	0.121589	0.025961	4.09E-06	8794	genus.Oscillibacter.id.2063.s	TRUE	reported iiflrl	NA
rs7545376	T	G	0.12212	0.026863	5.35E-06	8256	genus.Oscillibacter.id.2063.s	TRUE	reported iiflrl	NA
rs1686640	G	A	0.098877	0.02088	3.08E-06	8930	genus.Oscillibacter.id.2063.s	TRUE	reported iiflrl	NA
rs761240	G	T	-0.17664	0.038881	2.04E-06	5185	genus.Oscillibacter.id.2063.s	TRUE	reported iiflrl	NA
rs4506202	G	A	-0.07113	0.015226	3.21E-06	8694	genus.Oscillibacter.id.2063.s	TRUE	reported iiflrl	NA
rs6188356	G	A	-0.10135	0.022102	3.39E-06	8930	genus.Oscillibacter.id.2063.s	TRUE	reported iiflrl	NA
rs1693418	G	A	-0.12957	0.028157	4.38E-06	8492	genus.Oscillibacter.id.2063.s	TRUE	reported iiflrl	NA
rs1241795	G	C	0.078543	0.017407	6.03E-06	8928	genus.Oscillibacter.id.2063.s	TRUE	reported iiflrl	NA
rs6901560	G	C	0.085503	0.018693	6.21E-06	8525	genus.Oscillibacter.id.2063.s	TRUE	reported iiflrl	NA
rs6220650	A	C	-0.06814	0.015135	6.60E-06	8883	genus.Oscillibacter.id.2063.s	TRUE	reported iiflrl	NA
rs1379171	A	T	-0.17499	0.038839	4.62E-06	5733	genus.Oscillibacter.id.2063.s	TRUE	reported iiflrl	NA
rs1199027	C	T	-0.08249	0.018046	4.94E-06	8528	genus.Oscillibacter.id.2063.s	TRUE	reported iiflrl	NA
rs1220646	A	G	-0.13302	0.026973	1.04E-06	9554	genus.Oscillospira.id.2064.su	TRUE	reported DcCeSu	NA
rs7303867	A	T	-0.08321	0.016867	1.09E-06	10195	genus.Oscillospira.id.2064.su	TRUE	reported DcCeSu	NA
rs1954532	C	T	-0.08262	0.017525	2.27E-06	9841	genus.Oscillospira.id.2064.su	TRUE	reported DcCeSu	NA
rs2888993	C	A	0.114051	0.025284	3.37E-06	9758	genus.Oscillospira.id.2064.su	TRUE	reported DcCeSu	NA
rs751183	C	T	-0.07743	0.017222	6.85E-06	9841	genus.Oscillospira.id.2064.su	TRUE	reported DcCeSu	NA
rs8076323	G	A	0.071539	0.015654	5.61E-06	10193	genus.Oscillospira.id.2064.su	TRUE	reported DcCeSu	NA
rs7286697	C	A	-0.13057	0.028167	5.63E-06	9098	genus.Oscillospira.id.2064.su	TRUE	reported DcCeSu	NA
rs6242265	T	C	0.089846	0.019809	6.47E-06	9769	genus.Oscillospira.id.2064.su	TRUE	reported DcCeSu	NA
rs2211563	A	C	0.081918	0.018108	9.12E-06	10199	genus.Oscillospira.id.2064.su	TRUE	reported DcCeSu	NA
rs1292502	C	T	0.13559	0.030678	9.31E-06	8200	genus.Oscillospira.id.2064.su	TRUE	reported DcCeSu	NA
rs4428215	A	G	0.130293	0.024224	7.51E-08	4655	genus.Oxalobacter.id.2978.sum	TRUE	reported N10WJM	NA
rs736744	T	C	0.117882	0.021126	2.57E-08	4655	genus.Oxalobacter.id.2978.sum	TRUE	reported N10WJM	NA
rs6000536	T	C	-0.13099	0.02538	2.06E-07	4654	genus.Oxalobacter.id.2978.sum	TRUE	reported N10WJM	NA
rs3605733	T	G	0.207847	0.042144	8.80E-07	4244	genus.Oxalobacter.id.2978.sum	TRUE	reported N10WJM	NA
rs6071435	A	T	-0.10551	0.021489	1.07E-06	4635	genus.Oxalobacter.id.2978.sum	TRUE	reported N10WJM	NA
rs1200225	C	A	0.217122	0.046632	1.42E-06	4297	genus.Oxalobacter.id.2978.sum	TRUE	reported N10WJM	NA
rs1569853	C	T	-0.13808	0.029698	3.65E-06	4492	genus.Oxalobacter.id.2978.sum	TRUE	reported N10WJM	NA
rs1110850	G	A	-0.1991	0.042733	3.74E-06	4303	genus.Oxalobacter.id.2978.sum	TRUE	reported N10WJM	NA

rs1046499	A	G	0.137691	0.02948	3.30E-06	4650	genus.Oxalobacter.id.2978.sum	TRUE	reported	N10WJM	NA
rs1119667	C	T	0.213114	0.047162	7.30E-06	3931	genus.Oxalobacter.id.2978.sum	TRUE	reported	N10WJM	NA
rs6993398	A	G	0.127217	0.027885	7.13E-06	4656	genus.Oxalobacter.id.2978.sum	TRUE	reported	N10WJM	NA
rs3862635	T	C	-0.17214	0.039403	9.19E-06	4469	genus.Oxalobacter.id.2978.sum	TRUE	reported	N10WJM	NA
rs6088475	T	C	-0.07027	0.014225	5.71E-07	17293	genus.Parabacteroides.id.954.	TRUE	reported	3BC0HR	NA
rs3860755	C	G	0.055878	0.011662	1.71E-06	17294	genus.Parabacteroides.id.954.	TRUE	reported	3BC0HR	NA
rs4236095	A	G	0.076196	0.015704	1.93E-06	16383	genus.Parabacteroides.id.954.	TRUE	reported	3BC0HR	NA
rs1156028	A	G	0.10308	0.022274	1.93E-06	16394	genus.Parabacteroides.id.954.	TRUE	reported	3BC0HR	NA
rs1145673	C	T	0.186478	0.04052	5.65E-06	5859	genus.Parabacteroides.id.954.	TRUE	reported	3BC0HR	NA
rs7298818	T	C	0.088883	0.02009	8.54E-06	16510	genus.Parabacteroides.id.954.	TRUE	reported	3BC0HR	NA
rs6657302	C	T	-0.10452	0.022552	9.76E-06	16552	genus.Parabacteroides.id.954.	TRUE	reported	3BC0HR	NA
rs1196557	C	G	0.162824	0.038186	8.87E-06	3890	genus.Parabacteroides.id.954.	TRUE	reported	3BC0HR	NA
rs1714198	C	G	0.05003	0.011146	7.27E-06	16825	genus.Parabacteroides.id.954.	TRUE	reported	3BC0HR	NA
rs7289364	T	A	-0.07156	0.015667	8.83E-06	16508	genus.Parabacteroides.id.954.	TRUE	reported	3BC0HR	NA
rs2081023	G	A	-0.12256	0.023651	2.64E-07	7178	genus.Paraprevotella.id.962.s	TRUE	reported	B341QM	NA
rs9900242	G	A	-0.0853	0.017521	1.14E-06	6900	genus.Paraprevotella.id.962.s	TRUE	reported	B341QM	NA
rs9602779	C	A	-0.10669	0.022026	6.93E-07	7177	genus.Paraprevotella.id.962.s	TRUE	reported	B341QM	NA
rs1409979	C	T	-0.16238	0.035418	2.11E-06	6187	genus.Paraprevotella.id.962.s	TRUE	reported	B341QM	NA
rs1450203	G	A	-0.12465	0.026232	4.03E-06	6859	genus.Paraprevotella.id.962.s	TRUE	reported	B341QM	NA
rs4767113	T	C	0.088247	0.018382	2.14E-06	6900	genus.Paraprevotella.id.962.s	TRUE	reported	B341QM	NA
rs3008582	C	T	0.10572	0.022725	4.36E-06	7175	genus.Paraprevotella.id.962.s	TRUE	reported	B341QM	NA
rs4756632	T	G	-0.13891	0.02899	3.82E-06	7179	genus.Paraprevotella.id.962.s	TRUE	reported	B341QM	NA
rs1084246	C	T	-0.07582	0.017257	6.60E-06	7178	genus.Paraprevotella.id.962.s	TRUE	reported	B341QM	NA
rs3801748	A	G	0.077972	0.017169	5.20E-06	7179	genus.Paraprevotella.id.962.s	TRUE	reported	B341QM	NA
rs7240324	G	T	-0.10229	0.022695	5.96E-06	6900	genus.Paraprevotella.id.962.s	TRUE	reported	B341QM	NA
rs1710992	G	A	-0.09883	0.021617	6.75E-06	7179	genus.Paraprevotella.id.962.s	TRUE	reported	B341QM	NA
rs1778562	G	A	0.248065	0.05243	1.93E-06	3288	genus.Paraprevotella.id.962.s	TRUE	reported	B341QM	NA
rs2387977	C	T	-0.06818	0.013476	5.38E-07	11345	genus.Parasutterella.id.2892.	TRUE	reported	nh1V6A	NA
rs7572229	A	G	0.066273	0.013274	6.32E-07	11383	genus.Parasutterella.id.2892.	TRUE	reported	nh1V6A	NA
rs3541459	A	T	-0.06849	0.014214	1.51E-06	11386	genus.Parasutterella.id.2892.	TRUE	reported	nh1V6A	NA
rs6828768	T	C	0.063685	0.013265	1.78E-06	11386	genus.Parasutterella.id.2892.	TRUE	reported	nh1V6A	NA
rs1089991	G	A	-0.07171	0.014815	1.15E-06	11386	genus.Parasutterella.id.2892.	TRUE	reported	nh1V6A	NA
rs7303158	T	C	0.064686	0.013426	1.33E-06	11386	genus.Parasutterella.id.2892.	TRUE	reported	nh1V6A	NA

rs7838303	C	T	-0.14631	0.029712	1.57E-06	9174	genus.Parasutterella.id.2892.	TRUE	reported	nh1V6A	NA
rs3505555	C	T	0.109554	0.023543	3.35E-06	9970	genus.Parasutterella.id.2892.	TRUE	reported	nh1V6A	NA
rs2090816	C	A	0.084097	0.017731	2.90E-06	11385	genus.Parasutterella.id.2892.	TRUE	reported	nh1V6A	NA
rs1403396	T	A	-0.07567	0.015903	2.76E-06	11378	genus.Parasutterella.id.2892.	TRUE	reported	nh1V6A	NA
rs5587786	C	A	-0.10446	0.022809	2.87E-06	10588	genus.Parasutterella.id.2892.	TRUE	reported	nh1V6A	NA
rs8039785	G	T	0.061835	0.0133	3.62E-06	11383	genus.Parasutterella.id.2892.	TRUE	reported	nh1V6A	NA
rs823424	A	G	-0.07134	0.015696	4.95E-06	11386	genus.Parasutterella.id.2892.	TRUE	reported	nh1V6A	NA
rs7311004	C	T	-0.06175	0.013644	5.92E-06	11384	genus.Parasutterella.id.2892.	TRUE	reported	nh1V6A	NA
rs6227390	G	A	0.229468	0.050226	5.88E-06	3691	genus.Parasutterella.id.2892.	TRUE	reported	nh1V6A	NA
rs6809952	A	G	-0.0685	0.015089	8.13E-06	11077	genus.Parasutterella.id.2892.	TRUE	reported	nh1V6A	NA
rs1171585	A	G	-0.0663	0.014611	6.23E-06	11386	genus.Parasutterella.id.2892.	TRUE	reported	nh1V6A	NA
rs7575456	G	C	0.181434	0.031943	1.10E-08	5481	genus.Peptococcus.id.2037.sum	TRUE	reported	ovhlml	NA
rs7768162	T	C	0.200307	0.038733	2.69E-07	4842	genus.Peptococcus.id.2037.sum	TRUE	reported	ovhlml	NA
rs1003105	C	T	-0.12117	0.022584	1.24E-07	5526	genus.Peptococcus.id.2037.sum	TRUE	reported	ovhlml	NA
rs6918730	A	G	0.135311	0.028974	1.15E-06	5656	genus.Peptococcus.id.2037.sum	TRUE	reported	ovhlml	NA
rs413827	A	G	0.110229	0.023752	3.30E-06	5482	genus.Peptococcus.id.2037.sum	TRUE	reported	ovhlml	NA
rs2054133	A	G	0.089543	0.018833	2.14E-06	5657	genus.Peptococcus.id.2037.sum	TRUE	reported	ovhlml	NA
rs1100194	A	G	-0.19561	0.039222	1.33E-06	5607	genus.Peptococcus.id.2037.sum	TRUE	reported	ovhlml	NA
rs7033353	G	T	0.090152	0.018995	2.22E-06	5657	genus.Peptococcus.id.2037.sum	TRUE	reported	ovhlml	NA
rs5770862	C	T	0.162018	0.035681	3.22E-06	5431	genus.Peptococcus.id.2037.sum	TRUE	reported	ovhlml	NA
rs3428274	C	G	0.191797	0.039983	1.84E-06	5257	genus.Peptococcus.id.2037.sum	TRUE	reported	ovhlml	NA
rs7285016	C	T	-0.1343	0.030042	5.74E-06	5627	genus.Peptococcus.id.2037.sum	TRUE	reported	ovhlml	NA
rs7761420	C	T	-0.17586	0.038752	5.68E-06	5329	genus.Peptococcus.id.2037.sum	TRUE	reported	ovhlml	NA
rs1103056	T	A	-0.17402	0.037413	3.13E-06	5329	genus.Peptococcus.id.2037.sum	TRUE	reported	ovhlml	NA
rs7766680	C	G	0.097683	0.021415	3.51E-06	5655	genus.Peptococcus.id.2037.sum	TRUE	reported	ovhlml	NA
rs7459222	A	G	0.137957	0.030296	8.55E-06	5329	genus.Peptococcus.id.2037.sum	TRUE	reported	ovhlml	NA
rs3612107	G	A	-0.14067	0.030628	6.99E-06	5301	genus.Peptococcus.id.2037.sum	TRUE	reported	ovhlml	NA
rs7669771	A	C	0.146302	0.032234	8.05E-06	5481	genus.Peptococcus.id.2037.sum	TRUE	reported	ovhlml	NA
rs1206935	T	C	0.16763	0.03795	9.28E-06	5352	genus.Peptococcus.id.2037.sum	TRUE	reported	ovhlml	NA
rs7588296	C	T	0.096866	0.019058	3.19E-07	10710	genus.Phascolarctobacterium.i	TRUE	reported	R9mAqY	NA
rs5615788	C	A	0.095486	0.019398	1.09E-06	10031	genus.Phascolarctobacterium.i	TRUE	reported	R9mAqY	NA
rs5606906	A	G	-0.11131	0.023069	1.87E-06	11007	genus.Phascolarctobacterium.i	TRUE	reported	R9mAqY	NA
rs7454077	A	G	-0.12101	0.025862	3.60E-06	9403	genus.Phascolarctobacterium.i	TRUE	reported	R9mAqY	NA

rs7612421	G	C	-0.15936	0.034453	2.67E-06	7314	genus.Phascolarctobacterium.i	TRUE	reported	R9mAqY	NA
rs6427992	C	G	-0.06525	0.013751	2.09E-06	11113	genus.Phascolarctobacterium.i	TRUE	reported	R9mAqY	NA
rs1261820	G	A	0.064166	0.01382	3.38E-06	11113	genus.Phascolarctobacterium.i	TRUE	reported	R9mAqY	NA
rs7484727	G	A	-0.10489	0.0231	5.73E-06	11098	genus.Phascolarctobacterium.i	TRUE	reported	R9mAqY	NA
rs1264476	G	T	0.076734	0.016604	4.30E-06	11104	genus.Phascolarctobacterium.i	TRUE	reported	R9mAqY	NA
rs2852513	A	G	-0.11866	0.026905	8.23E-06	10457	genus.Phascolarctobacterium.i	TRUE	reported	R9mAqY	NA
rs7982713	A	G	0.072692	0.01632	9.72E-06	10710	genus.Phascolarctobacterium.i	TRUE	reported	R9mAqY	NA
rs1192984	C	T	-0.06972	0.015797	8.88E-06	11114	genus.Phascolarctobacterium.i	TRUE	reported	R9mAqY	NA
rs130483	G	A	0.065506	0.01436	6.79E-06	11096	genus.Phascolarctobacterium.i	TRUE	reported	R9mAqY	NA
rs430270	C	A	0.139155	0.029706	2.87E-06	3318	genus.Prevotella7.id.11182.su	TRUE	reported	8Z7K06	NA
rs5740456	A	C	0.155486	0.031606	6.22E-07	3487	genus.Prevotella7.id.11182.su	TRUE	reported	8Z7K06	NA
rs9959718	A	G	0.133012	0.027536	1.90E-06	3489	genus.Prevotella7.id.11182.su	TRUE	reported	8Z7K06	NA
rs9608249	G	A	-0.15821	0.033632	2.07E-06	3482	genus.Prevotella7.id.11182.su	TRUE	reported	8Z7K06	NA
rs2240542	T	C	0.12085	0.026178	4.84E-06	3489	genus.Prevotella7.id.11182.su	TRUE	reported	8Z7K06	NA
rs2918132	T	C	-0.11465	0.025492	6.42E-06	3318	genus.Prevotella7.id.11182.su	TRUE	reported	8Z7K06	NA
rs1693724	C	G	0.146098	0.035153	9.64E-06	3486	genus.Prevotella7.id.11182.su	TRUE	reported	8Z7K06	NA
rs1180384	G	A	0.205676	0.046892	7.85E-06	3295	genus.Prevotella7.id.11182.su	TRUE	reported	8Z7K06	NA
rs1212456	G	A	-0.12128	0.027502	9.49E-06	3283	genus.Prevotella7.id.11182.su	TRUE	reported	8Z7K06	NA
rs1219543	C	T	0.196528	0.044232	8.73E-06	3489	genus.Prevotella7.id.11182.su	TRUE	reported	8Z7K06	NA
rs9426434	C	T	-0.12366	0.027851	9.72E-06	3318	genus.Prevotella7.id.11182.su	TRUE	reported	8Z7K06	NA
rs7926316	C	A	-0.14398	0.031501	7.51E-06	3489	genus.Prevotella7.id.11182.su	TRUE	reported	8Z7K06	NA
rs1115098	C	T	0.171131	0.034762	1.24E-06	6960	genus.Prevotella9.id.11183.su	TRUE	reported	9j4pH8	NA
rs2683313	G	A	-0.07246	0.01516	1.69E-06	10260	genus.Prevotella9.id.11183.su	TRUE	reported	9j4pH8	NA
rs1172719	G	A	0.208089	0.044039	2.82E-06	4878	genus.Prevotella9.id.11183.su	TRUE	reported	9j4pH8	NA
rs1051234	G	C	0.247373	0.054409	3.19E-06	3063	genus.Prevotella9.id.11183.su	TRUE	reported	9j4pH8	NA
rs9428102	G	A	-0.07788	0.017607	4.62E-06	10264	genus.Prevotella9.id.11183.su	TRUE	reported	9j4pH8	NA
rs746764	C	T	-0.09158	0.019324	2.04E-06	9385	genus.Prevotella9.id.11183.su	TRUE	reported	9j4pH8	NA
rs1696646	C	G	0.074335	0.016529	9.33E-06	10261	genus.Prevotella9.id.11183.su	TRUE	reported	9j4pH8	NA
rs2104588	C	T	0.105558	0.023773	8.13E-06	9268	genus.Prevotella9.id.11183.su	TRUE	reported	9j4pH8	NA
rs1119973	T	A	0.077021	0.016946	7.00E-06	10263	genus.Prevotella9.id.11183.su	TRUE	reported	9j4pH8	NA
rs7232121	C	G	0.067129	0.014423	3.76E-06	9790	genus.Prevotella9.id.11183.su	TRUE	reported	9j4pH8	NA
rs1168569	T	C	-0.14136	0.029566	2.03E-06	8342	genus.Prevotella9.id.11183.su	TRUE	reported	9j4pH8	NA
rs9613013	A	G	0.091759	0.02027	6.10E-06	9790	genus.Prevotella9.id.11183.su	TRUE	reported	9j4pH8	NA

rs2495052	G	A	0.083898	0.018852	8.97E-06	9790	genus.Prevotella9.id.11183.su	TRUE	reported	9j4pH8	NA
rs7281577	C	T	-0.17619	0.039307	8.78E-06	5728	genus.Prevotella9.id.11183.su	TRUE	reported	9j4pH8	NA
rs4968431	T	G	0.064011	0.014416	8.58E-06	10271	genus.Prevotella9.id.11183.su	TRUE	reported	9j4pH8	NA
rs4821647	C	G	0.063696	0.014398	9.95E-06	10179	genus.Prevotella9.id.11183.su	TRUE	reported	9j4pH8	NA
rs7976209	C	T	-0.08708	0.019795	7.28E-06	10263	genus.Prevotella9.id.11183.su	TRUE	reported	9j4pH8	NA
rs1264823	C	T	0.07859	0.017768	7.39E-06	10271	genus.Prevotella9.id.11183.su	TRUE	reported	9j4pH8	NA
rs1304512	A	G	0.076159	0.016588	5.29E-06	9790	genus.Prevotella9.id.11183.su	TRUE	reported	9j4pH8	NA
rs7237249	T	C	-0.08238	0.018213	8.93E-06	10271	genus.Prevotella9.id.11183.su	TRUE	reported	9j4pH8	NA
rs2900503	T	G	-0.17233	0.032667	1.55E-07	3320	genus.RikenellaceaeRC9gutgrou	TRUE	reported	RlaPhE	NA
rs1758278	G	A	-0.15774	0.033992	3.55E-06	3320	genus.RikenellaceaeRC9gutgrou	TRUE	reported	RlaPhE	NA
rs7113155	C	G	0.114423	0.02478	5.26E-06	3334	genus.RikenellaceaeRC9gutgrou	TRUE	reported	RlaPhE	NA
rs4270579	A	G	-0.11802	0.027104	5.46E-06	3331	genus.RikenellaceaeRC9gutgrou	TRUE	reported	RlaPhE	NA
rs2998141	C	T	-0.13635	0.029307	4.42E-06	3330	genus.RikenellaceaeRC9gutgrou	TRUE	reported	RlaPhE	NA
rs4717843	T	G	-0.11938	0.026055	4.72E-06	3220	genus.RikenellaceaeRC9gutgrou	TRUE	reported	RlaPhE	NA
rs9887954	A	G	-0.11485	0.024901	4.81E-06	3330	genus.RikenellaceaeRC9gutgrou	TRUE	reported	RlaPhE	NA
rs7193937	C	G	0.124153	0.027741	6.19E-06	3220	genus.RikenellaceaeRC9gutgrou	TRUE	reported	RlaPhE	NA
rs1159721	C	T	0.113569	0.025165	5.63E-06	3320	genus.RikenellaceaeRC9gutgrou	TRUE	reported	RlaPhE	NA
rs8030908	A	G	0.17405	0.038342	4.56E-06	3170	genus.RikenellaceaeRC9gutgrou	TRUE	reported	RlaPhE	NA
rs1250167	G	A	0.116395	0.026225	6.29E-06	3334	genus.RikenellaceaeRC9gutgrou	TRUE	reported	RlaPhE	NA
rs1703229	C	T	-0.16958	0.036825	6.61E-06	3206	genus.RikenellaceaeRC9gutgrou	TRUE	reported	RlaPhE	NA
rs1322394	C	T	-0.11742	0.026552	8.89E-06	3220	genus.RikenellaceaeRC9gutgrou	TRUE	reported	RlaPhE	NA
rs2074881	C	T	-0.14224	0.032391	9.45E-06	3315	genus.RikenellaceaeRC9gutgrou	TRUE	reported	RlaPhE	NA
rs7712231	G	A	0.156284	0.035046	7.97E-06	3334	genus.RikenellaceaeRC9gutgrou	TRUE	reported	RlaPhE	NA
rs6184150	A	G	0.092888	0.017145	4.00E-08	13568	genus.Romboutsia.id.11347.sum	TRUE	reported	sYNgLe	NA
rs1027997	G	A	-0.06222	0.012768	1.17E-06	14143	genus.Romboutsia.id.11347.sum	TRUE	reported	sYNgLe	NA
rs1684357	T	C	-0.08753	0.019678	5.08E-06	14186	genus.Romboutsia.id.11347.sum	TRUE	reported	sYNgLe	NA
rs7770269	G	A	-0.09441	0.020854	7.37E-06	12487	genus.Romboutsia.id.11347.sum	TRUE	reported	sYNgLe	NA
rs7598735	A	G	-0.12958	0.028045	6.71E-06	10466	genus.Romboutsia.id.11347.sum	TRUE	reported	sYNgLe	NA
rs7520053	G	T	-0.19064	0.042106	5.07E-06	5246	genus.Romboutsia.id.11347.sum	TRUE	reported	sYNgLe	NA
rs9567264	T	C	0.058002	0.012749	5.76E-06	14180	genus.Romboutsia.id.11347.sum	TRUE	reported	sYNgLe	NA
rs3430203	G	A	0.055026	0.012083	5.88E-06	14185	genus.Romboutsia.id.11347.sum	TRUE	reported	sYNgLe	NA
rs7109293	G	A	0.091911	0.020574	6.98E-06	14141	genus.Romboutsia.id.11347.sum	TRUE	reported	sYNgLe	NA
rs1009189	C	T	-0.06499	0.01455	6.62E-06	14188	genus.Romboutsia.id.11347.sum	TRUE	reported	sYNgLe	NA

rs1143987	C	G	-0.13104	0.029382	7.96E-06	10326	genus.Romboutsia.id.11347.sum	TRUE	reported	sYNgLe	NA
rs2860335	C	T	-0.21491	0.047474	8.52E-06	3693	genus.Romboutsia.id.11347.sum	TRUE	reported	sYNgLe	NA
rs1122142	C	T	-0.07268	0.015826	6.49E-06	14187	genus.Romboutsia.id.11347.sum	TRUE	reported	sYNgLe	NA
rs9389266	G	T	0.072281	0.016235	9.38E-06	14187	genus.Romboutsia.id.11347.sum	TRUE	reported	sYNgLe	NA
rs6250445	G	A	-0.071	0.015665	4.66E-06	14178	genus.Romboutsia.id.11347.sum	TRUE	reported	sYNgLe	NA
rs2034589	C	G	0.062976	0.012349	5.01E-07	17846	genus.Roseburia.id.2012.summa	TRUE	reported	OFvRu9	NA
rs1162705	A	T	-0.15384	0.032932	1.20E-06	9099	genus.Roseburia.id.2012.summa	TRUE	reported	OFvRu9	NA
rs2160994	C	T	0.055069	0.011248	9.70E-07	17444	genus.Roseburia.id.2012.summa	TRUE	reported	OFvRu9	NA
rs1691029	C	T	-0.09804	0.020957	2.91E-06	16648	genus.Roseburia.id.2012.summa	TRUE	reported	OFvRu9	NA
rs1274045	C	T	0.069753	0.015361	7.34E-06	17837	genus.Roseburia.id.2012.summa	TRUE	reported	OFvRu9	NA
rs6445851	A	G	-0.04973	0.010816	3.53E-06	17851	genus.Roseburia.id.2012.summa	TRUE	reported	OFvRu9	NA
rs9300744	T	C	-0.05885	0.012623	4.75E-06	17854	genus.Roseburia.id.2012.summa	TRUE	reported	OFvRu9	NA
rs2943022	C	T	0.049379	0.010676	4.11E-06	17854	genus.Roseburia.id.2012.summa	TRUE	reported	OFvRu9	NA
rs6930661	T	C	-0.09616	0.020497	2.48E-06	16945	genus.Roseburia.id.2012.summa	TRUE	reported	OFvRu9	NA
rs4748237	C	G	0.048833	0.01064	4.67E-06	17851	genus.Roseburia.id.2012.summa	TRUE	reported	OFvRu9	NA
rs1479900	G	A	-0.05789	0.01324	8.93E-06	17840	genus.Roseburia.id.2012.summa	TRUE	reported	OFvRu9	NA
rs329182	C	T	0.069033	0.015288	5.90E-06	17854	genus.Roseburia.id.2012.summa	TRUE	reported	OFvRu9	NA
rs302266	C	T	-0.07773	0.017299	8.13E-06	17056	genus.Roseburia.id.2012.summa	TRUE	reported	OFvRu9	NA
rs7532625	T	C	-0.10463	0.02309	7.50E-06	15893	genus.Roseburia.id.2012.summa	TRUE	reported	OFvRu9	NA
rs5585816	C	A	0.179284	0.040495	9.99E-06	5447	genus.Roseburia.id.2012.summa	TRUE	reported	OFvRu9	NA
rs5746617	T	C	0.074141	0.01716	8.30E-06	17333	genus.Roseburia.id.2012.summa	TRUE	reported	OFvRu9	NA
rs28040	C	G	0.056569	0.012679	9.26E-06	17854	genus.Roseburia.id.2012.summa	TRUE	reported	OFvRu9	NA
rs7875315	C	A	0.096874	0.021407	9.98E-06	16303	genus.Roseburia.id.2012.summa	TRUE	reported	OFvRu9	NA
rs243585	G	C	-0.05859	0.012102	1.33E-06	16906	genus.Ruminiclostridium5.id.1	TRUE	reported	WkQa1M	NA
rs2286384	C	G	-0.05188	0.010748	1.44E-06	17394	genus.Ruminiclostridium5.id.1	TRUE	reported	WkQa1M	NA
rs7996883	G	A	-0.09503	0.019351	1.15E-06	16602	genus.Ruminiclostridium5.id.1	TRUE	reported	WkQa1M	NA
rs2482038	A	C	0.051899	0.010878	1.70E-06	17396	genus.Ruminiclostridium5.id.1	TRUE	reported	WkQa1M	NA
rs1137539	C	T	0.082069	0.017446	3.99E-06	16680	genus.Ruminiclostridium5.id.1	TRUE	reported	WkQa1M	NA
rs1492620	C	T	-0.08305	0.018007	3.53E-06	17397	genus.Ruminiclostridium5.id.1	TRUE	reported	WkQa1M	NA
rs2791343	C	T	0.051717	0.011337	5.54E-06	17392	genus.Ruminiclostridium5.id.1	TRUE	reported	WkQa1M	NA
rs1082747	G	A	-0.05474	0.011516	2.19E-06	17391	genus.Ruminiclostridium5.id.1	TRUE	reported	WkQa1M	NA
rs6121460	A	G	0.093296	0.019921	2.64E-06	17318	genus.Ruminiclostridium5.id.1	TRUE	reported	WkQa1M	NA
rs2801960	G	C	0.052098	0.011511	6.21E-06	17387	genus.Ruminiclostridium5.id.1	TRUE	reported	WkQa1M	NA

rs8053158	G	A	-0.07405	0.015915	5.90E-06	17397	genus.Ruminiclostridium5.id.1	TRUE	reported	WkQa1M	NA
rs1223978	C	T	0.048417	0.010825	8.16E-06	17381	genus.Ruminiclostridium5.id.1	TRUE	reported	WkQa1M	NA
rs7300257	C	G	0.181548	0.041408	8.82E-06	3761	genus.Ruminiclostridium5.id.1	TRUE	reported	WkQa1M	NA
rs2833828	A	G	0.048962	0.01087	6.82E-06	17397	genus.Ruminiclostridium5.id.1	TRUE	reported	WkQa1M	NA
rs4955951	G	A	-0.07137	0.016583	9.96E-06	17397	genus.Ruminiclostridium5.id.1	TRUE	reported	WkQa1M	NA
rs1756364	T	G	0.099919	0.019717	2.54E-07	12933	genus.Ruminiclostridium6.id.1	TRUE	reported	JJEQXd	NA
rs7141412	G	T	0.200894	0.040643	1.08E-06	5567	genus.Ruminiclostridium6.id.1	TRUE	reported	JJEQXd	NA
rs6106092	G	T	0.15913	0.032228	1.09E-06	8955	genus.Ruminiclostridium6.id.1	TRUE	reported	JJEQXd	NA
rs7996817	A	G	0.11614	0.024308	1.66E-06	12802	genus.Ruminiclostridium6.id.1	TRUE	reported	JJEQXd	NA
rs7719351	G	A	0.073653	0.015316	1.30E-06	13380	genus.Ruminiclostridium6.id.1	TRUE	reported	JJEQXd	NA
rs1199218	C	A	0.06253	0.013788	4.65E-06	13381	genus.Ruminiclostridium6.id.1	TRUE	reported	JJEQXd	NA
rs7299153	G	T	0.135604	0.029517	4.95E-06	9642	genus.Ruminiclostridium6.id.1	TRUE	reported	JJEQXd	NA
rs663262	C	T	-0.13498	0.031071	3.39E-06	7876	genus.Ruminiclostridium6.id.1	TRUE	reported	JJEQXd	NA
rs9555756	C	A	-0.08041	0.017669	7.10E-06	13369	genus.Ruminiclostridium6.id.1	TRUE	reported	JJEQXd	NA
rs792058	A	G	0.055423	0.012542	8.58E-06	12933	genus.Ruminiclostridium6.id.1	TRUE	reported	JJEQXd	NA
rs1082982	C	T	-0.09761	0.021607	3.47E-06	12802	genus.Ruminiclostridium6.id.1	TRUE	reported	JJEQXd	NA
rs6747953	C	T	0.118994	0.026487	9.30E-06	11613	genus.Ruminiclostridium6.id.1	TRUE	reported	JJEQXd	NA
rs1871858	G	C	-0.10542	0.023727	9.12E-06	12818	genus.Ruminiclostridium6.id.1	TRUE	reported	JJEQXd	NA
rs1169695	G	A	-0.16682	0.037668	9.16E-06	6824	genus.Ruminiclostridium6.id.1	TRUE	reported	JJEQXd	NA
rs2548459	T	C	0.055487	0.012275	6.40E-06	13377	genus.Ruminiclostridium6.id.1	TRUE	reported	JJEQXd	NA
rs7317603	C	T	0.058665	0.013208	7.29E-06	13378	genus.Ruminiclostridium6.id.1	TRUE	reported	JJEQXd	NA
rs3536246	A	C	0.072008	0.016539	8.99E-06	13308	genus.Ruminiclostridium6.id.1	TRUE	reported	JJEQXd	NA
rs5766599	G	C	-0.06415	0.012334	2.07E-07	16725	genus.Ruminiclostridium9.id.1	TRUE	reported	QDekeH	NA
rs5587576	C	G	-0.05453	0.011297	1.60E-06	16725	genus.Ruminiclostridium9.id.1	TRUE	reported	QDekeH	NA
rs1204054	T	G	0.057042	0.012236	3.15E-06	16721	genus.Ruminiclostridium9.id.1	TRUE	reported	QDekeH	NA
rs1150445	A	G	-0.098	0.020262	2.37E-06	16107	genus.Ruminiclostridium9.id.1	TRUE	reported	QDekeH	NA
rs1241985	A	T	-0.0728	0.015559	3.18E-06	16027	genus.Ruminiclostridium9.id.1	TRUE	reported	QDekeH	NA
rs6082461	C	A	0.058642	0.0131	4.87E-06	16650	genus.Ruminiclostridium9.id.1	TRUE	reported	QDekeH	NA
rs1130487	G	C	0.060073	0.013274	4.10E-06	16719	genus.Ruminiclostridium9.id.1	TRUE	reported	QDekeH	NA
rs2615052	G	T	0.057761	0.012058	1.75E-06	16717	genus.Ruminiclostridium9.id.1	TRUE	reported	QDekeH	NA
rs918449	G	A	-0.09509	0.019718	2.56E-06	16725	genus.Ruminiclostridium9.id.1	TRUE	reported	QDekeH	NA
rs7359267	T	A	-0.08163	0.016951	2.14E-06	15493	genus.Ruminiclostridium9.id.1	TRUE	reported	QDekeH	NA
rs7908272	G	C	0.092858	0.020487	6.47E-06	16094	genus.Ruminiclostridium9.id.1	TRUE	reported	QDekeH	NA

rs7430317	C	T	0.053274	0.011927	7.92E-06	16706	genus.Ruminiclostridium9.id.1	TRUE	reported	QDekeH	NA
rs1303331	A	T	0.051119	0.011142	5.68E-06	16724	genus.Ruminiclostridium9.id.1	TRUE	reported	QDekeH	NA
rs9809789	T	C	-0.07175	0.015967	8.72E-06	16025	genus.Ruminiclostridium9.id.1	TRUE	reported	QDekeH	NA
rs9522712	C	T	0.069973	0.015494	4.66E-06	16725	genus.Ruminiclostridium9.id.1	TRUE	reported	QDekeH	NA
rs7819172	C	T	0.0945	0.021021	7.58E-06	16039	genus.Ruminiclostridium9.id.1	TRUE	reported	QDekeH	NA
rs7137760	T	C	0.050797	0.011218	7.07E-06	16722	genus.Ruminiclostridium9.id.1	TRUE	reported	QDekeH	NA
rs5994253	G	A	-0.08113	0.015762	2.35E-07	15288	genus.RuminococcaceaeNK4A214g	TRUE	reported	kxTLyy	NA
rs1170469	T	G	-0.08997	0.018475	8.71E-07	15206	genus.RuminococcaceaeNK4A214g	TRUE	reported	kxTLyy	NA
rs136761	A	G	-0.05875	0.011915	8.15E-07	14822	genus.RuminococcaceaeNK4A214g	TRUE	reported	kxTLyy	NA
rs1158641	A	G	-0.08634	0.016994	3.66E-07	14822	genus.RuminococcaceaeNK4A214g	TRUE	reported	kxTLyy	NA
rs3457693	C	G	-0.08733	0.019489	4.72E-06	14678	genus.RuminococcaceaeNK4A214g	TRUE	reported	kxTLyy	NA
rs1017877	T	G	0.071439	0.016212	7.72E-06	15268	genus.RuminococcaceaeNK4A214g	TRUE	reported	kxTLyy	NA
rs1142444	G	C	-0.17527	0.037278	3.59E-06	7123	genus.RuminococcaceaeNK4A214g	TRUE	reported	kxTLyy	NA
rs4814689	T	C	-0.1083	0.023071	4.55E-06	14009	genus.RuminococcaceaeNK4A214g	TRUE	reported	kxTLyy	NA
rs7315881	G	C	-0.10926	0.022746	2.20E-06	13077	genus.RuminococcaceaeNK4A214g	TRUE	reported	kxTLyy	NA
rs12731	G	A	-0.05277	0.011505	4.87E-06	15288	genus.RuminococcaceaeNK4A214g	TRUE	reported	kxTLyy	NA
rs7573569	C	T	0.107739	0.023364	3.23E-06	14822	genus.RuminococcaceaeNK4A214g	TRUE	reported	kxTLyy	NA
rs1474751	G	A	-0.13377	0.02952	4.72E-06	9917	genus.RuminococcaceaeNK4A214g	TRUE	reported	kxTLyy	NA
rs6202736	C	T	0.061539	0.013761	6.58E-06	14822	genus.RuminococcaceaeNK4A214g	TRUE	reported	kxTLyy	NA
rs1264203	C	T	-0.0553	0.01194	3.43E-06	15276	genus.RuminococcaceaeNK4A214g	TRUE	reported	kxTLyy	NA
rs3555991	C	T	-0.09251	0.020367	4.89E-06	13976	genus.RuminococcaceaeNK4A214g	TRUE	reported	kxTLyy	NA
rs1308769	G	T	0.057462	0.012594	8.69E-06	15288	genus.RuminococcaceaeNK4A214g	TRUE	reported	kxTLyy	NA
rs6681678	T	C	-0.10017	0.024	9.05E-06	13031	genus.RuminococcaceaeNK4A214g	TRUE	reported	kxTLyy	NA
rs1124174	T	C	0.053386	0.012003	6.59E-06	14822	genus.RuminococcaceaeNK4A214g	TRUE	reported	kxTLyy	NA
rs7756431	C	A	-0.07131	0.014086	3.29E-07	17000	genus.RuminococcaceaeUCG002.i	TRUE	reported	1TjP9u	NA
rs5579312	C	T	0.137396	0.027414	4.81E-07	11669	genus.RuminococcaceaeUCG002.i	TRUE	reported	1TjP9u	NA
rs1092742	A	C	-0.07136	0.014771	8.50E-07	17096	genus.RuminococcaceaeUCG002.i	TRUE	reported	1TjP9u	NA
rs6774692	G	C	-0.05424	0.011036	9.17E-07	16651	genus.RuminococcaceaeUCG002.i	TRUE	reported	1TjP9u	NA
rs1169748	A	C	-0.18973	0.039657	2.03E-06	5819	genus.RuminococcaceaeUCG002.i	TRUE	reported	1TjP9u	NA
rs7155595	A	C	0.056993	0.011699	1.15E-06	17084	genus.RuminococcaceaeUCG002.i	TRUE	reported	1TjP9u	NA
rs7120052	C	A	0.06248	0.013552	1.97E-06	17097	genus.RuminococcaceaeUCG002.i	TRUE	reported	1TjP9u	NA
rs1091613	T	C	-0.06933	0.014675	2.87E-06	17097	genus.RuminococcaceaeUCG002.i	TRUE	reported	1TjP9u	NA
rs7901605	T	C	-0.08877	0.018942	2.34E-06	16937	genus.RuminococcaceaeUCG002.i	TRUE	reported	1TjP9u	NA

rs1246337	G	A	-0.05221	0.011209	2.96E-06	16994	genus.RuminococcaceaeUCG002.i	TRUE	reported	1TjP9u	NA
rs1175029	T	G	-0.05783	0.012051	1.76E-06	17094	genus.RuminococcaceaeUCG002.i	TRUE	reported	1TjP9u	NA
rs2265670	G	C	-0.05124	0.010929	2.99E-06	17096	genus.RuminococcaceaeUCG002.i	TRUE	reported	1TjP9u	NA
rs15256	T	C	0.073238	0.016834	9.46E-06	17095	genus.RuminococcaceaeUCG002.i	TRUE	reported	1TjP9u	NA
rs882348	G	A	-0.07999	0.017862	5.45E-06	17097	genus.RuminococcaceaeUCG002.i	TRUE	reported	1TjP9u	NA
rs7287419	C	G	-0.07703	0.016725	3.47E-06	16651	genus.RuminococcaceaeUCG002.i	TRUE	reported	1TjP9u	NA
rs6793778	T	C	-0.05587	0.012526	9.81E-06	17094	genus.RuminococcaceaeUCG002.i	TRUE	reported	1TjP9u	NA
rs7342369	A	C	-0.05278	0.011602	5.66E-06	16651	genus.RuminococcaceaeUCG002.i	TRUE	reported	1TjP9u	NA
rs1096444	A	G	-0.14906	0.034486	7.45E-06	5346	genus.RuminococcaceaeUCG002.i	TRUE	reported	1TjP9u	NA
rs1160747	G	A	-0.07802	0.017632	7.19E-06	16282	genus.RuminococcaceaeUCG002.i	TRUE	reported	1TjP9u	NA
rs7249614	G	A	-0.04928	0.011081	9.07E-06	16651	genus.RuminococcaceaeUCG002.i	TRUE	reported	1TjP9u	NA
rs7684726	G	A	0.163508	0.035615	5.17E-06	7654	genus.RuminococcaceaeUCG002.i	TRUE	reported	1TjP9u	NA
rs6542556	G	A	0.050974	0.011406	7.86E-06	17094	genus.RuminococcaceaeUCG002.i	TRUE	reported	1TjP9u	NA
rs5707934	G	T	-0.07657	0.017282	7.22E-06	16372	genus.RuminococcaceaeUCG002.i	TRUE	reported	1TjP9u	NA
rs5603042	A	G	-0.09829	0.021634	6.30E-06	14937	genus.RuminococcaceaeUCG002.i	TRUE	reported	1TjP9u	NA
rs362417	C	G	-0.05486	0.012098	7.80E-06	17097	genus.RuminococcaceaeUCG002.i	TRUE	reported	1TjP9u	NA
rs1131473	G	A	-0.07584	0.016456	7.69E-06	16370	genus.RuminococcaceaeUCG002.i	TRUE	reported	1TjP9u	NA
rs7334154	C	T	-0.16986	0.031877	1.51E-07	6744	genus.RuminococcaceaeUCG003.i	TRUE	reported	uTaAn9	NA
rs6759615	G	A	0.102523	0.02002	7.86E-07	14410	genus.RuminococcaceaeUCG003.i	TRUE	reported	uTaAn9	NA
rs646327	A	G	0.05867	0.011837	7.83E-07	14406	genus.RuminococcaceaeUCG003.i	TRUE	reported	uTaAn9	NA
rs1161391	T	G	0.07276	0.015572	1.63E-06	14309	genus.RuminococcaceaeUCG003.i	TRUE	reported	uTaAn9	NA
rs1124341	C	T	-0.09257	0.01912	1.67E-06	14004	genus.RuminococcaceaeUCG003.i	TRUE	reported	uTaAn9	NA
rs1695979	C	A	-0.06253	0.013126	2.22E-06	14412	genus.RuminococcaceaeUCG003.i	TRUE	reported	uTaAn9	NA
rs4452755	C	A	-0.06345	0.013474	3.29E-06	14406	genus.RuminococcaceaeUCG003.i	TRUE	reported	uTaAn9	NA
rs1049028	T	C	-0.06721	0.014331	4.16E-06	14004	genus.RuminococcaceaeUCG003.i	TRUE	reported	uTaAn9	NA
rs4532474	A	G	0.076923	0.017045	4.82E-06	14412	genus.RuminococcaceaeUCG003.i	TRUE	reported	uTaAn9	NA
rs4629039	A	T	0.055374	0.012266	6.54E-06	14404	genus.RuminococcaceaeUCG003.i	TRUE	reported	uTaAn9	NA
rs2523124	C	T	-0.05466	0.012082	5.78E-06	14409	genus.RuminococcaceaeUCG003.i	TRUE	reported	uTaAn9	NA
rs3013089	A	G	-0.05515	0.012035	4.38E-06	14408	genus.RuminococcaceaeUCG003.i	TRUE	reported	uTaAn9	NA
rs7872011	G	A	-0.11535	0.02496	7.59E-06	13078	genus.RuminococcaceaeUCG003.i	TRUE	reported	uTaAn9	NA
rs139730	C	G	-0.05777	0.013064	9.72E-06	14004	genus.RuminococcaceaeUCG003.i	TRUE	reported	uTaAn9	NA
rs6769553	G	A	0.084974	0.015745	7.91E-08	9501	genus.RuminococcaceaeUCG004.i	TRUE	reported	6Bpx50	NA
rs1212573	T	G	0.133972	0.025742	2.09E-07	8938	genus.RuminococcaceaeUCG004.i	TRUE	reported	6Bpx50	NA

rs511258	A	G	-0.07575	0.016247	4.52E-06	9500	genus.RuminococcaceaeUCG004.i	TRUE	reported	6Bpx50	NA
rs2248146	C	T	0.068945	0.015365	8.20E-06	9502	genus.RuminococcaceaeUCG004.i	TRUE	reported	6Bpx50	NA
rs1097622	G	T	0.095976	0.021455	7.04E-06	9116	genus.RuminococcaceaeUCG004.i	TRUE	reported	6Bpx50	NA
rs7569771	G	A	-0.07591	0.017031	8.12E-06	9502	genus.RuminococcaceaeUCG004.i	TRUE	reported	6Bpx50	NA
rs872501	A	G	0.116125	0.025946	5.81E-06	8473	genus.RuminococcaceaeUCG004.i	TRUE	reported	6Bpx50	NA
rs550351	C	A	0.078587	0.018003	9.43E-06	9498	genus.RuminococcaceaeUCG004.i	TRUE	reported	6Bpx50	NA
rs3800154	C	A	-0.0798	0.017764	6.12E-06	9406	genus.RuminococcaceaeUCG004.i	TRUE	reported	6Bpx50	NA
rs1196189	A	G	-0.07078	0.016138	9.18E-06	9501	genus.RuminococcaceaeUCG004.i	TRUE	reported	6Bpx50	NA
rs9818949	T	G	0.085993	0.018867	5.39E-06	9116	genus.RuminococcaceaeUCG004.i	TRUE	reported	6Bpx50	NA
rs7123615	G	C	-0.07862	0.017959	7.09E-06	9502	genus.RuminococcaceaeUCG004.i	TRUE	reported	6Bpx50	NA
rs1095069	C	T	0.057763	0.011412	4.30E-07	15821	genus.RuminococcaceaeUCG005.i	TRUE	reported	Ytco24	NA
rs3478134	A	G	0.188685	0.038648	6.05E-07	6061	genus.RuminococcaceaeUCG005.i	TRUE	reported	Ytco24	NA
rs1228851	G	A	0.066635	0.014436	3.10E-06	15473	genus.RuminococcaceaeUCG005.i	TRUE	reported	Ytco24	NA
rs6008166	G	C	0.158082	0.031959	9.28E-07	9342	genus.RuminococcaceaeUCG005.i	TRUE	reported	Ytco24	NA
rs1142795	G	A	-0.1466	0.031603	3.22E-06	8465	genus.RuminococcaceaeUCG005.i	TRUE	reported	Ytco24	NA
rs1245821	C	T	0.067732	0.014452	2.41E-06	16099	genus.RuminococcaceaeUCG005.i	TRUE	reported	Ytco24	NA
rs3516612	G	C	-0.06862	0.014625	3.75E-06	16092	genus.RuminococcaceaeUCG005.i	TRUE	reported	Ytco24	NA
rs7555878	G	A	0.058668	0.012528	2.81E-06	16100	genus.RuminococcaceaeUCG005.i	TRUE	reported	Ytco24	NA
rs394449	T	A	0.069291	0.014871	2.60E-06	15728	genus.RuminococcaceaeUCG005.i	TRUE	reported	Ytco24	NA
rs1087344	C	T	0.065484	0.014397	4.11E-06	16098	genus.RuminococcaceaeUCG005.i	TRUE	reported	Ytco24	NA
rs2893871	A	G	-0.07364	0.015548	3.54E-06	16103	genus.RuminococcaceaeUCG005.i	TRUE	reported	Ytco24	NA
rs7277657	A	C	0.087067	0.019718	5.36E-06	15376	genus.RuminococcaceaeUCG005.i	TRUE	reported	Ytco24	NA
rs898577	C	T	-0.12301	0.028667	7.46E-06	9991	genus.RuminococcaceaeUCG005.i	TRUE	reported	Ytco24	NA
rs7449320	A	C	0.059916	0.013084	4.81E-06	16095	genus.RuminococcaceaeUCG005.i	TRUE	reported	Ytco24	NA
rs1093780	A	G	0.075581	0.016823	8.17E-06	15821	genus.RuminococcaceaeUCG005.i	TRUE	reported	Ytco24	NA
rs7586445	A	G	0.078235	0.017646	8.81E-06	15099	genus.RuminococcaceaeUCG005.i	TRUE	reported	Ytco24	NA
rs5579312	C	T	0.121548	0.027955	7.37E-06	11224	genus.RuminococcaceaeUCG005.i	TRUE	reported	Ytco24	NA
rs8009993	C	G	-0.13594	0.024486	4.42E-08	7389	genus.RuminococcaceaeUCG009.i	TRUE	reported	e18Afb	NA
rs1550196	A	G	0.130842	0.026248	1.13E-06	7389	genus.RuminococcaceaeUCG009.i	TRUE	reported	e18Afb	NA
rs4708333	G	T	-0.08403	0.017476	1.56E-06	7511	genus.RuminococcaceaeUCG009.i	TRUE	reported	e18Afb	NA
rs6177933	C	G	-0.13814	0.029207	1.94E-06	6865	genus.RuminococcaceaeUCG009.i	TRUE	reported	e18Afb	NA
rs4079028	T	C	0.091562	0.019936	3.28E-06	7480	genus.RuminococcaceaeUCG009.i	TRUE	reported	e18Afb	NA
rs2058609	G	A	0.081646	0.017472	3.12E-06	7510	genus.RuminococcaceaeUCG009.i	TRUE	reported	e18Afb	NA

rs1250821	T	C	-0.07746	0.016894	4.75E-06	7511	genus.RuminococcaceaeUCG009.i	TRUE	reported	e18Afb	NA
rs9558661	C	T	-0.08978	0.020074	7.01E-06	7480	genus.RuminococcaceaeUCG009.i	TRUE	reported	e18Afb	NA
rs758191	G	T	0.17702	0.037511	9.01E-06	5829	genus.RuminococcaceaeUCG009.i	TRUE	reported	e18Afb	NA
rs2192926	G	A	-0.08905	0.019299	4.88E-06	7511	genus.RuminococcaceaeUCG009.i	TRUE	reported	e18Afb	NA
rs1130068	C	T	-0.09289	0.020803	7.98E-06	7165	genus.RuminococcaceaeUCG009.i	TRUE	reported	e18Afb	NA
rs1384606	G	A	0.139274	0.031579	9.81E-06	6865	genus.RuminococcaceaeUCG009.i	TRUE	reported	e18Afb	NA
rs6952765	A	G	0.07321	0.016678	8.13E-06	7511	genus.RuminococcaceaeUCG009.i	TRUE	reported	e18Afb	NA
rs7841064	G	A	0.120989	0.027732	9.67E-06	7510	genus.RuminococcaceaeUCG009.i	TRUE	reported	e18Afb	NA
rs3550691	C	G	-0.06937	0.014793	3.21E-06	12793	genus.RuminococcaceaeUCG010.i	TRUE	reported	axLZ59	NA
rs682403	G	A	-0.05882	0.012467	2.37E-06	12864	genus.RuminococcaceaeUCG010.i	TRUE	reported	axLZ59	NA
rs6958419	T	C	-0.05857	0.012499	2.84E-06	12870	genus.RuminococcaceaeUCG010.i	TRUE	reported	axLZ59	NA
rs2820282	C	A	-0.05923	0.012592	2.85E-06	12865	genus.RuminococcaceaeUCG010.i	TRUE	reported	axLZ59	NA
rs1259710	A	G	0.067085	0.014441	4.87E-06	12415	genus.RuminococcaceaeUCG010.i	TRUE	reported	axLZ59	NA
rs7935775	T	A	-0.06314	0.013795	4.99E-06	12864	genus.RuminococcaceaeUCG010.i	TRUE	reported	axLZ59	NA
rs7321880	A	G	-0.16621	0.036794	6.43E-06	6714	genus.RuminococcaceaeUCG010.i	TRUE	reported	axLZ59	NA
rs7441445	T	C	-0.05695	0.01265	6.80E-06	12852	genus.RuminococcaceaeUCG010.i	TRUE	reported	axLZ59	NA
rs1416041	C	A	-0.18234	0.03399	7.04E-08	3632	genus.RuminococcaceaeUCG011.i	TRUE	reported	VDQoMG	NA
rs1272432	T	C	-0.12088	0.024923	1.52E-06	3632	genus.RuminococcaceaeUCG011.i	TRUE	reported	VDQoMG	NA
rs7911308	T	C	-0.15217	0.031752	2.06E-06	3610	genus.RuminococcaceaeUCG011.i	TRUE	reported	VDQoMG	NA
rs9729514	G	A	0.184934	0.039462	2.37E-06	3632	genus.RuminococcaceaeUCG011.i	TRUE	reported	VDQoMG	NA
rs2729556	T	C	-0.1091	0.02337	3.19E-06	3632	genus.RuminococcaceaeUCG011.i	TRUE	reported	VDQoMG	NA
rs1027456	T	C	0.110917	0.024456	6.50E-06	3632	genus.RuminococcaceaeUCG011.i	TRUE	reported	VDQoMG	NA
rs4490371	C	T	-0.11182	0.024896	7.75E-06	3610	genus.RuminococcaceaeUCG011.i	TRUE	reported	VDQoMG	NA
rs1263631	A	G	0.132725	0.028204	2.81E-06	3632	genus.RuminococcaceaeUCG011.i	TRUE	reported	VDQoMG	NA
rs1278171	T	C	-0.06561	0.011748	2.55E-08	16772	genus.RuminococcaceaeUCG013.i	TRUE	reported	yGkj1A	NA
rs1218934	A	G	0.068496	0.014558	1.68E-06	16470	genus.RuminococcaceaeUCG013.i	TRUE	reported	yGkj1A	NA
rs7508894	C	T	-0.0943	0.020071	2.55E-06	15638	genus.RuminococcaceaeUCG013.i	TRUE	reported	yGkj1A	NA
rs1691886	C	A	0.111491	0.024016	4.16E-06	12643	genus.RuminococcaceaeUCG013.i	TRUE	reported	yGkj1A	NA
rs7697348	T	G	0.194976	0.041821	3.35E-06	5299	genus.RuminococcaceaeUCG013.i	TRUE	reported	yGkj1A	NA
rs1248535	A	G	-0.06079	0.013085	4.19E-06	16771	genus.RuminococcaceaeUCG013.i	TRUE	reported	yGkj1A	NA
rs9565219	A	T	-0.05246	0.01177	8.73E-06	16772	genus.RuminococcaceaeUCG013.i	TRUE	reported	yGkj1A	NA
rs1729065	C	G	-0.05334	0.012076	9.64E-06	16470	genus.RuminococcaceaeUCG013.i	TRUE	reported	yGkj1A	NA
rs7784330	A	G	-0.04983	0.011207	8.16E-06	16770	genus.RuminococcaceaeUCG013.i	TRUE	reported	yGkj1A	NA

rs1158188	T	C	0.066121	0.014474	4.73E-06	15730	genus.RuminococcaceaeUCG013.i	TRUE	reported	yGkj1A	NA
rs2428106	G	C	-0.04909	0.01099	8.38E-06	16768	genus.RuminococcaceaeUCG013.i	TRUE	reported	yGkj1A	NA
rs2730183	A	G	-0.04887	0.010991	8.44E-06	16772	genus.RuminococcaceaeUCG013.i	TRUE	reported	yGkj1A	NA
rs9313055	C	T	0.105087	0.023446	9.55E-06	15764	genus.RuminococcaceaeUCG013.i	TRUE	reported	yGkj1A	NA
rs1233678	C	T	-0.0856	0.018931	8.60E-06	16772	genus.RuminococcaceaeUCG013.i	TRUE	reported	yGkj1A	NA
rs4385846	T	G	0.05984	0.013181	6.46E-06	16772	genus.RuminococcaceaeUCG013.i	TRUE	reported	yGkj1A	NA
rs1157778	C	T	-0.18835	0.038664	4.62E-07	5674	genus.RuminococcaceaeUCG014.i	TRUE	reported	x0JLtT	NA
rs7280922	C	T	0.067178	0.013984	2.41E-06	13922	genus.RuminococcaceaeUCG014.i	TRUE	reported	x0JLtT	NA
rs1263813	G	T	0.058255	0.011966	1.21E-06	14403	genus.RuminococcaceaeUCG014.i	TRUE	reported	x0JLtT	NA
rs5610523	A	G	0.139276	0.029913	2.91E-06	9190	genus.RuminococcaceaeUCG014.i	TRUE	reported	x0JLtT	NA
rs995642	T	C	0.060048	0.012642	1.90E-06	14402	genus.RuminococcaceaeUCG014.i	TRUE	reported	x0JLtT	NA
rs1094129	T	C	-0.12206	0.026002	2.40E-06	12615	genus.RuminococcaceaeUCG014.i	TRUE	reported	x0JLtT	NA
rs7964038	A	T	-0.11086	0.02481	8.74E-06	13692	genus.RuminococcaceaeUCG014.i	TRUE	reported	x0JLtT	NA
rs439810	C	G	-0.05771	0.012668	7.04E-06	14403	genus.RuminococcaceaeUCG014.i	TRUE	reported	x0JLtT	NA
rs1049539	T	C	-0.08249	0.018719	9.96E-06	13902	genus.RuminococcaceaeUCG014.i	TRUE	reported	x0JLtT	NA
rs6247883	A	T	-0.05813	0.012903	6.04E-06	14331	genus.RuminococcaceaeUCG014.i	TRUE	reported	x0JLtT	NA
rs6189881	T	A	0.060786	0.013876	9.92E-06	14325	genus.RuminococcaceaeUCG014.i	TRUE	reported	x0JLtT	NA
rs853612	G	A	-0.05283	0.011937	9.75E-06	14403	genus.RuminococcaceaeUCG014.i	TRUE	reported	x0JLtT	NA
rs7318622	A	G	-0.09939	0.021682	6.72E-06	13760	genus.RuminococcaceaeUCG014.i	TRUE	reported	x0JLtT	NA
rs3440207	T	C	-0.0688	0.015608	9.80E-06	13863	genus.RuminococcaceaeUCG014.i	TRUE	reported	x0JLtT	NA
rs7406014	G	C	-0.11577	0.02542	8.71E-06	13011	genus.RuminococcaceaeUCG014.i	TRUE	reported	x0JLtT	NA
rs7762708	G	C	0.067562	0.01501	7.43E-06	14390	genus.RuminococcaceaeUCG014.i	TRUE	reported	x0JLtT	NA
rs1729693	G	C	-0.08295	0.018506	7.34E-06	13177	genus.RuminococcaceaeUCG014.i	TRUE	reported	x0JLtT	NA
rs1079116	G	A	-0.06646	0.015007	9.76E-06	14403	genus.RuminococcaceaeUCG014.i	TRUE	reported	x0JLtT	NA
rs1076915	C	G	-0.06403	0.011006	5.29E-09	16603	genus.Ruminococcus1.id.11373.	TRUE	reported	GVF4EV	NA
rs7117576	G	A	0.08295	0.017089	6.48E-07	16606	genus.Ruminococcus1.id.11373.	TRUE	reported	GVF4EV	NA
rs1778186	T	C	0.099927	0.021173	1.96E-06	14349	genus.Ruminococcus1.id.11373.	TRUE	reported	GVF4EV	NA
rs7583465	T	C	0.052765	0.011262	2.56E-06	16588	genus.Ruminococcus1.id.11373.	TRUE	reported	GVF4EV	NA
rs7861352	A	G	0.167497	0.036764	5.11E-06	7266	genus.Ruminococcus1.id.11373.	TRUE	reported	GVF4EV	NA
rs7857213	A	G	0.125038	0.027941	5.23E-06	11667	genus.Ruminococcus1.id.11373.	TRUE	reported	GVF4EV	NA
rs3000856	A	T	-0.071	0.016277	9.28E-06	16126	genus.Ruminococcus1.id.11373.	TRUE	reported	GVF4EV	NA
rs6493760	T	C	0.053526	0.011588	3.38E-06	16126	genus.Ruminococcus1.id.11373.	TRUE	reported	GVF4EV	NA
rs1178369	T	G	-0.07341	0.01614	4.73E-06	16606	genus.Ruminococcus1.id.11373.	TRUE	reported	GVF4EV	NA

rs1099581	G	C	-0.0762	0.017469	8.38E-06	16597	genus.Ruminococcus1.id.11373.	TRUE	reported	GVF4EV	NA
rs1016783	G	A	0.051951	0.011631	8.09E-06	16605	genus.Ruminococcus1.id.11373.	TRUE	reported	GVF4EV	NA
rs3819978	T	C	-0.11504	0.026014	8.74E-06	12769	genus.Ruminococcus1.id.11373.	TRUE	reported	GVF4EV	NA
rs4849717	A	T	0.132764	0.029982	8.83E-06	10484	genus.Ruminococcus1.id.11373.	TRUE	reported	GVF4EV	NA
rs6105066	C	T	-0.06071	0.013425	5.06E-06	16049	genus.Ruminococcus1.id.11373.	TRUE	reported	GVF4EV	NA
rs7812038	G	A	-0.19282	0.039205	3.31E-07	6082	genus.Ruminococcus2.id.11374.	TRUE	reported	zXOGuW	NA
rs1298662	T	C	0.066594	0.014017	2.14E-06	14714	genus.Ruminococcus2.id.11374.	TRUE	reported	zXOGuW	NA
rs7635831	A	G	0.061834	0.012903	1.98E-06	14924	genus.Ruminococcus2.id.11374.	TRUE	reported	zXOGuW	NA
rs2997412	G	A	-0.05684	0.012232	4.22E-06	15336	genus.Ruminococcus2.id.11374.	TRUE	reported	zXOGuW	NA
rs2368224	G	T	0.199648	0.043845	3.63E-06	3026	genus.Ruminococcus2.id.11374.	TRUE	reported	zXOGuW	NA
rs4400279	G	A	0.054611	0.012008	5.80E-06	15333	genus.Ruminococcus2.id.11374.	TRUE	reported	zXOGuW	NA
rs5868173	G	A	0.072388	0.016079	4.18E-06	15338	genus.Ruminococcus2.id.11374.	TRUE	reported	zXOGuW	NA
rs1819812	T	G	0.084236	0.018489	5.28E-06	14868	genus.Ruminococcus2.id.11374.	TRUE	reported	zXOGuW	NA
rs1240630	C	A	-0.06319	0.014228	9.79E-06	15339	genus.Ruminococcus2.id.11374.	TRUE	reported	zXOGuW	NA
rs2846589	T	G	0.052209	0.011638	7.59E-06	14924	genus.Ruminococcus2.id.11374.	TRUE	reported	zXOGuW	NA
rs5570711	A	C	0.086552	0.018914	8.01E-06	15336	genus.Ruminococcus2.id.11374.	TRUE	reported	zXOGuW	NA
rs7514080	G	T	0.083682	0.017645	3.95E-06	14249	genus.Ruminococcus2.id.11374.	TRUE	reported	zXOGuW	NA
rs4799823	T	C	0.0837	0.018226	5.40E-06	14650	genus.Ruminococcus2.id.11374.	TRUE	reported	zXOGuW	NA
rs6179156	C	T	-0.05235	0.011712	6.79E-06	15319	genus.Ruminococcus2.id.11374.	TRUE	reported	zXOGuW	NA
rs7693984	A	G	-0.10282	0.023509	9.42E-06	14154	genus.Ruminococcus2.id.11374.	TRUE	reported	zXOGuW	NA
rs1341718	C	T	0.166509	0.033752	7.62E-07	3047	genus.Sellimonas.id.14369.sum	TRUE	reported	9JUUdl	NA
rs2371572	C	A	0.127349	0.025086	4.46E-07	3154	genus.Sellimonas.id.14369.sum	TRUE	reported	9JUUdl	NA
rs6201646	C	T	0.233969	0.048428	1.09E-06	3153	genus.Sellimonas.id.14369.sum	TRUE	reported	9JUUdl	NA
rs1133790	C	T	-0.16276	0.035702	7.21E-06	3154	genus.Sellimonas.id.14369.sum	TRUE	reported	9JUUdl	NA
rs2187447	C	A	0.243457	0.05277	3.98E-06	3028	genus.Sellimonas.id.14369.sum	TRUE	reported	9JUUdl	NA
rs2016057	C	A	-0.12588	0.025615	1.03E-06	3154	genus.Sellimonas.id.14369.sum	TRUE	reported	9JUUdl	NA
rs7255385	C	G	-0.15046	0.033263	5.38E-06	3154	genus.Sellimonas.id.14369.sum	TRUE	reported	9JUUdl	NA
rs5620327	C	T	-0.12405	0.026911	3.72E-06	3154	genus.Sellimonas.id.14369.sum	TRUE	reported	9JUUdl	NA
rs553697	C	T	-0.15377	0.03391	6.13E-06	3047	genus.Sellimonas.id.14369.sum	TRUE	reported	9JUUdl	NA
rs6433790	T	C	-0.13853	0.03017	3.98E-06	3095	genus.Sellimonas.id.14369.sum	TRUE	reported	9JUUdl	NA
rs7968030	T	A	-0.12711	0.028062	5.56E-06	3095	genus.Sellimonas.id.14369.sum	TRUE	reported	9JUUdl	NA
rs1765732	T	C	0.114571	0.025362	8.25E-06	3154	genus.Sellimonas.id.14369.sum	TRUE	reported	9JUUdl	NA
rs41816	G	A	0.132201	0.029109	8.39E-06	3154	genus.Sellimonas.id.14369.sum	TRUE	reported	9JUUdl	NA

rs11787822	A	C	0.081327	0.017115	2.63E-06	6923	genus.Senegalimassilia.id.111	TRUE	reported	s9vAcp	NA
rs7288780	A	T	-0.08225	0.017572	2.42E-06	6923	genus.Senegalimassilia.id.111	TRUE	reported	s9vAcp	NA
rs7225245	A	G	0.079173	0.017042	4.18E-06	6923	genus.Senegalimassilia.id.111	TRUE	reported	s9vAcp	NA
rs5751250	A	T	0.081949	0.017224	2.03E-06	6923	genus.Senegalimassilia.id.111	TRUE	reported	s9vAcp	NA
rs1003690	T	C	0.185519	0.040088	8.05E-06	4996	genus.Senegalimassilia.id.111	TRUE	reported	s9vAcp	NA
rs1338327	C	G	0.077468	0.017084	6.04E-06	6923	genus.Senegalimassilia.id.111	TRUE	reported	s9vAcp	NA
rs1990708	C	A	-0.10962	0.024779	8.91E-06	6923	genus.Senegalimassilia.id.111	TRUE	reported	s9vAcp	NA
rs2017373	T	C	0.078226	0.017684	9.50E-06	6923	genus.Senegalimassilia.id.111	TRUE	reported	s9vAcp	NA
rs8901	T	C	0.093459	0.018681	6.07E-07	6063	genus.Slackia.id.825.summary.	TRUE	reported	0JaP4u	NA
rs4492265	G	A	-0.09058	0.019166	2.41E-06	6063	genus.Slackia.id.825.summary.	TRUE	reported	0JaP4u	NA
rs1689413	T	C	-0.12279	0.026305	2.71E-06	6063	genus.Slackia.id.825.summary.	TRUE	reported	0JaP4u	NA
rs1127642	A	T	0.194714	0.04116	3.40E-06	5634	genus.Slackia.id.825.summary.	TRUE	reported	0JaP4u	NA
rs1244044	G	A	0.090193	0.019058	2.63E-06	6063	genus.Slackia.id.825.summary.	TRUE	reported	0JaP4u	NA
rs3515698	C	T	-0.15571	0.034808	8.06E-06	5965	genus.Slackia.id.825.summary.	TRUE	reported	0JaP4u	NA
rs1040978	G	A	0.095083	0.021124	7.70E-06	5941	genus.Slackia.id.825.summary.	TRUE	reported	0JaP4u	NA
rs1333923	G	C	0.147055	0.033064	7.42E-06	6063	genus.Slackia.id.825.summary.	TRUE	reported	0JaP4u	NA
rs5876732	C	G	-0.10283	0.022708	4.60E-06	6063	genus.Slackia.id.825.summary.	TRUE	reported	0JaP4u	NA
rs1111028	C	T	-0.13752	0.02274	2.58E-09	15120	genus.Streptococcus.id.1853.s	TRUE	reported	wZL5zu	NA
rs4968759	G	A	-0.05151	0.011207	3.78E-06	16384	genus.Streptococcus.id.1853.s	TRUE	reported	wZL5zu	NA
rs1176438	G	A	-0.06953	0.014367	1.29E-06	16387	genus.Streptococcus.id.1853.s	TRUE	reported	wZL5zu	NA
rs7273963	G	A	0.095994	0.019321	1.03E-06	14920	genus.Streptococcus.id.1853.s	TRUE	reported	wZL5zu	NA
rs1918540	A	G	0.059639	0.012815	2.44E-06	16373	genus.Streptococcus.id.1853.s	TRUE	reported	wZL5zu	NA
rs1770827	G	A	-0.0794	0.017063	3.04E-06	16278	genus.Streptococcus.id.1853.s	TRUE	reported	wZL5zu	NA
rs7755851	G	A	-0.104	0.022971	4.71E-06	14949	genus.Streptococcus.id.1853.s	TRUE	reported	wZL5zu	NA
rs1172039	A	G	0.107024	0.022812	3.59E-06	15220	genus.Streptococcus.id.1853.s	TRUE	reported	wZL5zu	NA
rs395407	C	G	-0.07928	0.01737	4.37E-06	15864	genus.Streptococcus.id.1853.s	TRUE	reported	wZL5zu	NA
rs1044831	G	A	-0.05179	0.011132	3.31E-06	16384	genus.Streptococcus.id.1853.s	TRUE	reported	wZL5zu	NA
rs7916711	G	A	0.102891	0.021736	2.72E-06	15294	genus.Streptococcus.id.1853.s	TRUE	reported	wZL5zu	NA
rs8191642	A	G	-0.06042	0.013671	8.09E-06	16384	genus.Streptococcus.id.1853.s	TRUE	reported	wZL5zu	NA
rs7148175	G	T	0.093105	0.020795	6.51E-06	14526	genus.Streptococcus.id.1853.s	TRUE	reported	wZL5zu	NA
rs2370083	T	G	-0.08168	0.018585	9.75E-06	16277	genus.Streptococcus.id.1853.s	TRUE	reported	wZL5zu	NA
rs1002856	T	C	-0.09212	0.019188	7.30E-06	16311	genus.Streptococcus.id.1853.s	TRUE	reported	wZL5zu	NA
rs6806351	C	T	-0.06338	0.013665	4.94E-06	15973	genus.Streptococcus.id.1853.s	TRUE	reported	wZL5zu	NA

rs5764674	A	G	-0.09077	0.020034	5.48E-06	15939	genus.Streptococcus.id.1853.s	TRUE	reported	wZL5zu	NA
rs9903102	A	C	-0.07095	0.015527	4.18E-06	16384	genus.Streptococcus.id.1853.s	TRUE	reported	wZL5zu	NA
rs6563952	C	G	0.082734	0.018004	5.82E-06	16387	genus.Streptococcus.id.1853.s	TRUE	reported	wZL5zu	NA
rs1263822	C	G	-0.05573	0.010855	2.48E-07	17165	genus.Subdoligranulum.id.2070	TRUE	reported	h7Briv	NA
rs1006532	C	T	-0.05128	0.010811	2.10E-06	17589	genus.Subdoligranulum.id.2070	TRUE	reported	h7Briv	NA
rs4347804	G	A	0.166063	0.035748	2.18E-06	4312	genus.Subdoligranulum.id.2070	TRUE	reported	h7Briv	NA
rs6555306	C	T	-0.07408	0.015546	2.81E-06	17591	genus.Subdoligranulum.id.2070	TRUE	reported	h7Briv	NA
rs3761728	G	T	-0.05435	0.011887	3.87E-06	17570	genus.Subdoligranulum.id.2070	TRUE	reported	h7Briv	NA
rs2114677	T	C	-0.10418	0.023083	2.72E-06	15566	genus.Subdoligranulum.id.2070	TRUE	reported	h7Briv	NA
rs7515821	C	T	-0.07234	0.015933	7.52E-06	17165	genus.Subdoligranulum.id.2070	TRUE	reported	h7Briv	NA
rs2171249	T	C	0.106744	0.023321	4.51E-06	14679	genus.Subdoligranulum.id.2070	TRUE	reported	h7Briv	NA
rs3594063	A	G	-0.0511	0.011001	4.22E-06	17582	genus.Subdoligranulum.id.2070	TRUE	reported	h7Briv	NA
rs7652831	T	G	-0.14328	0.031066	7.41E-06	8468	genus.Subdoligranulum.id.2070	TRUE	reported	h7Briv	NA
rs1667315	A	G	0.048502	0.010745	6.72E-06	17586	genus.Subdoligranulum.id.2070	TRUE	reported	h7Briv	NA
rs7666426	A	T	0.083408	0.018536	4.87E-06	16712	genus.Subdoligranulum.id.2070	TRUE	reported	h7Briv	NA
rs1049783	T	C	-0.05241	0.01187	8.38E-06	17579	genus.Subdoligranulum.id.2070	TRUE	reported	h7Briv	NA
rs1696243	T	A	0.0856	0.018921	7.65E-06	15857	genus.Subdoligranulum.id.2070	TRUE	reported	h7Briv	NA
rs1317303	G	A	-0.07181	0.015163	2.73E-06	13245	genus.Sutterella.id.2896.summ	TRUE	reported	s9jqKj	NA
rs2321387	A	G	-0.05929	0.012451	1.87E-06	13252	genus.Sutterella.id.2896.summ	TRUE	reported	s9jqKj	NA
rs7499539	G	A	0.061748	0.0131	2.36E-06	13254	genus.Sutterella.id.2896.summ	TRUE	reported	s9jqKj	NA
rs6250147	A	G	0.069423	0.014942	5.52E-06	12762	genus.Sutterella.id.2896.summ	TRUE	reported	s9jqKj	NA
rs1434387	C	T	-0.14579	0.030687	3.28E-06	9368	genus.Sutterella.id.2896.summ	TRUE	reported	s9jqKj	NA
rs9350083	G	T	-0.05931	0.013393	8.23E-06	13254	genus.Sutterella.id.2896.summ	TRUE	reported	s9jqKj	NA
rs7638039	C	T	0.064562	0.014389	8.66E-06	13236	genus.Sutterella.id.2896.summ	TRUE	reported	s9jqKj	NA
rs1159162	G	T	-0.06884	0.015137	6.50E-06	12604	genus.Sutterella.id.2896.summ	TRUE	reported	s9jqKj	NA
rs2613606	T	C	-0.05568	0.012412	7.20E-06	13249	genus.Sutterella.id.2896.summ	TRUE	reported	s9jqKj	NA
rs1145877	G	A	-0.07355	0.016241	7.20E-06	13251	genus.Sutterella.id.2896.summ	TRUE	reported	s9jqKj	NA
rs607327	T	C	0.057833	0.012905	6.63E-06	13251	genus.Sutterella.id.2896.summ	TRUE	reported	s9jqKj	NA
rs2050185	A	G	0.057514	0.012877	7.97E-06	13243	genus.Sutterella.id.2896.summ	TRUE	reported	s9jqKj	NA
rs1883097	T	C	0.226399	0.045464	4.16E-07	4417	genus.Terrisporobacter.id.113	TRUE	reported	4YOMU3	NA
rs2569953	C	A	-0.07756	0.017464	8.95E-06	6637	genus.Terrisporobacter.id.113	TRUE	reported	4YOMU3	NA
rs7184125	C	T	0.091256	0.020549	8.48E-06	6232	genus.Terrisporobacter.id.113	TRUE	reported	4YOMU3	NA
rs2872237	A	C	-0.08145	0.017594	3.97E-06	6632	genus.Terrisporobacter.id.113	TRUE	reported	4YOMU3	NA

rs5840543	T	G	0.134617	0.030118	7.94E-06	6176	genus.Terrisporobacter.id.113	TRUE	reported	4YOMU3	NA
rs7034891	C	G	-0.07992	0.017379	4.54E-06	6629	genus.Terrisporobacter.id.113	TRUE	reported	4YOMU3	NA
rs1497445	G	A	0.169883	0.031548	7.01E-08	8459	genus.Turicibacter.id.2162.su	TRUE	reported	20jGcS	NA
rs4869133	A	G	0.131186	0.027197	2.55E-06	8256	genus.Turicibacter.id.2162.su	TRUE	reported	20jGcS	NA
rs1260336	C	T	0.110861	0.02256	8.67E-07	8923	genus.Turicibacter.id.2162.su	TRUE	reported	20jGcS	NA
rs1105468	C	T	-0.10475	0.0227	2.31E-06	8928	genus.Turicibacter.id.2162.su	TRUE	reported	20jGcS	NA
rs5575621	C	T	-0.11512	0.024071	2.81E-06	8941	genus.Turicibacter.id.2162.su	TRUE	reported	20jGcS	NA
rs6126517	C	G	-0.08586	0.018578	4.14E-06	8941	genus.Turicibacter.id.2162.su	TRUE	reported	20jGcS	NA
rs7199484	A	G	-0.07314	0.016017	5.77E-06	8948	genus.Turicibacter.id.2162.su	TRUE	reported	20jGcS	NA
rs1164945	C	G	0.095089	0.020343	3.27E-06	8943	genus.Turicibacter.id.2162.su	TRUE	reported	20jGcS	NA
rs2834977	C	T	-0.096	0.020826	3.96E-06	8946	genus.Turicibacter.id.2162.su	TRUE	reported	20jGcS	NA
rs2952020	A	G	-0.0759	0.016576	5.63E-06	8949	genus.Turicibacter.id.2162.su	TRUE	reported	20jGcS	NA
rs1166653	T	C	-0.11169	0.024844	7.37E-06	8256	genus.Turicibacter.id.2162.su	TRUE	reported	20jGcS	NA
rs4247078	G	C	0.071038	0.015522	5.46E-06	8948	genus.Turicibacter.id.2162.su	TRUE	reported	20jGcS	NA
rs2221441	C	G	0.071036	0.015343	3.46E-06	8720	genus.Turicibacter.id.2162.su	TRUE	reported	20jGcS	NA
rs3734633	A	G	-0.12096	0.02683	5.32E-06	8626	genus.Turicibacter.id.2162.su	TRUE	reported	20jGcS	NA
rs6747674	G	T	0.132164	0.022208	3.74E-09	4948	genus.Tyzzerella3.id.11335.su	TRUE	reported	e418Br	NA
rs1770627	C	T	-0.14037	0.027471	5.88E-07	5395	genus.Tyzzerella3.id.11335.su	TRUE	reported	e418Br	NA
rs7561370	C	T	0.131341	0.028629	1.52E-06	5396	genus.Tyzzerella3.id.11335.su	TRUE	reported	e418Br	NA
rs5579912	G	A	-0.11435	0.02386	1.34E-06	5362	genus.Tyzzerella3.id.11335.su	TRUE	reported	e418Br	NA
rs1780915	T	A	-0.16382	0.033639	1.54E-06	4621	genus.Tyzzerella3.id.11335.su	TRUE	reported	e418Br	NA
rs7019909	C	T	0.144156	0.030163	1.76E-06	5396	genus.Tyzzerella3.id.11335.su	TRUE	reported	e418Br	NA
rs7509180	T	G	-0.18497	0.038303	1.71E-06	4621	genus.Tyzzerella3.id.11335.su	TRUE	reported	e418Br	NA
rs4904512	C	T	-0.11715	0.025031	3.09E-06	5396	genus.Tyzzerella3.id.11335.su	TRUE	reported	e418Br	NA
rs7333521	C	T	-0.20719	0.045312	4.88E-06	4335	genus.Tyzzerella3.id.11335.su	TRUE	reported	e418Br	NA
rs1121022	G	A	-0.21635	0.047758	6.18E-06	3857	genus.Tyzzerella3.id.11335.su	TRUE	reported	e418Br	NA
rs1232220	T	G	-0.14387	0.031828	7.91E-06	5000	genus.Tyzzerella3.id.11335.su	TRUE	reported	e418Br	NA
rs191093	A	G	0.159008	0.035331	6.76E-06	4621	genus.Tyzzerella3.id.11335.su	TRUE	reported	e418Br	NA
rs1089879	T	C	0.12238	0.027468	8.85E-06	5387	genus.Tyzzerella3.id.11335.su	TRUE	reported	e418Br	NA
rs6920448	T	C	-0.14108	0.030545	4.15E-06	5010	genus.Tyzzerella3.id.11335.su	TRUE	reported	e418Br	NA
rs2013594	C	T	-0.07207	0.015515	3.42E-06	9283	genus.Veillonella.id.2198.sum	TRUE	reported	21Ds3L	NA
rs1267970	G	C	-0.07935	0.016482	1.78E-06	9290	genus.Veillonella.id.2198.sum	TRUE	reported	21Ds3L	NA
rs742016	G	A	-0.06886	0.014977	4.66E-06	9279	genus.Veillonella.id.2198.sum	TRUE	reported	21Ds3L	NA

rs1882878	G	A	-0.0769	0.016391	2.98E-06	9282	genus.Veillonella.id.2198.sum	TRUE	reported	21Ds3L	NA
rs7645873	T	A	0.076139	0.016423	3.12E-06	9286	genus.Veillonella.id.2198.sum	TRUE	reported	21Ds3L	NA
rs5580741	G	A	0.107329	0.023763	5.51E-06	8610	genus.Veillonella.id.2198.sum	TRUE	reported	21Ds3L	NA
rs6237642	T	C	-0.07622	0.01635	3.65E-06	8999	genus.Veillonella.id.2198.sum	TRUE	reported	21Ds3L	NA
rs7359080	A	C	-0.13539	0.030352	7.40E-06	6189	genus.Veillonella.id.2198.sum	TRUE	reported	21Ds3L	NA
rs1114149	A	G	-0.07834	0.017439	9.75E-06	9291	genus.Veillonella.id.2198.sum	TRUE	reported	21Ds3L	NA
rs6656807	G	A	0.070306	0.015395	5.50E-06	8999	genus.Veillonella.id.2198.sum	TRUE	reported	21Ds3L	NA
rs1161453	C	G	0.074454	0.016521	7.13E-06	8411	genus.Veillonella.id.2198.sum	TRUE	reported	21Ds3L	NA
rs5634919	G	A	-0.1585	0.031523	6.26E-07	3266	genus.Victivallis.id.2256.sum	TRUE	reported	NnhhJu	NA
rs592514	A	T	-0.18145	0.039168	2.60E-06	3241	genus.Victivallis.id.2256.sum	TRUE	reported	NnhhJu	NA
rs911666	C	T	-0.11858	0.026315	7.65E-06	3267	genus.Victivallis.id.2256.sum	TRUE	reported	NnhhJu	NA
rs7374366	A	G	-0.18227	0.040504	7.23E-06	3235	genus.Victivallis.id.2256.sum	TRUE	reported	NnhhJu	NA
rs4764863	A	G	0.121561	0.024606	8.22E-07	3266	genus.Victivallis.id.2256.sum	TRUE	reported	NnhhJu	NA
rs1251254	C	A	-0.17804	0.037434	2.54E-06	3239	genus.Victivallis.id.2256.sum	TRUE	reported	NnhhJu	NA
rs342302	G	A	-0.15275	0.035157	8.16E-06	3265	genus.Victivallis.id.2256.sum	TRUE	reported	NnhhJu	NA
rs4895919	C	T	-0.11692	0.024752	2.75E-06	3254	genus.Victivallis.id.2256.sum	TRUE	reported	NnhhJu	NA
rs1189994	A	G	0.130568	0.027624	2.77E-06	3258	genus.Victivallis.id.2256.sum	TRUE	reported	NnhhJu	NA
rs6445926	C	G	0.117031	0.024976	2.96E-06	3267	genus.Victivallis.id.2256.sum	TRUE	reported	NnhhJu	NA
rs173120	C	T	0.13381	0.029013	7.65E-06	3263	genus.Victivallis.id.2256.sum	TRUE	reported	NnhhJu	NA
rs2546432	C	T	-0.1108	0.024965	9.93E-06	3266	genus.Victivallis.id.2256.sum	TRUE	reported	NnhhJu	NA
rs1882775	G	A	-0.13827	0.031273	8.73E-06	3258	genus.Victivallis.id.2256.sum	TRUE	reported	NnhhJu	NA

sure
