# Supplementary material for: Bidirectional Two-Sample, Two-Step Mendelian Randomisation Study Reveals Mediating Role of Gut Microbiota Between Vitamin B Supplementation and Alzheimer’s Disease
Source: Nutrients. 2024 Nov 18;16(22):3929. doi: 10.3390/nu16223929 (PMC11597120; doi:10.3390/nu16223929)
Supplement: Supplementary file 1 [file nutrients-16-03929-s001.zip › Table S2.pdf]

Table S2 MR estimates for the association between gut microbiota and Alzheimer

Bacterial taxa (exposure)	MR method	No. of SNP	OR	*lo95% CI*	*up95%*	*P*-value	*q*-value
LachnospiraceaeNK4A136group	MR Egger	15	0.999219	0.997402	1.001039	0.415140	0.742685
	Weighted median	15	0.999440	0.998298	1.000583	0.336703	0.700683
	Inverse variance weighted	15	0.999045	0.998195	0.999897	0.028002	0.162959
	Simple mode	15	0.999742	0.998036	1.001450	0.771234	0.842818
	Weighted mode	15	0.999559	0.998153	1.000966	0.548776	0.792333
Paraprevotella	MR Egger	12	0.999492	0.997458	1.001530	0.635439	0.435756
	Weighted median	12	0.999140	0.998294	0.999987	0.046699	0.064049
	Inverse variance weighted	12	0.999268	0.998634	0.999902	0.023634	0.064049
	Simple mode	12	0.999026	0.997727	1.000327	0.170317	0.171496
	Weighted mode	12	0.999083	0.997851	1.000317	0.173300	0.173978
Slackia	MR Egger	6	1.002058	0.996727	1.007418	0.492005	0.242131
	Weighted median	6	0.999083	0.997933	1.000234	0.118434	0.091601
	Inverse variance weighted	6	0.999032	0.998145	0.999920	0.032544	0.064063
	Simple mode	6	0.998327	0.996431	1.000226	0.144817	0.094402
	Weighted mode	6	0.998354	0.996382	1.000330	0.163339	0.095895
Bifidobacterium	MR Egger	19	0.998638	0.996637	1.000644	0.200799	0.410853
	Weighted median	19	0.998864	0.997854	0.999876	0.027857	0.088213
	Inverse variance weighted	19	0.998883	0.998193	0.999574	0.001532	0.005949
	Simple mode	19	0.999672	0.997746	1.001601	0.742412	0.720544
	Weighted mode	19	0.998067	0.996339	0.999798	0.042098	0.127556
Defluviitaleaceae UCG-011	MR Egger	8	1.002823	1.000020	1.005634	0.095861	0.128200
	Weighted median	8	1.000738	0.999624	1.001854	0.194291	0.182662
	Inverse variance weighted	8	1.000997	1.000145	1.001849	0.021779	0.058253
	Simple mode	8	1.000046	0.998126	1.001970	0.963626	0.525710
	Weighted mode	8	1.000157	0.998358	1.001960	0.868992	0.499891
Desulfovibrio	MR Egger	11	1.001044	0.998099	1.003998	0.505244	0.255300
	Weighted median	11	1.001389	1.000182	1.002597	0.024114	0.024370
	Inverse variance weighted	11	1.001223	1.000274	1.002172	0.011482	0.023207
	Simple mode	11	1.002274	0.999952	1.004602	0.083872	0.056507

	Weighted mode	11	1.002237	0.999656	1.004825	0.120245	0.075435
RuminococcaceaeUCG003	MR Egger	11	1.003612	1.000547	1.006687	0.046196	0.047751
	Weighted median	11	1.000988	0.999689	1.002288	0.136071	0.089281
	Inverse variance weighted	11	1.000970	1.000006	1.001934	0.048517	0.047751
	Simple mode	11	1.000779	0.998691	1.002871	0.481511	0.238180
	Weighted mode	11	1.000816	0.998576	1.003061	0.491976	0.242103
Ruminococcusgnavusgroup	MR Egger	11	1.002774	0.999937	1.005619	0.087587	0.052838
	Weighted median	11	1.000754	0.999840	1.001670	0.105924	0.052838
	Inverse variance weighted	11	1.000699	1.000045	1.001354	0.036314	0.052838
	Simple mode	11	1.000829	0.999303	1.002358	0.312193	0.117661
	Weighted mode	11	1.000816	0.999248	1.002386	0.331772	0.124124
