# Supplementary material for: Bidirectional Two-Sample, Two-Step Mendelian Randomisation Study Reveals Mediating Role of Gut Microbiota Between Vitamin B Supplementation and Alzheimer’s Disease
Source: Nutrients. 2024 Nov 18;16(22):3929. doi: 10.3390/nu16223929 (PMC11597120; doi:10.3390/nu16223929)
Supplement: Supplementary file 1 [file nutrients-16-03929-s001.zip › Table S3.pdf]

Table S3 The F-statistic for each SNP

Bacterial taxa (exposure)	SNP	Effect allele	Other allele	MAF	Exposure (Bacteria)			
					Beta	SE	P-value	F-statistic
Defluviitaleaceae (UCG011)	rs72731813	C	T	0.048	-0.147	0.029	4.33E-07	25.69441141
Defluviitaleaceae (UCG011)	rs4677103	A	G	0.18	0.098	0.02	9.60E-07	24.01
Defluviitaleaceae (UCG011)	rs112893842	T	C	0.09	0.114	0.023	1.45E-06	24.56710775
Defluviitaleaceae (UCG011)	rs12122999	T	G	0.2247	-0.0827292	0.018115427	4.83E-06	20.85549484
Defluviitaleaceae (UCG011)	rs55658617	T	C	0.036	0.174	0.036	2.15E-06	23.36111111
Defluviitaleaceae (UCG011)	rs9608282	T	G	0.033	0.143	0.03	2.52E-06	22.72111111
Defluviitaleaceae (UCG011)	rs9725395	A	G	0.114	-0.138	0.03	3.52E-06	21.16
Defluviitaleaceae (UCG011)	rs4344384	G	T	0.496	0.072	0.016	4.83E-06	20.25
Lachnospiraceae (NK4A136 group)	rs954878	A	G	0.373	-0.052	0.011	1.78E-06	22.34710744
Lachnospiraceae (NK4A136 group)	rs160061	A	G	0.519	0.051	0.011	2.12E-06	21.49586777
Lachnospiraceae (NK4A136 group)	rs68104925	T	C	0.305	-0.055	0.012	2.37E-06	21.00694444
Lachnospiraceae (NK4A136 group)	rs7616165	G	T	0.025	-0.231	0.048	2.77E-06	23.16015625
Lachnospiraceae (NK4A136 group)	rs76193507	A	G	0.086	-0.23	0.05	2.93E-06	21.16
Lachnospiraceae (NK4A136 group)	rs7832116	A	G	0.129	-0.071	0.015	3.57E-06	22.40444444
Lachnospiraceae (NK4A136 group)	rs73044693	A	G	0.071	-0.108	0.023	3.57E-06	22.04914934
Lachnospiraceae (NK4A136 group)	rs11263806	A	G	0.343	-0.052	0.012	5.07E-06	18.77777778
Lachnospiraceae (NK4A136 group)	rs7073658	T	G	0.485	-0.05	0.011	5.27E-06	20.66115702
Lachnospiraceae (NK4A136 group)	rs2880566	T	C	0.148	0.06	0.013	5.61E-06	21.30177515
Lachnospiraceae (NK4A136 group)	rs12611395	A	G	0.106	-0.09	0.02	5.83E-06	20.25
Lachnospiraceae (NK4A136 group)	rs4955932	T	C	0.388	-0.049	0.011	7.05E-06	19.84297521
Lachnospiraceae (NK4A136 group)	rs10952110	G	T	0.445	0.049	0.011	9.08E-06	19.84297521
Lachnospiraceae (NK4A136 group)	rs28540839	A	C	0.475	0.051	0.011	9.34E-06	21.49586777

Paraprevotella	rs2081023	A	G	0.142	-0.123	0.024	2.64E-07	26.265625
Paraprevotella	rs9602779	A	C	0.242	-0.107	0.022	6.93E-07	23.65495868
Paraprevotella	rs9900242	A	G	0.343	-0.085	0.018	1.14E-06	22.29938272
Paraprevotella	rs17785622	A	G	0.039	0.248	0.052	1.93E-06	22.74556213
Paraprevotella	rs140997932	T	C	0.055	-0.162	0.035	2.11E-06	21.42367347
Paraprevotella	rs4767113	C	T	0.328	0.088	0.018	2.14E-06	23.90123457
Paraprevotella	rs4756632	G	T	0.13	-0.139	0.029	3.82E-06	22.97384067
Paraprevotella	rs145020347	A	G	0.148	-0.125	0.026	4.03E-06	23.11390533
Paraprevotella	rs3008582	T	C	0.194	0.106	0.023	4.36E-06	21.24007561
Paraprevotella	rs3801748	G	A	0.362	0.078	0.017	5.20E-06	21.05190311
Paraprevotella	rs7240324	T	G	0.246	-0.102	0.023	5.96E-06	19.66729679
Paraprevotella	rs10842464	T	C	0.302	-0.076	0.017	6.60E-06	19.98615917
Paraprevotella	rs17109926	A	G	0.271	-0.099	0.022	6.75E-06	20.25
Parasutterella	rs7572229	G	A	0.522	0.066	0.013	6.32E-07	25.77514793
Parasutterella	rs10899911	A	G	0.235	-0.072	0.015	1.15E-06	23.04
Parasutterella	rs7303158	C	T	0.448	0.065	0.013	1.33E-06	25
Parasutterella	rs78383039	T	C	0.04	-0.146	0.03	1.57E-06	23.68444444
Parasutterella	rs6828768	C	T	0.468	0.064	0.013	1.78E-06	24.23668639
Parasutterella	rs55877868	A	C	0.1	-0.104	0.023	2.87E-06	20.44612476
Parasutterella	rs2090816	A	C	0.186	0.084	0.018	2.90E-06	21.77777778
Parasutterella	rs35055552	T	C	0.14	0.11	0.024	3.35E-06	21.00694444
Parasutterella	rs8039785	T	G	0.512	0.062	0.013	3.62E-06	22.74556213
Parasutterella	rs823424	G	A	0.254	-0.071	0.016	4.95E-06	19.69140625
Parasutterella	rs62273907	A	G	0.067	0.229	0.05	5.88E-06	20.9764
Parasutterella	rs7311004	T	C	0.444	-0.062	0.014	5.92E-06	19.6122449
Parasutterella	rs11715853	G	A	0.295	-0.066	0.015	6.23E-06	19.36

Parasutterella	rs6809952	G	A	0.278	-0.068	0.015	8.13E-06	20.55111111
Slackia	rs8901	C	T	0.292	0.093	0.019	6.07E-07	23.95844875
Slackia	rs4492265	A	G	0.304	-0.091	0.019	2.41E-06	22.93905817
Slackia	rs12440440	A	G	0.342	0.09	0.019	2.63E-06	22.43767313
Slackia	rs16894137	C	T	0.131	-0.123	0.026	2.71E-06	22.38017751
Slackia	rs10409783	A	G	0.291	0.095	0.021	7.70E-06	20.46485261
Slackia	rs35156985	T	C	0.042	-0.156	0.035	8.06E-06	19.86612245
Bifidobacterium	rs182549	C	T	0.401	0.12	0.013	1.28E-20	85.20710059
Bifidobacterium	rs7322849	T	C	0.091	0.112	0.02	1.08E-08	31.36
Bifidobacterium	rs62181700	G	A	0.247	-0.062	0.013	2.17E-06	22.74556213
Bifidobacterium	rs56108664	T	C	0.13	0.073	0.016	2.44E-06	20.81640625
Bifidobacterium	rs857444	C	T	0.369	0.056	0.012	3.57E-06	21.77777778
Bifidobacterium	rs75344046	C	T	0.047	0.232	0.051	4.86E-06	20.69357939
Bifidobacterium	rs540489	T	G	0.176	-0.064	0.014	5.19E-06	20.89795918
Bifidobacterium	rs55888705	A	G	0.28	0.055	0.012	6.67E-06	21.00694444
Bifidobacterium	rs2686790	T	C	0.148	0.071	0.016	7.50E-06	19.69140625
Bifidobacterium	rs11208411	T	C	0.84	0.071	0.016	7.57E-06	19.69140625
Bifidobacterium	rs12022129	G	A	0.275	0.062	0.014	8.00E-06	19.6122449
Bifidobacterium	rs2491158	G	A	0.133	0.071	0.016	8.05E-06	19.69140625
Bifidobacterium	rs5746486	T	C	0.388	-0.054	0.012	9.00E-06	20.25
Desulfovibrio	rs16863365	A	G	0.047	0.109	0.023	1.79E-06	22.45935728
Desulfovibrio	rs2853179	C	T	0.23	0.081	0.017	2.42E-06	22.70242215
Desulfovibrio	rs13066142	G	A	0.091	0.119	0.025	3.79E-06	22.6576
Desulfovibrio	rs6580353	T	C	0.204	0.077	0.017	4.94E-06	20.51557093
Desulfovibrio	rs4797774	G	A	0.043	0.213	0.047	5.64E-06	20.5382526
Desulfovibrio	rs12031543	T	C	0.138	-0.127	0.028	6.55E-06	20.57270408

Desulfovibrio	rs2590913	G	A	0.052	0.154	0.034	6.65E-06	20.51557093
Desulfovibrio	rs72647089	T	G	0.081	-0.107	0.024	8.30E-06	19.87673611
Desulfovibrio	rs2032031	A	G	0.509	-0.065	0.015	9.14E-06	18.77777778
Desulfovibrio	rs7729080	C	A	0.293	-0.07	0.016	9.96E-06	19.140625
Ruminococcaceae (UCG003)	rs73341549	T	C	0.057	-0.17	0.032	1.51E-07	28.22265625
Ruminococcaceae (UCG003)	rs646327	G	A	0.409	0.059	0.012	7.83E-07	24.17361111
Ruminococcaceae (UCG003)	rs6759615	A	G	0.098	0.103	0.02	7.86E-07	26.5225
Ruminococcaceae (UCG003)	rs11613919	G	T	0.221	0.073	0.016	1.63E-06	20.81640625
Ruminococcaceae (UCG003)	rs11243416	T	C	0.07	-0.093	0.019	1.67E-06	23.95844875
Ruminococcaceae (UCG003)	rs16959793	A	C	0.416	-0.063	0.013	2.22E-06	23.4852071
Ruminococcaceae (UCG003)	rs4452755	A	C	0.339	-0.063	0.013	3.29E-06	23.4852071
Ruminococcaceae (UCG003)	rs10490280	C	T	0.189	-0.067	0.014	4.16E-06	22.90306122
Ruminococcaceae (UCG003)	rs3013089	G	A	0.375	-0.055	0.012	4.38E-06	21.00694444
Ruminococcaceae (UCG003)	rs4532474	G	A	0.163	0.077	0.017	4.82E-06	20.51557093
Ruminococcaceae (UCG003)	rs2523124	T	C	0.402	-0.055	0.012	5.78E-06	21.00694444
Eubacterium (ventriosum group)	rs11617697	A	G	0.056	-0.143	0.029	7.22E-07	24.31510107
Eubacterium (ventriosum group)	rs57199565	T	C	0.199	0.078	0.016	7.97E-07	23.765625
Eubacterium (ventriosum group)	rs73615400	T	C	0.098	-0.096	0.019	9.54E-07	25.52908587
Eubacterium (ventriosum group)	rs16884680	G	T	0.104	-0.091	0.019	1.74E-06	22.93905817
Eubacterium (ventriosum group)	rs12964517	G	A	0.283	0.059	0.012	2.07E-06	24.17361111
Eubacterium (ventriosum group)	rs876734	C	T	0.283	-0.062	0.013	2.89E-06	22.74556213
Eubacterium (ventriosum group)	rs78250280	G	A	0.14	0.075	0.016	3.36E-06	21.97265625
Eubacterium (ventriosum group)	rs3809430	T	C	0.308	-0.055	0.012	3.55E-06	21.00694444
Eubacterium (ventriosum group)	rs73849225	T	C	0.083	0.098	0.022	5.21E-06	19.84297521
Eubacterium (ventriosum group)	rs35179274	C	T	0.178	-0.063	0.014	5.76E-06	20.25
Eubacterium (ventriosum group)	rs66746423	C	T	0.153	0.075	0.016	6.11E-06	21.97265625

Eubacterium (ventriosum group)	rs72783037	C	A	0.206	0.066	0.014	6.55E-06	22.2244898
Eubacterium (ventriosum group)	rs6704822	A	G	0.13	0.074	0.017	6.62E-06	18.94809689
Eubacterium (ventriosum group)	rs9316536	T	G	0.145	-0.082	0.018	7.84E-06	20.75308642
