# Supplementary material for: Bidirectional Two-Sample, Two-Step Mendelian Randomisation Study Reveals Mediating Role of Gut Microbiota Between Vitamin B Supplementation and Alzheimer’s Disease
Source: Nutrients. 2024 Nov 18;16(22):3929. doi: 10.3390/nu16223929 (PMC11597120; doi:10.3390/nu16223929)
Supplement: Supplementary file 1 [file nutrients-16-03929-s001.zip › Table S4.pdf]

Table S4 The heterogeneity of gut microbiota instrumental variables.

Bacterial taxa (exposure)	Cochran's Q	df	P-value
LachnospiraceaeNK4A136group	15.94684696	14	0.316631057
Paraprevotella	6.564454324	11	0.833179356
Slackia	4.748920437	5	0.447284154
Bifidobacterium	19.09572319	18	0.385951249
Defluviitaleaceae UCG-011	7.389030211	7	0.389527731
Desulfovibrio	14.51437571	10	0.150795239
RuminococcaceaeUCG003	7.44650972	10	0.682725522
Ruminococcusgnavusgroup	8.99465974	10	0.532610462

df, degree of freedom.
